# Best practice guidelines for professional nurses to provide self-management support to adults with tuberculosis-human immunodeficiency virus coinfection: A scoping review

**DOI:** 10.1371/journal.pone.0291529

**Published:** 2023-09-12

**Authors:** Eric Tornu, Portia Jordan, Michael McCaul

**Affiliations:** 1 Department of Nursing and Midwifery, Faculty of Medicine and Health Sciences, Stellenbosch University, Cape Town, South Africa; 2 Centre for Evidence-based Health Care, Division of Epidemiology and Biostatistics, Department of Global Health, Stellenbosch University, Cape Town, South Africa; Keele University & University Hospitals of North Midlands (UHNM) NHS Trust, UNITED KINGDOM

## Abstract

**Background:**

Adults with tuberculosis-human immunodeficiency virus coinfection require professional nurses’ support to manage their illness, treatment and its effect on their daily lives. This scoping review maps recommendations in clinical or best practice guidelines that guide professional nurses to provide self-management support to adults with tuberculosis-human immunodeficiency virus coinfection in primary healthcare settings.

**Methods:**

We conducted a scoping review by searching for guidelines in six online databases, guideline clearing houses and search engines from 16th April 2022 to 25th May 2022. The title, abstract and full-text screening of guidelines were conducted independently and in duplicate by two reviewers based on predetermined eligibility criteria. The guidelines were critically appraised with the Appraisal of Guidelines Research and Evaluation (AGREE) II instrument. Relevant data regarding the characteristics of the guideline, recommendations and underlying evidence were extracted, analysed and reported.

**Results:**

The six guidelines on self-management support found were developed in four high-income countries. Five of the guidelines recorded <60% across all six domains of the AGREE II instrument. One high-quality guideline scored >60% in all AGREE II domains but was informed by outdated evidence produced between 1977 to 2010. Twenty-five practice, education and organisational/policy recommendations were extracted from the high-quality guideline. The guidelines did not report evidence-to-decision frameworks and the strength of the recommendations. The guidelines also lacked direct underlying evidence on the effectiveness and cost of self-management support. Lastly, the review found a paucity of contextual (equity, acceptability and feasibility) evidence on self-management support among adults with tuberculosis-human immunodeficiency virus in the guidelines.

**Conclusion:**

There is a dearth of updated and relevant high-quality guidelines that guide healthcare professionals to provide self-management support to adults with tuberculosis-human immunodeficiency virus coinfection in primary healthcare settings. Systematic reviews of effectiveness, economic and contextual evidence related to self-management support interventions are required for guideline production.

## Introduction

Tuberculosis-Human Immunodeficiency Virus (TB-HIV) coinfection is a significant health challenge facing many adults (aged 18 years and older) globally [1]. The TB-HIV chronic condition arises when an individual is simultaneously infected with TB bacillus and HIV. Adults with TB-HIV coinfection (AWTB-HIV) experience the negative impact of TB-HIV coinfection on the physical, psychological and social aspects of their lives. Through self-management, the AWTB-HIV can identify how to manage their medical (symptoms and health promotion), role (relationships and life-role) and emotional (stigma and self-esteem) challenges as well as their long-term antituberculosis and antiretroviral treatment [2, 3].

Self-management entails the tasks people living with chronic conditions (such as TB-HIV coinfection) undertake to manage their illness and its effect on their daily life [2]. Previous studies indicate that self-management can contribute to an improvement in medication adherence as well as psychological (self-efficacy or self-confidence) and social (social support) health-related quality of life of persons living with chronic conditions such as TB and HIV [4–8]. Self-management support refers to the collaborative interaction between an individual with a chronic condition and care providers to improve the management of the chronic condition [9]. Thus, self-management support presents an opportunity for professional nurses to collaborate with AWTB-HIV to self-manage their challenges and prevent illnesses or death. The self-management support provided by professional nurses could be in the form of health education or counselling, skills training, collaborative goal-setting, action planning, problem-solving and regular assessment of progress and health problems [9, 10].

Professional nurses provide care (including self-management support) to AWTB-HIV in primary healthcare settings such as TB or HIV clinics globally [11]. Primary healthcare delivery focuses on ensuring people’s well-being by addressing their health needs through the provision of health services which are accessible within their environment [12]. The primary health care services delivered to AWTB-HIV in TB and HIV clinics are mainly out-patient health services which include monitoring the health status of AWTB-HIV, counselling the AWTB-HIV and their relations, administering TB and HIV treatment and arranging follow-up clinic visits [13]. The professional nurses’ access to and utilisation of high-quality guidelines relevant to their context can contribute to improving the quality of care they provide to their patients, such as AWTB-HIV within the primary healthcare setting [14].

In this paper, *best practice guidelines* also refer to *clinical practice guidelines* because of their similarity in meaning. Best practice guidelines are “systematically developed, evidence-based documents that include recommendations for nurses and the interprofessional team, educators, leaders and policymakers, persons and their chosen families on specific clinical and healthy work environment topics” [15]. Similarly, the Institute of Medicine (IOM) defines clinical practice guidelines as “statements that include recommendations intended to optimise patient care that are informed by a systematic review of evidence and an assessment of the benefits and harms of alternative care options” [16]. In both definitions, guidelines offer evidence-based recommendations for providing the best possible health care. In relation to the provision of self-management support, best practice guidelines can present recommendations for professional nurses to provide the best possible self-management support to AWTB-HIV to improve their health outcomes.

Even though best practice guidelines offer numerous advantages for improved patient care, their methodological quality can vary based on the quality, type and age of the evidence informing the recommendations [17]. With the aid of a guideline appraisal instrument such as the Appraisal of Guidelines Research and Evaluation (AGREE) II, the quality of existing best practice guidelines can be appraised before use [18]. The AGREE II instrument items also aid guideline appraisers in rating whether a guideline is recommended for use as it is or requires modifications before use in care delivery [18]. Consequently, a review and appraisal of best practice guidelines on self-management support can be a valuable resource for guideline developers to adapt existing self-management support guidelines for their local contexts. Guideline adaptation refers to modifying the recommendations of existing best practice guidelines by including local research evidence and expert group consensus to address local issues [19, 20]. Guideline adaptation can help to reduce the time and effort required to produce context-relevant recommendations which guide healthcare delivery [19, 20]. A reliable guideline adaptation process, however, requires that existing recommendations are mapped and informed by effectiveness evidence (benefits and harms of recommended interventions), economic evidence (cost/resource implications) and contextual evidence (equity, acceptability and feasibility) which are direct or specific to the population (or patients) for whom the guideline will be used [21, 22].

Previous studies have mapped recommendations and critically assessed the quality of existing guidelines on depression [23, 24], emergency [25], oral [26], and prehospital care [27]. However, no existing reviews have searched and appraised TB-HIV related self-management support best practice guidelines and their recommendations to inform guideline adaptation, guide healthcare, promote education or inform policy. A scoping review was deemed the most appropriate review method as it allows the researchers to map out existing recommendations in guidelines on how professional nurses can provide self-management support to AWTB-HIV. Mapping out the existing self-management support guidelines and their underlying evidence can facilitate the adolopment (development, adoption or adaptation) of best practice guidelines on self-management support. Consequently, this scoping review aims to map and describe best practice guidelines and recommendations that can guide professional nurses to provide self-management support to AWTB-HIV in primary healthcare settings.

## Methods

The scoping review adopted the JBI methodology for scoping reviews using a protocol developed before the review [28, 29]. The Preferred Reporting Items for Systematic Reviews and Meta-Analysis for Scoping Reviews (PRISMA-ScR) checklist guided the reporting of this review, while the PRISMA flow diagram was used to illustrate the guideline search and screening process [30]. The PRISMA-ScR checklist is provided as S1 Checklist.

### Eligibility criteria

The eligibility criteria for the best practice guideline were predetermined using the Participant, Concept and Context Framework (PCC) [31].

#### Participants

This review included best practice guidelines designed for the self-management support of AWTB-HIV, aged 18 years and older, irrespective of gender. Best practice guidelines that guide the provision of self-management support to persons with chronic conditions, in general, were also included in the review to ensure that all relevant recommendations for the provision of self-management support to AWTB-HIV were considered.

#### Concept

The concept or intervention of interest was self-management support provided by professional nurses. Self-management support refers to a professional nurse supporting an AWTB-HIV to undertake the tasks required to self-manage TB-HIV coinfection throughout the period of TB-HIV coinfection. The self-management support could be in the form of health education or counselling, skills training, collaborative goal-setting, action planning, problem-solving and regular assessment of progress and health problems [9, 10].

#### Context

The context included primary health care settings such as TB, HIV or TB-HIV clinics in any country.

#### Outcomes

The outcomes of interest included (1) medication adherence (e.g. indicated by the number of antiretroviral and antituberculosis medications taken by the patient as prescribed), (2) psychological health-related quality of life (e.g. indicated by the patient’s self-assessed self-efficacy) and (3) social health-related quality of life (e.g. indicated by the patient’s self-assessed social support level or sources). Guidelines were included in the review even if they did not report the stated outcomes.

#### Type of sources

This review included best practice guidelines with available full-text versions that provide recommendations. Multi-disciplinary (non-nursing discipline-specific) best practice guidelines were included in the review as self-management support recommendations relevant to professional nurses may be integrated into some guidelines which are not specific to nursing.

#### Exclusion criteria

Best practice guidelines were excluded from the review if they were not in the English language to avoid misinterpretation of the recommendations and the guideline translation costs. Guidance documents such as protocols, algorithms, standard operating procedures and patient care pathways were also excluded since this review considered recommendations based on systematic evidence synthesis. Best practice guidelines designed for in-patient or hospitalised patient care were excluded due to the difference in the care context compared to primary healthcare settings.

### Search strategy

A three-step search strategy was designed with a librarian to search for existing best practice guidelines on self-management support developed from 1st January 2010 to 31st May 2022 [31]. The search was conducted by one reviewer (ET) from 16^th^ April 2022 to 25^th^ May 2022 and checked by two independent reviewers (PJ and MM) to ensure a thorough search for evidence.

Six databases (PubMed, CINAHL, Africa Wide Information, Scopus, Web of Science and Trip) were searched with MeSH and search terms. S1 Appendix presents the search strategy.

We also searched the repositories of 12 guideline clearing houses/organisations, including the National Institute for Health and Care Excellence, Guidelines International Network, Scottish Intercollegiate Guidelines Network and the World Health Organization. Search terms used were comparable to terms used in the database search.

Grey (non-peer-reviewed) literature was also searched on three (3) websites and search engines, including Research Gate, Google and Google Scholar. The first 100 search results were assessed for relevant guidelines during the search in guideline clearing houses and grey literature sources.

An updated search was conducted on 1st August 2022 to identify additional or updated versions of old included guidelines. However, no new or additional guidelines were found for inclusion.

### Selection

After the search, the identified best practice guidelines were exported into Mendeley Reference Manager, and duplicates were removed. Two reviewers (ET and BRO) independently and in duplicate, screened the titles and abstracts of each best practice guideline and removed irrelevant results based on the predetermined eligibility criteria. The potentially relevant best practice guidelines were fully downloaded and exported to the JBI System for the Unified Management, Assessment, and Review of Information (JBI SUMARI; JBI, Adelaide, Australia).

Two reviewers (ET and BRO) independently and in duplicate, examined the full texts of each best practice guideline based on the review’s eligibility criteria. The reasons for excluding full-text best practice guidelines were documented and presented in S2 Appendix. The two reviewers resolved differences regarding the inclusion or exclusion of each guideline during the title/abstract and full-text screen stages through consensus. The included guidelines were examined by four reviewers (ET, PJ, MM and BRO) before data extraction.

### Data extraction

Data extraction was conducted at two levels; (1) at the best practice guidelines’ characteristics level (such as guideline author, year of publication, purpose and setting) and (2) at the recommendations’ characteristics level (such as the number of recommendations and underlying evidence). Two reviewers (ET and BRO) independently extracted the relevant guideline data and recommendations into a Microsoft Excel spreadsheet. Three reviewers (ET, PJ and MM) discussed the extracted data to ensure its accuracy and completeness. The reviewers piloted the data extraction tool to reduce the risk of data extraction errors.

The data extracted for each guideline included the author, date/year of publication, title, target users of the guideline, target beneficiaries (whom the guideline is to be used to care for), context (country and setting), self-management support recommendations and their grading systems. Data extracted about each recommendation within each guideline included the direction and strength of the recommendation, its underlying evidence, evidence-to-decision (EtD) frameworks and systematic review summary of findings tables where available. One reviewer (ET) emailed the guideline authors and requested updated versions, full texts, Grading of Recommendations Assessment, Development and Evaluation (GRADE) EtD frameworks (or similar EtD frameworks or processes) and systematic reviews which may be unavailable in the guidelines found. In response, two guideline development groups provided additional documents for consideration in the review. This measure ensured that all relevant information about the guidelines was obtained for extraction.

### Critical appraisal

The best practice guidelines were critically appraised to identify their methodological quality because recommendations from best practice guidelines with high methodological quality may be adapted to produce contextually appropriate evidence-based guidelines [32].

Two independent reviewers (ET and BRO) used the 23-item AGREE II instrument to appraise the methodological quality of each guideline across six domains (scope and purpose, level of stakeholder involvement, rigour of development, clarity of presentation, applicability and editorial independence) from 0% to 100% [18]. A guideline with a rigour of development domain rating of 60% or more was considered to meet the minimum methodological standards [33].

The two reviewers (ET and BRO) also rated the overall quality of each guideline from 1 (lowest possible quality) to 7 (highest possible quality) and reported the mean score.

### Data analysis

One reviewer (ET) exported data from Microsoft Excel to Statistical Package for the Social Sciences (SPSS) software version 23 for analysis. The results were analysed using descriptive statistics and narrative summary.

## Results

**Fig 1:**
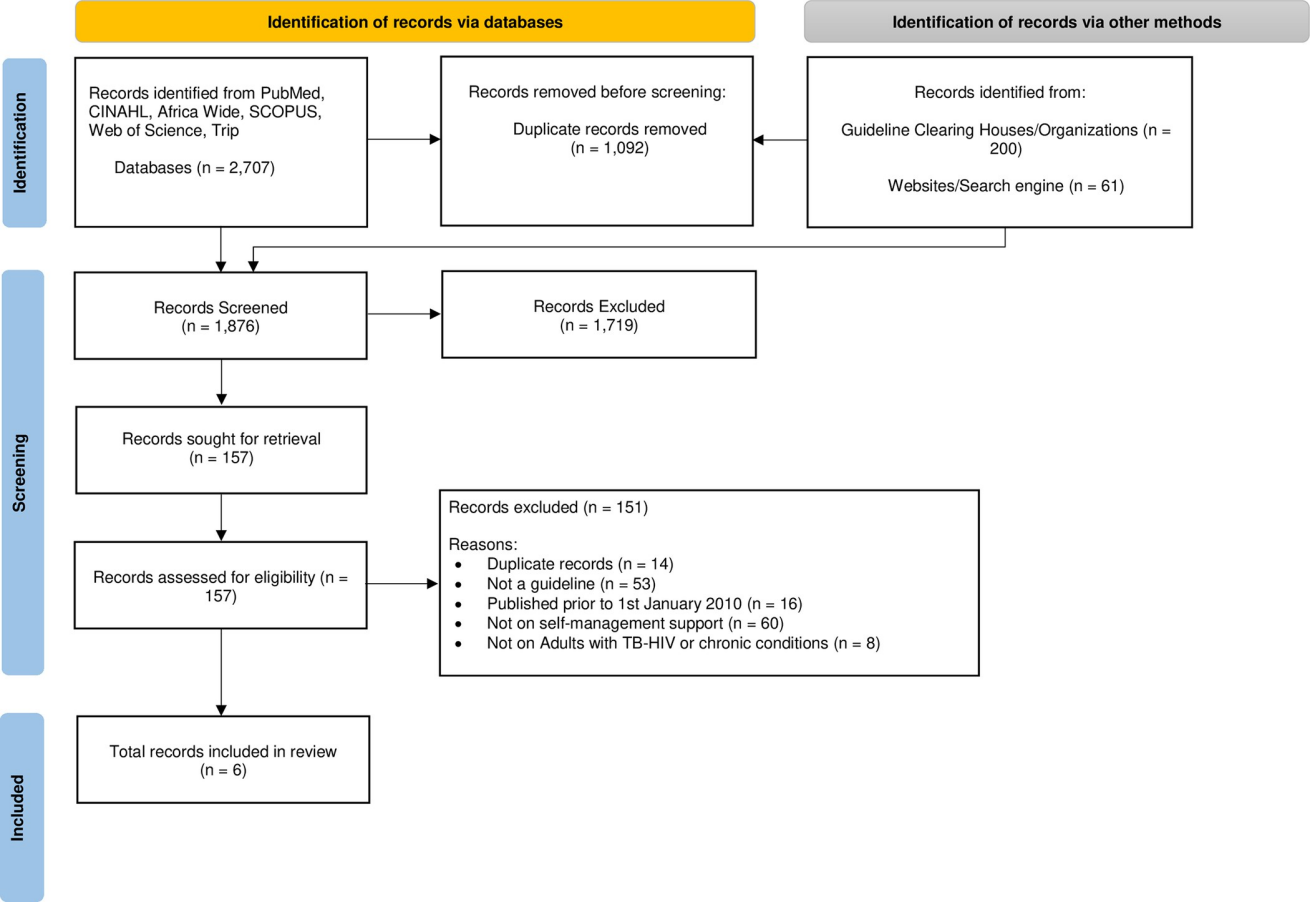
PRISMA flow diagram of literature search and selection.

A total of 2,968 records were identified after the search in six databases and 15 grey literature sources [12 guideline clearing houses/organisations and three websites/search engines]. Six best practice guidelines met the inclusion criteria. The data search, screening and selection process is presented in the PRISMA flow chart (Fig 1).

### Characteristics of included guidelines

The six included guidelines were published by five international organisations namely; the Health Care for the Homeless Clinicians’ Network (HCN) [34], National Health Service (NHS) [35], Registered Nurses’ Association of Ontario (RNAO) [36], The Health Foundation (THF) [37] and The Royal Australian College of General Practitioners (RACGP) which developed two guidelines (RACGP1) [38] and (RACGP2) [39].

The guidelines were published between 2010 [36] and 2020 [35] across four countries; Australia [38, 39], Canada [36], England [35, 37] and the United States of America [34]. All the guidelines were designed for use within the primary healthcare setting by healthcare providers [35, 37], such as nurses [36, 39] and physicians/clinicians [34, 38]. Five guidelines (NHS, RNAO, THF, RACGP1, RACGP2) were developed for the self-management support of persons with chronic conditions in general [34–39]. One guideline (HCN) targeted unstably housed adults and adolescents with HIV [34]. None of the guidelines was developed specifically for the self-management support of AWTB-HIV.

Three guidelines indicated they were summary guidelines; however, complete versions of the guidelines were not found during guideline search or upon email requests to the authors. Five guidelines contained references (NHS, RNAO, THF, RACGP1, RACGP2). The authors of one guideline (NHS) provided additional references when contacted via email. None of the guidelines included a summary of findings table, which reports the characteristics of the underlying evidence for its recommendations. Three guidelines developed by RNAO and RACGP specified the level of evidence and strength of recommendations based on the study design of the underlying evidence [36, 38, 39]. The RNAO graded the level of evidence with an adapted Scottish Intercollegiate Guidelines Network (SIGN) grading system [40]. The RACGP rated the quality of evidence and strength of recommendations with the National Health and Medical Research Council rating system [41]. None of the guidelines reported using the GRADE EtD framework in assessing the certainty of evidence or developing recommendations. None of the guidelines transparently reported the criteria used to make recommendations. Three guidelines disclosed their sources of funding as the Government of Ontario (RNAO) and the Commonwealth Department of Health and Ageing (RACGP1 and RACGP). The characteristics of the included guidelines are summarised in Table 1.

**Table 1:** Characteristics of included national guidelines.

Author (Year)	Guideline Title	Country and Setting for guideline application	Purpose	Target users and beneficiaries	Evidence grading and presentation
Health Care for the Homeless Clinicians’ Network (2013)	Adapting your Health Practice: Treatment and Recommendations for Unstably Housed Patients with HIV/AIDS	United States of America	Not stated. But provides recommendations on how unstably or poorly-housed patients with HIV/AIDS could be supported by care	**Users**: Clinicians	**Criteria for making recommendations:** Absent
		**Setting**: Clinics		**Beneficiaries:** unstably or poorly housed adults and adolescents with HIV	**Summary of findings table**: Absent
					**Certainty of evidence rating**: Absent
					**Strength of recommendation rating**: Absent
National Health Service (2020)	Supported self-management	England	Enable enhanced understanding that health and care professionals have a role in supporting people with long-term conditions to self-manage, alongside more specific interventions including health coaching, self-management education and peer support.	**Users**: Healthcare providers, people and organisations leading local implementation of supported self-management	**Criteria for making recommendations**: Absent
		**Setting**: Primary Healthcare			
					**Summary of findings table**: Absent
					**Certainty of evidence rating**: Absent
					**Strength of recommendation rating**: Absent
				**Beneficiaries:** Persons with chronic conditions	
Registered Nurses’ Association of Ontario (2010)	Strategies to Support Self-Management in Chronic Conditions: Collaboration with Clients	Canada	Provide evidence-based recommendations for Registered Nurses and Registered Practical Nurses in self-management support.	**Users**: Nurses	**Criteria for making recommendations**: Absent
		**Setting**: Primary Healthcare		**Beneficiaries:** Persons with chronic conditions	
					**Summary of findings table**: Absent
					**Certainty of evidence rating**: Adapted SIGN^a^ grading system
					**Strength of Recommendation rating**: Absent
The Health Foundation (2015)	A practical guide to self-management support	United Kingdom	Provide an overview of self-management support and the key components for effective implementation	**Users**: Healthcare professionals, commissioners, service managers, people in voluntary or community groups and patient leaders	**Criteria for making recommendations**: Absent
		**Setting**: Clinics			
					**Summary of Findings table**: Absent
					**Certainty of Evidence rating**: Absent
					**Strength of Recommendation rating**: Absent
				**Beneficiaries**: Persons with chronic conditions	
The Royal Australian College of General Practitioners (2014)	Chronic Condition Self-management Guidelines: Summary for General Practitioners	Australia	Assist general practitioners (physicians) in facilitating self-management in patients with a chronic condition	**Users**: General practitioners (physicians)	**Criteria for making recommendations**: Absent
		**Setting**: Primary Healthcare			**Summary of Findings table**: Absent
				**Beneficiaries:** Persons with chronic conditions	
					**Certainty of Evidence rating**: NHMRC^b^ grading system
					**Strength of Recommendation rating**: NHMRC grading system
The Royal Australian College of General Practitioners (2014)	Chronic Condition Self-management Guidelines: Summary for Nurses and Allied Health Professionals	Australia	Assist nurses and allied health professionals facilitate self-management in clients with a chronic condition	**Users**: Nurses and allied health professionals	**Criteria for making recommendations**: Absent
		**Setting**: Primary Healthcare			
				**Beneficiaries**: Persons with chronic conditions	**Summary of Findings table**: Absent
					**Certainty of Evidence rating**: NHMRC grading system
					**Strength of Recommendation rating**: NHMRC grading system

^a^SIGN: Scottish Intercollegiate Guidelines Network

^b^NHMRC: National Health and Medical Research Council

### AGREE II appraisal of included guidelines

Table 2 summarises the methodological quality of the included guidelines across six domains of the AGREE II instrument. One guideline (RNAO) obtained >60% in all six domains. The median (25^th^ and 75^th^ percentile) overall assessment score in all guidelines was 2.5 (2, 3.75) out of a highest possible score of 7. None of the guidelines was *recommended* for use during the overall assessment. Only one guideline, (RNAO) [36] was *recommended with modifications*. The remaining guidelines (n = 5, NHS, RNAO, THF, RACGP1, RACGP2) were *not recommended* because of low methodological quality.

**Table 2:** AGREE II domain scores for included guidelines.

Guideline	AGREE II Domains						
	Domain 1 Scope and Purpose (%)	Domain 2 Stakeholder Involvement (%)	Domain 3 Rigour of Development (%)	Domain 4 Clarity of Presentation (%)	Domain 5 Applicability (%)	Domain 6 Editorial Independence (%)	Overall Assessment Score (1–7)
Adapting your Health Practice: Treatment and Recommendations for Unstably Housed Patients with HIV/AIDS (HCN, 2013) [34]	31	19	2	50	8	4	2
Supported self-management (NHS, 2020) [35]	22	3	1	17	4	0	2
Strategies to Support Self-Management in Chronic Conditions: Collaboration with Clients (RNAO, 2010) [36]	64	86	68	67	88	67	6
A practical guide to self-management support (THF, 2015) [37]	39	25	2	14	8	0	2
Chronic Condition Self-management Guidelines: Summary for General Practitioners (RACGP1,2014) [38]	39	19	6	25	10	0	3
Chronic Condition Self-management Guidelines: Summary for Nurses and Allied Health Professionals (RACGP2, 2014) [39].	44	22	4	28	10	17	3
**Median (Q1, Q3)**	39 (28.7, 49)	20.5 (15, 40.3)	3 (1.8, 21.5)	26.5 (16.3, 54.3)	9 (7, 29.5)	2 (0, 29.5)	2.5 (2, 3.75)

Domain Score Colours: >60% = Green, 30% - 60% = Yellow, <30% = Red

### Guideline recommendations and underlying evidence

The recommendations within the high-quality clinical practice guideline (RNAO) [36] were extracted and presented with their underlying evidence (S3 Appendix). The review focused on discussing the recommendations and underlying evidence of the high-quality guideline because it was rigorously developed and can inform future self-management support guideline adaptation.

The RNAO guideline contained 25 recommendations categorised under practice recommendations (n = 19), educational recommendations (n = 2) as well as organisation and policy recommendations (n = 4). The 19 practice recommendations were further organised under a general recommendation (n = 1), Assess (n = 5), Advise (n = 5), Agree (n = 1), Assist (n = 3), Arrange (n = 1) and Innovative Delivery Models (n = 3) (S3 Appendix). The guideline indicated the direction for each recommendation (e.g., Nurses establish rapport with clients and families), however, the strength of each recommendation (conditional or strongly recommended) was not specified.

In the absence of an EtD framework or summary of findings table in the RNAO guideline, the references discussed under each recommendation were extracted as underlying evidence (S3 Appendix). The recommendations were mainly informed by (1) systematic reviews of randomised controlled trials (RCTs) and non-RCTs, (2) results of experimental and non-experimental studies, (3) clinical guidelines as well as (4) literature reviews. The evidence informing the recommendations was produced between 1977 and 2010 and hence was considered insufficient and outdated for application in 2023. Furthermore, the underlying evidence is indirect to AWTB-HIV, who are the population of interest because it included studies among persons with chronic conditions such as diabetes mellitus, obesity, risky behaviour, tobacco addiction, asthma, hypertension, depression and HIV but not AWTB-HIV. S3 Appendix describes each recommendation and its underlying evidence.

#### Effectiveness evidence and its certainty

The underlying evidence regarding the effectiveness of self-management support was derived from systematic reviews of RCTs and non-RCTs, experimental and non-experimental studies and guidelines. The desirable effects of self-management support interventions found within the guideline were improved patient health outcomes (patient knowledge, self-management skill, quality of life, health behaviour, self-efficacy and health status). No undesirable effects of self-management support were identified. The guideline did not provide quantifiable evidence of the benefit or harms of self-management support. No summaries of findings tables were provided or referred to in the guideline.

The level/certainty of evidence ratings adopted by the RNAO for each recommendation in their guideline was an adapted SIGN grading system based on the study design of the evidence [40]. Thus, self-management support evidence derived from meta-analyses was considered the most credible evidence of effective self-management support. However, the criteria used by the guideline developers in converting evidence into recommendations were not found in the guideline. The guideline did not indicate whether the underlying evidence for each recommendation was assessed for risk of bias, inconsistency, indirectness, imprecision and publication bias. Furthermore, the guideline developers’ judgements regarding the balance of desirable and undesirable effects were not identified.

#### Economic evidence or decisions

The RNAO guideline considered the resource (human, facilities and equipment) implications of implementing self-management support and recommended adequate funding for its provision by nurses. However, the underlying evidence informing the recommendations did not include economic analysis studies examining how large the resource requirements for self-management support are or should be. The guideline did not describe the certainty of evidence on the resource requirements (costs). Only one study [42] on the cost-effectiveness of self-management support among persons with chronic conditions was identified.

#### Contextual evidence or decisions

The guideline considered contextual evidence from six qualitative studies [43–48]. No underlying evidence or judgements regarding the impact of self-management support on health equity were found in the guideline. Furthermore, the guideline did not include judgements regarding the acceptability of self-management support by stakeholders, persons with chronic conditions or AWTB-HIV and their relations. The review found one feasibility study on diabetes care management as the underlying evidence which informed the guideline’s self-management support recommendations [49].

## Discussion

This review found one high-quality best practice guideline for professional nurses to provide self-management support to persons with chronic conditions within primary health settings. Although the best practice guideline was of high methodological quality, it was underpinned by outdated and indirect evidence which may not adequately address the specific self-management support needs of AWTB-HIV, who must simultaneously self-manage TB and HIV coinfection [21, 22]. Furthermore, the high-quality best practice guideline does not report appropriate measures of effect, summaries of findings or EtD frameworks which can facilitate its adaptation. The high-quality best practice guideline could be adapted for the self-management support of AWTB-HIV if its underlying evidence is updated with current and direct evidence related to TB-HIV coinfection.

The need to develop, adopt, adapt or contextualise guidelines on self-management support for resource-limited and high-burden TB-HIV coinfection settings may be more urgent in resource-limited settings since all of the existing guidelines were developed for high-income settings such as Australia, Canada, England and the United States of America. Previous reviews have reported similar dominance of high-income countries in developing guidelines [23–26]. Guideline development groups in resource-limited settings can utilise rigorous yet pragmatic approaches such as the GRADE Adolopment approach to produce context-relevant self-management support recommendations from available high-quality guidelines [21, 50, 51].

This scoping review, however, revealed that the methodological quality of most of the existing best practice guidelines for self-management support was low. The guidelines’ low methodological quality was due to the scant transparency around the methods, evidence and judgements used in developing their recommendations. Consequently, as found in this review, the guideline (RNAO) that adequately described its scope/purpose, stakeholder involvement processes, methods and evidence used in its development, funding sources and conflict of interest declaration obtained greater than 60% in all AGREE II domains.

It was evident that all six guidelines required more rigorous and transparent processes in converting evidence to recommendations. The three guidelines (RNAO, RACGP1 and RACP2) which rated the levels of evidence informing their recommendations primarily considered the study design of the evidence but not the related factors influencing the certainty of the evidence. Thus, important factors that could reduce the guideline developers’ level of confidence in the evidence informing the recommendations (such as the risk of bias, imprecision, indirectness, inconsistency and publication bias) were not reported [52]. Furthermore, the strength and direction of the recommendation were not explicitly stated to inform stakeholders’ (clinicians, patients and policymakers) interpretation of the recommendations. Similarly, another gap found in this scoping review was that the guideline development groups did not clearly indicate the contextual judgements which informed their development of the recommendations per current internationally-accepted standards in guideline development [53]. These gaps could limit stakeholders’ application of the recommendations proposed.

The GRADE process and EtD framework provide guideline developers with a framework to transparently report their judgements regarding (1) the magnitude and certainty of evidence underlying their recommendations as well as (2) the contextual factors which informed their guideline recommendations [54]. Based on the methodological gaps identified within the guidelines apprised in this scoping review, future guideline development groups developing recommendations or guidelines should consider applying the current standards in the World Health Organization and guideline bodies to enhance the methodological quality of their guidelines. Additionally, ensuring open access to guideline development materials such as evidence profiles/syntheses and EtD framework tables can facilitate the adaptation of the recommendations [50].

### Study limitations and strengths

This scoping review appears to be the first to map out recommendations in best practice guidelines that guide professional nurses to provide self-management support to AWTB-HIV and persons with chronic conditions. It is not without limitations. For instance, only best practice guidelines in the English language were included in this review. Thus, equally relevant guidelines in other languages may have been omitted. Additionally, majority of the six guidelines found were developed in high-income countries, yet they informed most of the review’s findings and conclusions. These guidelines may need context adaptation to work in low-income countries because of social, economic and disease pattern differences between high and low-income countries. Furthermore, three guidelines were labelled as summary guidelines despite being full-text documents. Consequently, these guidelines may lack some relevant information required for their appraisal, although the reviewers considered additional information provided by the guideline authors. To limit reviewer bias, the judgements regarding guideline search, selections, data extraction, appraisal and report were conducted independently and thoroughly discussed among at least three reviewers till a consensus was achieved.

Despite these limitations, this scoping review provides systematically generated evidence regarding the scope and methodological quality of guidelines informing self-management support. The findings can inform prospective development, adaptation and review of guidelines as well as primary studies on self-management support. This review highlights the need for internationally-recognised bodies such as the World Health Organisation to develop current high-quality self-management support guidelines to serve as blueprints for adaptation into resource-limited settings.

## Conclusion

Best practice guidelines that can guide healthcare providers’ self-management support exist in the literature. However, there is a paucity of current and high-quality evidence-based recommendations that guide professional nurses to provide self-management support to AWTB-HIV. The guidelines may need thorough updating in the context of developing countries to guide healthcare delivery. The existing self-management support guidelines are not informed by current and direct evidence involving AWTB-HIV thus cannot be reliably adapted for other settings. More experimental, economic and contextual studies which examine the benefits and harms, cost-effectiveness, equity, acceptability and feasibility of self-management support among AWTB-HIV are required to inform future self-management support recommendations. Additionally, a systematic review of current effectiveness, economic and contextual studies that promote self-management among adults with chronic conditions such as TB, HIV and TB-HIV coinfection can help to produce adaptable high-quality guidelines on self-management support.

## Supporting information

S1 Checklist PRISMA-ScR checklist. (DOCX)

S1 Appendix Detailed search strategy and outputs. (DOCX)

S2 Appendix List of excluded guidelines. (DOCX)

S3 Appendix Recommendations and underlying evidence in the high-quality guideline. (DOCX)
