# Supplementary material for: Best practice guidelines for professional nurses to provide self-management support to adults with tuberculosis-human immunodeficiency virus coinfection: A scoping review
Source: PLoS One. 2023 Sep 12;18(9):e0291529. doi: 10.1371/journal.pone.0291529 (PMC10497153; doi:10.1371/journal.pone.0291529)
Supplement: S1 Appendix — (DOCX) [file pone.0291529.s002.docx]

**S1 APPENDIX**

**Detailed search strategy and outputs**

1. **DATABASES**

**Database 1**

|  | **PubMed - 13/04/2022** | **SEARCH OUTPUT** |
| --- | --- | --- |
| #1 | Search: "tuberculosis"[MeSH Terms] | 201,201 |
| #2 | Search: Tuberculosis[Title/Abstract] OR "Mycobacterium tuberculosis Infection"[Title/Abstract] OR mycobacterium[Title/Abstract] OR TB[Title/Abstract] OR "Koch’s Disease"[Title/Abstract] OR "Koch Disease"[Title/Abstract] OR "Kochs"[Title/Abstract] OR "TB HIV"[Title/Abstract] OR "HIV TB"[Title/Abstract] OR "TB/HIV"[Title/Abstract] OR "HIV/TB"[Title/Abstract] OR "TB-HIV"[Title/Abstract] OR "HIV-TB"[Title/Abstract] OR "HIV-TB coinfection"[Title/Abstract] OR "HIV-TB co-infection"[Title/Abstract] OR "HIV-associated TB"[Title/Abstract] OR "HIV/TB co-infection"[Title/Abstract] OR Coinfections[Title/Abstract] OR "Co-infections"[Title/Abstract] OR "Co infection"[Title/Abstract] OR "comorbidity"[Title/Abstract] OR "co morbidity"[Title/Abstract] OR "mixed infections"[Title/Abstract] OR "Polymicrobial Infection"[Title/Abstract] OR "Secondary Infections"[Title/Abstract] OR "chronic condition"[Title/Abstract] OR "Chronic illness"[Title/Abstract] OR "long term illness"[Title/Abstract] OR "long term condition"[Title/Abstract] OR "long-term condition"[Title/Abstract] | 402,485 |
| #3 | #1 OR #2 | 435,695 |
| #4 | HIV[MeSH Terms] | 104,937 |
| #5 | "Human immunodeficiency virus"[Title/Abstract] OR HIV[Title/Abstract] OR "Human T Cell Lymphotropic Virus Type III"[Title/Abstract] OR "Human T-Cell Lymphotropic Virus Type III"[Title/Abstract] OR "Human T-Cell Leukemia Virus Type III"[Title/Abstract] OR "LAV-HTLV-III"[Title/Abstract] OR "Lymphadenopathy-Associated Virus"[Title/Abstract] OR "Lymphadenopathy Associated Virus"[Title/Abstract] OR "Human T Lymphotropic Virus Type III"[Title/Abstract] OR "Human T-Lymphotropic Virus Type III"[Title/Abstract] OR "AIDS Virus"[Title/Abstract] OR "Acquired Immune Deficiency Syndrome Virus"[Title/Abstract] OR "Acquired Immunodeficiency Syndrome Virus"[Title/Abstract] OR "HTLV-III"[Title/Abstract] | 359,477 |
| #6 | HIV infections[MeSH Terms] | 303,768 |
| #7 | "HIV infections"[Title/Abstract] OR "HTLV-III-LAV Infections"[Title/Abstract] OR "HTLV III LAV Infections"[Title/Abstract] OR "HTLV-III-LAV Infection"[Title/Abstract] OR "HTLV-III Infections"[Title/Abstract] OR "HTLV III Infections"[Title/Abstract] OR "HTLV-III Infection"[Title/Abstract] OR "T-Lymphotropic Virus Type III Infections"[Title/Abstract] OR "HIV Coinfection"[Title/Abstract] | 13,677 |
| #8 | #4 OR #5 OR #6 OR #7 | 430,553 |
| #9 | Self-management[MeSH Terms] | 4,398 |
| #10 | "self-management"[Title/Abstract] OR "self management"[Title/Abstract] OR "self-manage"[Title/Abstract] OR "self manage"[Title/Abstract] OR "self-management support"[Title/Abstract] OR "self management support"[Title/Abstract] OR support[Title/Abstract] OR "self-care"[Title/Abstract] OR "self care"[Title/Abstract] OR "self-care support"[Title/Abstract] OR "self care support"[Title/Abstract] OR "goal setting"[Title/Abstract] OR "goal-setting"[Title/Abstract] OR "follow-up"[Title/Abstract] OR "follow up"[Title/Abstract] OR activation[Title/Abstract] OR activate[Title/Abstract] OR collaborat*[Title/Abstract] OR adherence[Title/Abstract] OR adher*[Title/Abstract] OR compliance[Title/Abstract] OR concordance[Title/Abstract] OR "drop out"[Title/Abstract] OR "drop-out"[Title/Abstract] OR "treatment refusal"[Title/Abstract] OR "self-efficacy"[Title/Abstract] OR "self efficacy"[Title/Abstract] OR efficacy[Title/Abstract] OR confidence[Title/Abstract] OR "self-confidence"[Title/Abstract] OR "self confidence"[Title/Abstract] OR motivation[Title/Abstract] OR "social support"[Title/Abstract] OR assistance[Title/Abstract] OR help[Title/Abstract] | 5,485,334 |
| #11 | #9 OR #10 | 5,485,787 |
| #12 | Primary Health Care[MeSH Terms] | 181,481 |
| #13 | "Primary Health Care"[Title/Abstract] OR "primary health"[Title/Abstract] OR "healthcare setting"[Title/Abstract] OR "healthcare"[Title/Abstract] OR clinic[Title/Abstract] | 607,794 |
| #14 | #12 OR #13 | 749,764 |
| #15 | "Clinical protocols"[MESH] OR "Consensus"[MESH] OR "Consensus development conferences as topic"[MESH] OR "Critical pathways"[MESH] OR "Guidelines as topic" OR "Practice guidelines as topic"[MESH] OR "Health planning guidelines"[MESH] OR "Clinical Decision Rules"[MESH] | 377,894 |
| #16 | guideline[Title/Abstract] OR "practice guideline"[Title/Abstract] OR "consensus development conference"[Title/Abstract] OR "position statement*"[Title/Abstract] OR "policy statement*"[Title/Abstract] OR "practice parameter*"[Title/Abstract] OR "best practice*"[Title/Abstract] OR standards[Title/Abstract] OR guidelines[Title/Abstract] OR guideline*[Title/Abstract] OR consensus*[Title/Abstract] OR recommendat*[Title/Abstract] OR "treatment guideline*"[Title/Abstract] OR CPG[Title/Abstract] OR CPGs[Title/Abstract] OR "clinical guideline*"[Title/Abstract] OR "guideline recommendation*"[Title/Abstract] OR practice[Title/Abstract] OR pathways[Title/Abstract] OR protocol[Title/Abstract] OR bulletins[Title/Abstract] OR OR[Title/Abstract] | 11,264,777 |
| #17 | #15 OR #16 | 11,400,202 |
| #18 | nurse[MeSH Terms] | 95,087 |
| #19 | nurse[Title/Abstract] OR nurs*[Title/Abstract] | 503,689 |
| #20 | #18 OR #19 | 537,862 |
| #21 | #3 AND #8 AND #11 AND #14 AND #17 | 936 |
| #22 | #20 AND #21 | 72 |

**Database 2**

|  | **Cumulative Index for Nursing and Allied Health Literature (CINAHL) (25/05/2022)** | **SEARCH OUTPUT** |
| --- | --- | --- |
| S1 | Tuberculosis OR “Mycobacterium tuberculosis Infection” OR mycobacterium OR TB OR “Koch’s Disease” OR “Koch Disease” OR “Kochs” OR “TB HIV” OR “HIV TB” OR “TB/HIV” OR “HIV/TB” OR “TB-HIV” OR “HIV-TB” OR “HIV-TB coinfection” OR “HIV-TB co-infection” OR “HIV-associated TB” OR “HIV/TB co-infection” OR Coinfections OR “Co-infections” OR “Co infection” OR “comorbidity” OR “co morbidity” OR “mixed infections” OR “Polymicrobial Infection” OR “Secondary Infections” OR “chronic condition” OR “Chronic illness” OR “long term illness” OR “long term condition” OR “long-term condition” | 157,491 |
| S2 | “Human immunodeficiency virus” OR HIV OR “Human T Cell Lymphotropic Virus Type III” OR “Human T-Cell Lymphotropic Virus Type III” OR “Human T-Cell Leukemia Virus Type III” OR “LAV-HTLV-III” OR “Lymphadenopathy-Associated Virus” OR “Lymphadenopathy Associated Virus” OR “Human T Lymphotropic Virus Type III” OR “Human T-Lymphotropic Virus Type III” OR “AIDS Virus” OR “Acquired Immune Deficiency Syndrome Virus” OR “Acquired Immunodeficiency Syndrome Virus” OR “HTLV-III” OR “HIV infections” OR “HTLV-III-LAV Infections” OR “HTLV III LAV Infections” OR “HTLV-III-LAV Infection” OR “HTLV-III Infections” OR “HTLV III Infections” OR “HTLV-III Infection” OR “T-Lymphotropic Virus Type III Infections” OR “HIV Coinfection” | 125,076 |
| S3 | S1 OR S2 | 270,462 |
| S4 | “self-management” OR “self management” OR “self-manage” OR “self manage” OR “self-management support” OR “self management support” OR support OR “self-care” OR “self care” OR “self-care support” OR “self care support” OR “goal setting” OR “goal-setting” OR “follow-up” OR “follow up” OR activation OR activate OR collaborat* OR adherence OR adher* OR compliance OR concordance OR “drop out” OR “drop-out” OR “treatment refusal” OR “self-efficacy” OR “self efficacy” OR efficacy OR confidence OR “self-confidence” OR “self confidence” OR motivation OR “social support” OR assistance OR help | 1,802,927 |
| S5 | “primary health care” OR “primary health” OR “healthcare setting” OR “healthcare” OR clinic | 388,817 |
| S6 | guideline OR "practice guideline" OR "consensus development conference" OR "consensus development conference" OR "position statement*" OR "policy statement*" OR "practice parameter*" OR "best practice*" OR standards OR guideline OR guidelines OR guideline* OR consensus* OR recommendat* OR "treatment guideline*" OR CPG OR CPGs OR "clinical guideline*" OR "guideline recommendation*" OR practice OR pathways OR protocol OR bulletins | 1,463,532 |
| S7 | S3 AND S4 AND S5 AND S6 | 5,354 |
| S8 | nurse OR nurs* | 943,414 |
| S9 | S7 AND S8 | 1,014 |
| S10 | S7 AND S8 – Filter: Date: 01/01/2010 to 25/05/2022,  English language. | 793 |

**Database 3**

|  | **AFRICA-WIDE INFORMATION (25/05/2022)** | **SEARCH OUTPUT** |
| --- | --- | --- |
| S1 | Tuberculosis OR “Mycobacterium tuberculosis Infection” OR mycobacterium OR TB OR “Koch’s Disease” OR “Koch Disease” OR “Kochs” OR “TB HIV” OR “HIV TB” OR “TB/HIV” OR “HIV/TB” OR “TB-HIV” OR “HIV-TB” OR “HIV-TB coinfection” OR “HIV-TB co-infection” OR “HIV-associated TB” OR “HIV/TB co-infection” OR Coinfections OR “Co-infections” OR “Co infection” OR “comorbidity” OR “co morbidity” OR “mixed infections” OR “Polymicrobial Infection” OR “Secondary Infections” OR “chronic condition” OR “Chronic illness” OR “long term illness” OR “long term condition” OR “long-term condition” | 225,272 |
| S2 | “Human immunodeficiency virus” OR HIV OR “Human T Cell Lymphotropic Virus Type III” OR “Human T-Cell Lymphotropic Virus Type III” OR “Human T-Cell Leukemia Virus Type III” OR “LAV-HTLV-III” OR “Lymphadenopathy-Associated Virus” OR “Lymphadenopathy Associated Virus” OR “Human T Lymphotropic Virus Type III” OR “Human T-Lymphotropic Virus Type III” OR “AIDS Virus” OR “Acquired Immune Deficiency Syndrome Virus” OR “Acquired Immunodeficiency Syndrome Virus” OR “HTLV-III” OR “HIV infections” OR “HTLV-III-LAV Infections” OR “HTLV III LAV Infections” OR “HTLV-III-LAV Infection” OR “HTLV-III Infections” OR “HTLV III Infections” OR “HTLV-III Infection” OR “T-Lymphotropic Virus Type III Infections” OR “HIV Coinfection” | 122,324 |
| S3 | S1 OR S2 | 322,604 |
| S4 | “self-management” OR “self management” OR “self-manage” OR “self manage” OR “self-management support” OR “self management support” OR support OR “self-care” OR “self care” OR “self-care support” OR “self care support” OR “goal setting” OR “goal-setting” OR “follow-up” OR “follow up” OR activation OR activate OR collaborat* OR adherence OR adher* OR compliance OR concordance OR “drop out” OR “drop-out” OR “treatment refusal” OR “self-efficacy” OR “self efficacy” OR efficacy OR confidence OR “self-confidence” OR “self confidence” OR motivation OR “social support” OR assistance OR help | 405,277 |
| S5 | “primary health care” OR “primary health” OR “healthcare setting” OR “healthcare” OR clinic | 84,166 |
| S6 | guideline OR "practice guideline" OR "consensus development conference" OR "consensus development conference" OR "position statement*" OR "policy statement*" OR "practice parameter*" OR "best practice*" OR standards OR guideline OR guidelines OR guideline* OR consensus* OR recommendat* OR "treatment guideline*" OR CPG OR CPGs OR "clinical guideline*" OR "guideline recommendation*" OR practice OR pathways OR protocol OR bulletins | 457,323 |
| S7 | S3 AND S4 AND S5 AND S6 | 3,297 |
| S8 | nurse OR nurs* | 46,998 |
| S9 | S7 AND S8 | 616 |
| S10 | S7 AND S8, Filter: Date: 01/01/2010 to 25/05/2022,  English language. | 329 |

**Database 4**

|  | **SCOPUS (16/04/2022)** | **SEARCH OUTPUT** |
| --- | --- | --- |
| #1 | TITLE-ABS ( tuberculosis  OR  "Mycobacterium tuberculosis Infection"  OR  mycobacterium  OR  tb  OR  "Koch's Disease"  OR  "Koch Disease"  OR  "Kochs"  OR  "TB HIV"  OR  "HIV TB"  OR  "TB/HIV"  OR  "HIV/TB"  OR  "TB-HIV"  OR  "HIV-TB"  OR  "HIV-TB coinfection"  OR  "HIV-TB co-infection"  OR  "HIV-associated TB"  OR  "HIV/TB co-infection"  OR  coinfections  OR  "Co-infections"  OR  "Co infection"  OR  "comorbidity"  OR  "co morbidity"  OR  "mixed infections"  OR  "Polymicrobial Infection"  OR  "Secondary Infections"  OR  "chronic condition"  OR  "Chronic illness"  OR  "long term illness"  OR  "long term condition"  OR  "long-term condition" ) | 522,995 |
| #2 | TITLE-ABS ( "Human immunodeficiency virus"  OR  hiv  OR  "Human T Cell Lymphotropic Virus Type III"  OR  "Human T-Cell Lymphotropic Virus Type III"  OR  "Human T-Cell Leukemia Virus Type III"  OR  "LAV-HTLV-III"  OR  "Lymphadenopathy-Associated Virus"  OR  "Lymphadenopathy Associated Virus"  OR  "Human T Lymphotropic Virus Type III"  OR  "Human T-Lymphotropic Virus Type III"  OR  "AIDS Virus"  OR  "Acquired Immune Deficiency Syndrome Virus"  OR  "Acquired Immunodeficiency Syndrome Virus"  OR  "HTLV-III"  OR  "HIV infections"  OR  "HTLV-III-LAV Infections"  OR  "HTLV III LAV Infections"  OR  "HTLV-III-LAV Infection"  OR  "HTLV-III Infections"  OR  "HTLV III Infections"  OR  "HTLV-III Infection"  OR  "T-Lymphotropic Virus Type III Infections"  OR  "HIV Coinfection" ) | 411,258 |
| #3 | TITLE-ABS ( tuberculosis  OR  "Mycobacterium tuberculosis Infection"  OR  mycobacterium  OR  tb  OR  "Koch's Disease"  OR  "Koch Disease"  OR  "Kochs"  OR  "TB HIV"  OR  "HIV TB"  OR  "TB/HIV"  OR  "HIV/TB"  OR  "TB-HIV"  OR  "HIV-TB"  OR  "HIV-TB coinfection"  OR  "HIV-TB co-infection"  OR  "HIV-associated TB"  OR  "HIV/TB co-infection"  OR  coinfections  OR  "Co-infections"  OR  "Co infection"  OR  "comorbidity"  OR  "co morbidity"  OR  "mixed infections"  OR  "Polymicrobial Infection"  OR  "Secondary Infections"  OR  "chronic condition"  OR  "Chronic illness"  OR  "long term illness"  OR  "long term condition"  OR  "long-term condition"  OR  "Human immunodeficiency virus"  OR  hiv  OR  "Human T Cell Lymphotropic Virus Type III"  OR  "Human T-Cell Lymphotropic Virus Type III"  OR  "Human T-Cell Leukemia Virus Type III"  OR  "LAV-HTLV-III"  OR  "Lymphadenopathy-Associated Virus"  OR  "Lymphadenopathy Associated Virus"  OR  "Human T Lymphotropic Virus Type III"  OR  "Human T-Lymphotropic Virus Type III"  OR  "AIDS Virus"  OR  "Acquired Immune Deficiency Syndrome Virus"  OR  "Acquired Immunodeficiency Syndrome Virus"  OR  "HTLV-III"  OR  "HIV infections"  OR  "HTLV-III-LAV Infections"  OR  "HTLV III LAV Infections"  OR  "HTLV-III-LAV Infection"  OR  "HTLV-III Infections"  OR  "HTLV III Infections"  OR  "HTLV-III Infection"  OR  "T-Lymphotropic Virus Type III Infections"  OR  "HIV Coinfection" ) | 899,412 |
| #4 | TITLE-ABS ( "self-management"  OR  "self management"  OR  "self-manage"  OR  "self manage"  OR  "self-management support"  OR  "self management support"  OR  support  OR  "self-care"  OR  "self care"  OR  "self-care support"  OR  "self care support"  OR  "goal setting"  OR  "goal-setting"  OR  "follow-up"  OR  "follow up"  OR  activation  OR  activate  OR  collaborat*  OR  adherence  OR  adher*  OR  compliance  OR  concordance  OR  "drop out"  OR  "drop-out"  OR  "treatment refusal"  OR  "self-efficacy"  OR  "self efficacy"  OR  efficacy  OR  confidence  OR  "self-confidence"  OR  "self confidence"  OR  motivation  OR  "social support"  OR  assistance  OR  help ) | 10,654,605 |
| #5 | TITLE-ABS ( "primary health care"  OR  "primary health"  OR  "healthcare setting"  OR  "healthcare"  OR  clinic ) | 883, 129 |
| #6 | TITLE-ABS ( guideline  OR  "practice guideline"  OR  "consensus development conference"  OR  "consensus development conference"  OR  "position statement*"  OR  "policy statement*"  OR  "practice parameter*"  OR  "best practice*"  OR  standards  OR  guideline  OR  guidelines  OR  guideline*  OR  consensus*  OR  recommendat*  OR  "treatment guideline*"  OR  cpg  OR  cpgs  OR  "clinical guideline*"  OR  "guideline recommendation*"  OR  practice  OR  pathways  OR  protocol  OR  bulletins ) | 8,840,319 |
| #7 | #1 AND #2 AND #3 AND #4 AND #5 AND #6 | 7,670 |
| #8 | TITLE-ABS ( nurse  OR  nurs* ) | 615,226 |
| #9 | #7 AND #8 | 929 |
| #10 | #9, Filter: Date: 01/01/2010 to 25/05/2022  English language. | 716 |

**Database 5**

|  | **WEB OF SCIENCE (25/05/2022)** | **SEARCH OUTPUT** |
| --- | --- | --- |
| #1 | Tuberculosis OR “Mycobacterium tuberculosis Infection” OR mycobacterium OR TB OR “Koch’s Disease” OR “Koch Disease” OR “Kochs” OR “TB HIV” OR “HIV TB” OR “TB/HIV” OR “HIV/TB” OR “TB-HIV” OR “HIV-TB” OR “HIV-TB coinfection” OR “HIV-TB co-infection” OR “HIV-associated TB” OR “HIV/TB co-infection” OR Coinfections OR “Co-infections” OR “Co infection” OR “comorbidity” OR “co morbidity” OR “mixed infections” OR “Polymicrobial Infection” OR “Secondary Infections” OR “chronic condition” OR “Chronic illness” OR “long term illness” OR “long term condition” OR “long-term condition” (Topic) | 407,046 |
| #2 | TS=(“Human immunodeficiency virus” OR HIV OR “Human T Cell Lymphotropic Virus Type III” OR “Human T-Cell Lymphotropic Virus Type III” OR “Human T-Cell Leukemia Virus Type III” OR “LAV-HTLV-III” OR “Lymphadenopathy-Associated Virus” OR “Lymphadenopathy Associated Virus” OR “Human T Lymphotropic Virus Type III” OR “Human T-Lymphotropic Virus Type III” OR “AIDS Virus” OR “Acquired Immune Deficiency Syndrome Virus” OR “Acquired Immunodeficiency Syndrome Virus” OR “HTLV-III” OR “HIV infections” OR “HTLV-III-LAV Infections” OR “HTLV III LAV Infections” OR “HTLV-III-LAV Infection” OR “HTLV-III Infections” OR “HTLV III Infections” OR “HTLV-III Infection” OR “T-Lymphotropic Virus Type III Infections” OR “HIV Coinfection”) | 433,802 |
| #3 | #1 OR #2 | 807,175 |
| #4 | TS=(“self-management” OR “self management” OR “self-manage” OR “self manage” OR “self-management support” OR “self management support” OR support OR “self-care” OR “self care” OR “self-care support” OR “self care support” OR “goal setting” OR “goal-setting” OR “follow-up” OR “follow up” OR activation OR activate OR collaborat* OR adherence OR adher* OR compliance OR concordance OR “drop out” OR “drop-out” OR “treatment refusal” OR “self-efficacy” OR “self efficacy” OR efficacy OR confidence OR “self-confidence” OR “self confidence” OR motivation OR “social support” OR assistance OR help) | 10,336,298 |
| #5 | TS=(“primary health care” OR “primary health” OR “healthcare setting” OR “healthcare” OR clinic) | 641,808 |
| #6 | TS=(guideline OR "practice guideline" OR "consensus development conference" OR "consensus development conference" OR "position statement*" OR "policy statement*" OR "practice parameter*" OR "best practice*" OR standards OR guideline OR guidelines OR guideline* OR consensus* OR recommendat* OR "treatment guideline*" OR CPG OR CPGs OR "clinical guideline*" OR "guideline recommendation*" OR practice OR pathways OR protocol OR bulletins ) | 7,086,338 |
| #7 | #3 AND #4 AND #5 AND #6 | 7,900 |
| #8 | TS=(nurse OR nurs*) | 366,684 |
| #9 | #7 AND #8 | 880 |
| #10 | #7 AND #8 - Filter: Date: 01/01/2010 to 25/05/2022,  English language. | 708 |

**Database 6**

|  | **TRIP DATABASE (19/04/2022)** | **SEARCH OUTPUT** |
| --- | --- | --- |
| #1 | Tuberculosis | 630 |
| #2 | HIV | 1,536 |
| #3 | TB HIV | 1,779 |
| #4 | self-management | 734 |
| #5 | TB HIV self-management | 45 |
| #6 | TB HIV self-management guideline | 44 |
| #7 | #1 - #6 - Filter: Date: 01/01/2010 to 19/04/2022, English language. | 89 |

| **TOTAL NUMBER OF RECORDS FROM SIX (6) DATABASES:** 2,707 RECORDS |
| --- |

**2. GREY LITERATURE SEARCH**

The results of grey literature search conducted in Guideline Clearing Houses/ Organizations and Search Engines are reported below.

**2.1 GUIDELINE CLEARING HOUSES/ORGANIZATIONS**

**Guideline Clearing House/Organization 1**

|  | **NATIONAL INSTITUTE OF CLINICAL EXCELLENCE (NICE) (25/04/2022)** | **SEARCH OUTPUT** |
| --- | --- | --- |
| #1 | Tuberculosis | 67 |
| #2 | HIV | 115 |
| #3 | TB HIV | 20 |
| #4 | self-management | 145 |
| #5 | TB HIV self-management | 12 |
| #6 | TB self-manage | 14 |
| #7 | HIV self-manage | 16 |
| #8 | #1 - #7 Filter: Date: 01/01/2010 to 25/04/2022, English language.  First 5 pages (10 per page) | 37 |

**Guideline Clearing House/Organization 2**

|  | **GUIDELINES INTERNATIONAL NETWORK (GIN) (25/04/2022)** | **SEARCH OUTPUT** |
| --- | --- | --- |
| #1 | Tuberculosis | 9 |
| #2 | HIV | 13 |
| #3 | TB HIV | 0 |
| #4 | self management | 11 |
| #5 | TB HIV self management | 0 |
| #6 | TB self manage | 0 |
| #7 | HIV self manage | 0 |
| #8 | #1 - #7 Filter: Date: 01/01/2010 to 25/04/2022, English language  First 2 pages (10 per page) | 7 |

**Guideline Clearing House/Organization 3**

|  | **SCOTTISH INTERCOLLEGIATE GUIDELINES NETWORK (SIGN) (26/04/2022)** | **SEARCH OUTPUT** |
| --- | --- | --- |
| #1 | Tuberculosis | 0 |
| #2 | HIV | 0 |
| #3 | TB HIV | 0 |
| #4 | self management | 90 |
| #5 | TB HIV self management | 92 |
| #6 | TB self manage | 92 |
| #7 | HIV self manage | 81 |
| #8 | #1 - #7 Filter: Date: 01/01/2010 to 26/04/2022, English language.  First 5 pages (10 per page) | 1 |

**Guideline Clearing House/Organization 4**

|  | **CANADIAN MEDICAL ASSOCIATION INFOBASE (CMA) (26/04/2022)** | **SEARCH OUTPUT** |
| --- | --- | --- |
| #1 | Tuberculosis | 15 |
| #2 | HIV | 33 |
| #3 | TB HIV | 0 |
| #4 | self management | 3 |
| #5 | TB HIV self management | 0 |
| #6 | TB self manage | 0 |
| #7 | HIV self manage | 0 |
| #8 | #1 - #7 Filter: Date: 01/01/2010 to 26/04/2022, English language.  First 5 pages (10 per page) | 18 |

**Guideline Clearing House/Organization 5**

|  | **GUIDELINE.GOV (AGENCY FOR HEALTHCARE RESEARCH AND QUALITY) (26/04/2022)** | **SEARCH OUTPUT** |
| --- | --- | --- |
| #1 | Tuberculosis | 650 |
| #2 | HIV | 2,538 |
| #3 | TB HIV | 158 |
| #4 | self management | 7,658 |
| #5 | TB HIV self management | 94 |
| #6 | TB self manage | 225 |
| #7 | HIV self manage | 890 |
| #8 | #1 - #7 Filter: Date: 01/01/2010 to 26/04/2022, English language.  First 5 pages (10 per page) | 19 |

**Guideline Clearing House/Organization 6**

|  | **NEW ZEALAND GUIDELINE GROUP (27/04/2022)** | **SEARCH OUTPUT** |
| --- | --- | --- |
| #1 | Tuberculosis | 7 |
| #2 | HIV | 5 |
| #3 | TB HIV | 2 |
| #4 | self management | 22 |
| #5 | TB HIV self management | 2 |
| #6 | TB self manage | 26 |
| #7 | HIV self manage | 23 |
| #8 | #1 - #7 Filter: Date: 01/01/2010 to 27/04/2022, English language.  First 5 pages (10 per page) | 13 |

**Guideline Clearing House/Organization 7**

|  | **REGISTERED NURSES’ ASSOCIATION OF ONTARIO (28/04/2022)** | **SEARCH OUTPUT** |
| --- | --- | --- |
| #1 | Tuberculosis | 0 |
| #2 | HIV | 2 |
| #3 | TB HIV | 2 |
| #4 | self management | 4 |
| #5 | TB HIV self management | 0 |
| #6 | TB self manage | 0 |
| #7 | HIV self manage | 6 |
| #8 | #1 - #7 Filter: Date: 01/01/2010 to 27/04/2022, English language.  First 5 pages (10 per page) | 6 |

**Guideline Clearing House/Organization 8**

|  | **GHANA AIDS COMMISSION (06/05/2022)** | **SEARCH OUTPUT** |
| --- | --- | --- |
| #1 | Tuberculosis | 10 |
| #2 | HIV | 10 |
| #3 | TB HIV | 10 |
| #4 | self-management | 10 |
| #5 | TB HIV self management | 10 |
| #6 | #1 - #5 Filter: Date: 01/01/2010 to 06/05/2022, English language.  First 5 pages (10 per page) | 6 |

**Guideline Clearing House/Organization 9**

|  | **GHANA NATIONAL TUBERCULOSIS CONTROL PROGRAMME (06/05/2022)** | **SEARCH OUTPUT** |
| --- | --- | --- |
| #1 | Tuberculosis | 0 |
| #2 | HIV | 0 |
| #3 | TB HIV | 0 |
| #4 | self-management | 0 |
| #5 | TB HIV self management | 0 |
| #6 | #1 - #5 Filter: Date: 01/01/2010 to 06/05/2022, English language.  First 5 pages (10 per page) | 0 |

**Guideline Clearing House/Organization 10**

|  | **GHANA MINISTRY OF HEALTH (GHANA HEALTH SERVICE) (06/05/2022)** | **SEARCH OUTPUT** |
| --- | --- | --- |
| #1 | Tuberculosis | 1 |
| #2 | HIV | 1 |
| #3 | TB HIV | 1 |
| #4 | self-management | 0 |
| #5 | TB HIV self management | 0 |
| #6 | #1 - #5 Filter: Date: 01/01/2010 to 06/05/2022, English language.  First 5 pages (10 per page) | 2 |

**Guideline Clearing House/Organization 11**

|  | **WORLD HEALTH ORGANIZATION (who.int) (06/05/2022)** | **SEARCH OUTPUT** |
| --- | --- | --- |
| #1 | Tuberculosis | 26,720 |
| #2 | HIV | 24,998 |
| #3 | TB HIV | 9,082 |
| #4 | self-management | 19,565 |
| #5 | TB HIV self-management | 15,042 |
| #6 | TB HIV self-management guideline | 12267 |
| #7 | #1 - #6 - Filter: Date: 01/01/2010 to 06/05/2022, English language.  First 5 pages (10 per page) | 51 |

**Guideline Clearing House/Organization 12**

|  | **MEDBOX (06/05/2022)** | **SEARCH OUTPUT** |
| --- | --- | --- |
| #1 | Tuberculosis | 200 |
| #2 | HIV | 200 |
| #3 | TB HIV | 200 |
| #4 | self-management | 200 |
| #5 | TB HIV self management | 200 |
| #6 | TB self manage | 200 |
| #7 | HIV self manage | 200 |
| #8 | #1 - #7 - Filter: Date: 01/01/2010 to 29/04/2022, English language.  First 5 pages (10 per page) | 40 |

| **TOTAL NUMBER OF RECORDS FROM GUIDELINE CLEARING HOUSES /ORGANIZATIONS :** 200 RECORDS |
| --- |

**2.2 WEBSITES/SEARCH ENGINES**

**Websites/search engine 1**

|  | **RESEARCH GATE (29/04/2022)** | **SEARCH OUTPUT** |
| --- | --- | --- |
| #1 | TB HIV self management guideline | 22 |
| #2 | TB HIV self management guideline | 25 |
| #3 | #1 - #2 - Filter: Date: 01/01/2010 to 29/04/2022, English language.  First 10 pages (10 per page) | 5 |

**Websites/search engine 2**

|  | **GOOGLE (06/05/2022)** | **SEARCH OUTPUT** |
| --- | --- | --- |
| #1 | Tuberculosis | 2,070,000,000 |
| #2 | HIV | 3,650,000,000 |
| #3 | TB HIV | 205,000,000 |
| #4 | self-management | 4,000,000,000 |
| #5 | self-management guideline | 7,300,000,000 |
| #6 | TB HIV self management | 24,000,000 |
| #7 | TB HIV self management guideline | 13,800,000 |
| #8 | #1 - #7 - Filter: Date: 01/01/2010 to 06/05/2022, English language.  First 10 pages (10 per page) | 53 |

**Websites/search engine 3**

|  | **GOOGLE SCHOLAR (19/04/2022)** | **SEARCH OUTPUT** |
| --- | --- | --- |
| #1 | Tuberculosis | 3,260,000 |
| #2 | HIV | 3,260,000 |
| #3 | TB HIV | 1,290,000 |
| #4 | self-management | 1,650,000 |
| #5 | TB HIV self-management | 13,200 |
| #6 | TB HIV self-management guideline | 17,800 |
| #7 | #1 - #6 - Filter: Date: 01/01/2010 to 19/04/2022, English language.  First 10 pages (10 per page) | 3 |

| **TOTAL NUMBER OF RECORDS FROM WEBSITES/SEARCH ENGINES:** 61 RECORDS |
| --- |
