# Supplementary material for: Best practice guidelines for professional nurses to provide self-management support to adults with tuberculosis-human immunodeficiency virus coinfection: A scoping review
Source: PLoS One. 2023 Sep 12;18(9):e0291529. doi: 10.1371/journal.pone.0291529 (PMC10497153; doi:10.1371/journal.pone.0291529)
Supplement: S2 Appendix — (DOCX) [file pone.0291529.s003.docx]

**S2 APPENDIX**

**List of guidelines excluded during full-text screening**

| **No.** | **Document** | **Reason for exclusion** |
| --- | --- | --- |
| 1 | 360 F. Nursing Care of Patients with HIV-AIDS - Facilitator’s Guide Presentation. | Duplicate study |
| 2 | AHRQ Health Information Technology Research. 2018; Available from: https://drive.google.com/file/d/1YgXRR5AVGtArYkrVQZ6f36jLCDbWj3qK/view?usp=sharing | Ineligible study design (Not a guideline) |
| 3 | AHRQ. External Resources on Self-Management Support. | Ineligible population (Adults with TB-HIV, TB, HIV, Chronic condition) |
| 4 | AIDSinfo. Guidance for Non-HIV-Specialized Providers Caring for Persons with HIV Displaced by Disasters Guidance for Non-HIV-Specialized Providers Caring for Persons with HIV Who Have Been Displaced by Disasters ( such as a Hurricane ) Essential Information for Man. 2020; | Ineligible intervention/phenomena of interest (Not self-management support) |
| 5 | Adams JA, Whiteman K, McGraw S. Reducing Missed Appointments for Patients with HIV: An Evidence-Based Approach. Journal of Nursing Care Quality [Internet]. 2020;35(2):170. Available from: https://search.ebscohost.com/login.aspx?direct=true&db=cin20&AN=142036651&site=ehost-live&scope=site | Ineligible study design (Not a guideline) |
| 6 | Agency for Healthcare Research and Quality Advancing Excellence in Health Care. Patient Self ­ Management Support Patient Self-Management Support: A Chronic Conditions Practice Improvement Activity User Guide. | Duplicate study |
| 7 | Ahmed AI, Soliman SM, Awad LA. Validation of evidence-based clinical practice guideline : Nursing intervention for newly diagnosed pulmonary tuberculosis patients at community setting. Alexandria Journal of Medicine [Internet]. 2012;48(2):165. Available from: http://dx.doi.org/10.1016/j.ajme.2011.08.002 | Ineligible study design (Not a guideline) |
| 8 | Alvarez GG, Pease C, Menzies D. Chapter 6 : Tuberculosis preventive treatment in adults. Canadian Journal of Respiratory, Critical Care, and Sleep Medicine [Internet]. 2022;6(S1):86. Available from: https://doi.org/10.1080/24745332.2022.2039498 | Ineligible intervention/phenomena of interest (Not self-management support) |
| 9 | An update on tuberculosis. Practice Nurse [Internet]. 2020;50(7):40. Available from: https://search.ebscohost.com/login.aspx?direct=true&db=cin20&AN=145483873&site=ehost-live&scope=site | Ineligible intervention/phenomena of interest (Not self-management support) |
| 10 | Anokye K, Hospital T, Sciences AH. Guidelines for antiretroviral therapy in Ghana 1. 2015;(November). | Ineligible intervention/phenomena of interest (Not self-management support) |
| 11 | Anon A. The Joint WHO-ILO-UNAIDS policy guidelines : improving health workers’ access to HIV and TB prevention, treatment, care and support services. HIV Nursing Matters [A Magazine of the Southern African HIV Clinicians Society] [Internet]. 2012 Jan;3(1):40. Available from: https://search.ebscohost.com/login.aspx?direct=true&db=awn&AN=902229&site=ehost-live&scope=site | Ineligible intervention/phenomena of interest (Not self-management support) |
| 12 | Approach PH, On CG. Guidelines hiv prevention, testing, treatment, service delivery and monitoring: 2021;(July). | Duplicate study |
| 13 | Baboolal S, American T, For S, Rastogi N. Caribbean Guidelines for the Prevention , Treatment , Care , and Control of Tuberculosis and TB / HIV. 2010;(April). | Ineligible intervention/phenomena of interest (Not self-management support) |
| 14 | Baker DC. Routine Inpatient Human Immunodeficiency Virus Screening: Missed Prevention Opportunities. Clinical Nurse Specialist: The Journal for Advanced Nursing Practice [Internet]. 2017;31(1):51. Available from: https://search.ebscohost.com/login.aspx?direct=true&db=cin20&AN=120544419&site=ehost-live&scope=site | Ineligible study design (Not a guideline) |
| 15 | Barbour A, Coyne K, Freedman A, Jelliman P, Khoo S, Seden K, et al. British HIV Association guidelines for the management of TB/HIV co-infection in adults 2017. 2017;29, 35, 63. | Ineligible intervention/phenomena of interest (Not self-management support) |
| 16 | Battersby M, Von Korff M, Schaefer J, Davis C, Ludman E, Greene SM, et al. Twelve evidence-based principles for implementing self-management support in primary care. Joint Commission Journal on Quality and Patient Safety [Internet]. 2010;36(12):570. Available from: https://search.ebscohost.com/login.aspx?direct=true&db=cin20&AN=104974544&site=ehost-live&scope=site | Ineligible study design (Not a guideline) |
| 17 | Behaviour change : individual approaches. 2022;(January 2014). | Ineligible intervention/phenomena of interest (Not self-management support) |
| 18 | Bodenheimer TA. Helping Patients Help Themselves: How to Implement Self-Management Support. 2010; | Ineligible study design (Not a guideline) |
| 19 | Bogardus MA. Best Practices and Self-Care to Support Women in Living Well with Human Immunodeficiency Virus/AIDS. Nursing Clinics of North America [Internet]. 2018;53(1):82. Available from: https://search.ebscohost.com/login.aspx?direct=true&db=cin20&AN=128263365&site=ehost-live&scope=site | Ineligible study design (Not a guideline) |
| 20 | British Columbia Ministry of Health. Self-Management Support : A Health Care Intervention. British Columbia Ministry of Health [Internet]. 2011;http://www.selfmanagementbc.ca/uploads/What%20is%2. Available from: http://www.selfmanagementbc.ca/uploads/What is Self-Management/PDF/Self-Management Support A health care intervention 2011.pdf | Ineligible population (Adults with TB-HIV, TB, HIV, Chronic condition) |
| 21 | British HIV Association. Treatment of HIV-1 positive adults with antiretroviral therapy. British HIV Association [Internet]. 2014; Available from: http://www.bhiva.org/documents/Guidelines/Treatment/2012/hiv1029_2.pdf | Duplicate study |
| 22 | British HIV Association. Treatment of HIV-1-positive adults with antiretroviral therapy (interim update). British HIV Association [Internet]. 2017 Jan; Available from: https://www.bhiva.org/file/RVYKzFwyxpgiI/treatment-guidelines-2016-interim-update.pdf | Ineligible intervention/phenomena of interest (Not self-management support) |
| 23 | CDC. Managing Drug Interactions in the Treatment of HIV-Related Tuberculosis. 2013; | Ineligible intervention/phenomena of interest (Not self-management support) |
| 24 | Cabotegravir with rilpivirine for treating HIV-1. 2022;(January):27. | Ineligible intervention/phenomena of interest (Not self-management support) |
| 25 | Campbell JR, Pease C, Daley P, Pai M. Chapter 4 : Diagnosis of tuberculosis infection. Canadian Journal of Respiratory, Critical Care, and Sleep Medicine [Internet]. 2022;6(S1):65. Available from: https://doi.org/10.1080/24745332.2022.2036503 | Ineligible intervention/phenomena of interest (Not self-management support) |
| 26 | Centers for Disease Control and Prevention. Guidelines for the Prevention and Treatment of Opportunistic Infections in Adults and Adolescents with HIV. 2021; | Ineligible intervention/phenomena of interest (Not self-management support) |
| 27 | Compendium of good practices in the implementation of the Tuberculosis Action Plan for the WHO European Region 2016–2020. 2020; | Ineligible intervention/phenomena of interest (Not self-management support) |
| 28 | Consolidated guidelines on HIV prevention, testing, treatment, service delivery and monitoring: recommendations for a public health approach. World Health Organisation Guidelines [Internet]. 2021 Jun; Available from: https://apps.who.int/iris/rest/bitstreams/1357089/retrieve | Ineligible intervention/phenomena of interest (Not self-management support) |
| 29 | Cornick R, Fairall L. Practical Approach to Care Kit (PACK) - Primary Care Guide for the Adult (Western Cape Edition). 2021; | Ineligible intervention/phenomena of interest (Not self-management support) |
| 30 | DATA MANAGEMENT Services to Persons Living with HIV and AIDS ( PLHIV ). 2017;(September). | Ineligible intervention/phenomena of interest (Not self-management support) |
| 31 | Dyer M, Kerr C. Comprehensive Primary Care for Adults With HIV. 2021; | Ineligible intervention/phenomena of interest (Not self-management support) |
| 32 | Edition S. GUIDELINES FOR HIV SURVEILLANCE AMONG TUBERCULOSIS PATIENTS. | Document older than 1st January 2010 |
| 33 | Edition S. Malawi National Tuberculosis Control Programme Manual. 2012; | Ineligible intervention/phenomena of interest (Not self-management support) |
| 34 | World Health Organization. 3^rd^ Edition. Treatment Of Tuberculosis: Guidelines for National Programmes. 2003 | Document older than 1st January 2010 |
| 35 | Edwards N, Kahwa E, Hoogeveen K. Results of an Integrative Analysis: A Call for Contextualizing HIV and AIDS Clinical Practice Guidelines to Support Evidence-Based Practice. Worldviews on Evidence-Based Nursing [Internet]. 2017;14(6):498. Available from: https://search.ebscohost.com/login.aspx?direct=true&db=cin20&AN=126598923&site=ehost-live&scope=site | Ineligible study design (Not a guideline) |
| 36 | Erin Martz. Promoting Self-management of Chronic Health Conditions. 2017. | Ineligible study design (Not a guideline) |
| 37 | Feyissa GT, Lockwood C, Woldie M, Munn Z. Reducing HIV-related stigma and discrimination in healthcare settings: A systematic review of guidelines, tools, standards of practice, best practices, consensus statements and systematic reviews. Journal of Multidisciplinary Healthcare [Internet]. 2018;11:416. Available from: https://www.scopus.com/inward/record.uri?eid=2-s2.0-85058987157&doi=10.2147%2FJMDH.S170720&partnerID=40&md5=8640b40b4c128564714cba7d6964075b | Ineligible study design (Not a guideline) |
| 38 | Garrett D. Practice question: Promoting self-management. Nursing Older People [Internet]. 2012;24(4):10. Available from: https://search.ebscohost.com/login.aspx?direct=true&db=cin20&AN=75126672&site=ehost-live&scope=site | Ineligible study design (Not a guideline) |
| 39 | Ghana AIDS Commission. National HIV & AIDS Strategic Plan: 2016 to 2020. 2016; | Ineligible study design (Not a guideline) |
| 40 | Ghana AIDS Commission. National HIV Homebased Care Training Manual. 2016; | Ineligible study design (Not a guideline) |
| 41 | Ghana AIDS Commission. National HIV and AIDS Strategic Plan 2016-2020. 2016;400. | Ineligible study design (Not a guideline) |
| 42 | Ghana Aids Commission. National Hiv/Aids and Sti Policy. 2004;(August):33. | Ineligible study design (Not a guideline) |
| 43 | Ghana Health Service. Guidelines for the Clinical Management of TB and HIV Co-infection in Ghana. 2007;(July). | Document older than 1st January 2010 |
| 44 | Ghana Health Service. Implementation of TB/HIV collaborative activities in Ghana: joint programme planning policy and guidelines. 2014;(March). | Ineligible intervention/phenomena of interest (Not self-management support) |
| 45 | Ghana Health Service. Technical Policy and Guidelines for TB/HIV Collaboration in Ghana. 2007; Available from: http://www.tbonline.info/media/uploads/documents/implementation_of_hiv:tb_collaborative_activities_for_ghana-_technical_policy_and_guidelines_%282007%29.pdf | Ineligible study design (Not a guideline) |
| 46 | Ghana Ministry of Health. Ghana Health Service (2016) Guideline for ANtiretroviral Therapy in Ghana.pdf. 2016; | Ineligible intervention/phenomena of interest (Not self-management support) |
| 47 | Guide AP. TB-HIV Activities. 2012; | Ineligible study design (Not a guideline) |
| 48 | Guidelines for the management of Tuberculosis , Human Immunodeficiency Virus and Sexually-Transmitted Infections in Correctional facilities 2013. 2013; | Ineligible intervention/phenomena of interest (Not self-management support) |
| 49 | Guidelines on the management of latent tuberculosis infection. World Health Organisation Guidelines [Internet]. 2015 Apr; Available from: http://apps.who.int/iris/bitstream/10665/136471/1/9789241548908_eng.pdf?ua=1 | Ineligible intervention/phenomena of interest (Not self-management support) |
| 50 | Guidelines on the treatment of skin and oral HIV-associated conditions in children and adults. | Ineligible intervention/phenomena of interest (Not self-management support) |
| 51 | HIV and adolescents: Guidance for HIV testing and counselling and care for adolescents living with HIV. World Health Organisation HIV Guidelines [Internet]. 2013; Available from: https://apps.who.int/iris/handle/10665/94334 | Ineligible intervention/phenomena of interest (Not self-management support) |
| 52 | HIV testing: Increasing uptake among people who may have undiagnosed HIV. 2021;(December 2016):39. | Ineligible intervention/phenomena of interest (Not self-management support) |
| 53 | HIV, viral hepatitis and STIs - a guide for primary care. Clinical Practice Guidelines Portal [Internet]. 2014; Available from: https://ashm.blob.core.windows.net/ashmpublic/HIV_Viral_Hepatitis_and_STIs_a_Guide_for_Clinical_Care_(4th_Edition).pdf | Ineligible intervention/phenomena of interest (Not self-management support) |
| 54 | Hargreaves N, Scano F. Guidelines for implementing collaborative TB and HIV programme activities. 2003; Available from: http://apps.who.int/iris/bitstream/10665/42677/1/WHO_CDS_TB_2003.319_eng.pdf | Document older than 1st January 2010 |
| 55 | Health M. Communicable Disease Control Manual. 2012; | Ineligible study design (Not a guideline) |
| 56 | Health M. Guidelines for Tuberculosis Control in New Zealand ,. 2019; | Ineligible intervention/phenomena of interest (Not self-management support) |
| 57 | Health M. Tuberculosis Case Management for People in Correctional Facilities. 2018; | Ineligible study design (Not a guideline) |
| 58 | Health R. WHO Consolidated Guideline on Self-Care Interventions for Health. | Duplicate study |
| 59 | Holmes CB, Sanne I. Changing models of care to improve progression through the HIV treatment cascade in different populations. Current Opinion in HIV and AIDS [Internet]. 2015;10(6):450. Available from: https://www.scopus.com/inward/record.uri?eid=2-s2.0-84943740321&doi=10.1097%2FCOH.0000000000000194&partnerID=40&md5=8c4cb1cb704f8693a984addf76cdea04 | Ineligible study design (Not a guideline) |
| 60 | Humphreys M. Living Well with a Chronic Condition : Framework for Self-management Support. | Ineligible study design (Not a guideline) |
| 61 | Indicators HL. THE END TB STRATEGY : MAIN INDICATORS IN THE. 2020; | Ineligible study design (Not a guideline) |
| 62 | International FH. Collaborative TB / HIV Services. 2009;(October). | Document older than 1st January 2010 |
| 63 | Iseases D. District Clinician Manual : Hospital Care for Adolescents and Adults. 2021;1(January). | Ineligible intervention/phenomena of interest (Not self-management support) |
| 64 | J B, E M, P R. Primary immunodeficiency disease: a model for case management of chronic diseases. Professional Case Management [Internet]. 2010;15(1):14. Available from: https://search.ebscohost.com/login.aspx?direct=true&db=cin20&AN=105275699&site=ehost-live&scope=site | Ineligible study design (Not a guideline) |
| 65 | Jackie Smith. The 90-90-90 Compendium: Healthy me, healthy us: A guide for community members about good health and staying healthy with chronic illness. 4th ed. 2018. | Ineligible intervention/phenomena of interest (Not self-management support) |
| 66 | Jelliman P. Finding and managing patients living with hiv lost to care: A guide for nurses. HIV Nursing [Internet]. 2020;20(1):BP4. Available from: https://www.scopus.com/inward/record.uri?eid=2-s2.0-85085507203&partnerID=40&md5=c70a54f05145cddefd893990feeebe23 | Ineligible study design (Not a guideline) |
| 67 | Johnston JC, Cooper R, Menzies D. Chapter 5 : Treatment of tuberculosis disease. Canadian Journal of Respiratory, Critical Care, and Sleep Medicine [Internet]. 2022;6(S1):76. Available from: https://doi.org/10.1080/24745332.2022.2036504 | Ineligible study design (Not a guideline) |
| 68 | Ka’opua LS, Linsk NL. HIV treatment adherence: Challenges for social services. HIV Treatment Adherence: Challenges for Social Services [Internet]. 2013;184. Available from: https://www.scopus.com/inward/record.uri?eid=2-s2.0-84905780593&doi=10.4324%2F9780203052075&partnerID=40&md5=e2683199236d32d8cd98a507f2d00dd9 | Ineligible intervention/phenomena of interest (Not self-management support) |
| 69 | Kelly AM. Tuberculosis. Nursing Clinics of North America [Internet]. 2019 Jan;54(2):205. Available from: https://search.ebscohost.com/login.aspx?direct=true&db=awn&AN=31027661&site=ehost-live&scope=site | Ineligible study design (Not a guideline) |
| 70 | LeRoy L, Shoemaker SJ, Levin JS, Weschler CA, Schaefer J, Genevro JL. Self-management support resources for nurse practitioners and clinical teams. Journal for Nurse Practitioners [Internet]. 2014;10(2):93. Available from: https://search.ebscohost.com/login.aspx?direct=true&db=cin20&AN=107889715&site=ehost-live&scope=site | Ineligible study design (Not a guideline) |
| 71 | Lin Y. MELLITUS-TUBERCULOSIS A Guide to the Essential Practice MELLITUS-TUBERCULOSIS A Guide to the Essential Practice First Edition. 2019; | Ineligible intervention/phenomena of interest (Not self-management support) |
| 72 | Management of MDR-TB: A Field Guide. Management of MDR-TB: A Field Guide: A Companion Document to Guidelines for Programmatic Management of Drug-Resistant Tuberculosis: Integrated Management of Adolescent and Adult Illness (IMAI) [Internet]. 2009; Available from: http://www.ncbi.nlm.nih.gov/pubmed/26290923 | Document older than 1st January 2010 |
| 73 | Management of collaborative TB / HIV activities : Training for managers at the national and subnational levels. | Document older than 1st January 2010 |
| 74 | Mburu G, Richardson D. Community-based TB and HIV integration. 2013; | Ineligible study design (Not a guideline) |
| 75 | Medicines Adherence : involving patients in decisions about prescribed medicines and supporting adherence Full Guideline January 2009 National Collaborating Centre for Primary. 2009;(January). | Document older than 1st January 2010 |
| 76 | Module O. Self-Management Support Module User Guide Reducing Barriers to Quality Assurance in Primary Care via Self-Management Support Module User Guide. | Ineligible study design (Not a guideline) |
| 77 | Morillo-Verdugo R, Polo R, Knobel H. Consensus document on enhancing medication adherence in patients with the human immunodeficiency virus receiving antiretroviral therapy. Farmacia Hospitalaria [Internet]. 2020;44(4):173. Available from: https://www.scopus.com/inward/record.uri?eid=2-s2.0-85087800041&doi=10.7399%2Ffh.11441&partnerID=40&md5=d0fc013bad772a263875587cfbc45914 | Ineligible study design (Not a guideline) |
| 78 | Murphy KM, Mash R, Malan Z. The case for behavioural change counselling for the prevention of NCDs and improvement of self-management of chronic conditions. South African Family Practice [Internet]. 2016;58(6):252. Available from: https://search.ebscohost.com/login.aspx?direct=true&db=cin20&AN=120131019&site=ehost-live&scope=site | Ineligible study design (Not a guideline) |
| 79 | Médecins Sans Frontières. Clinical guidelines - Diagnosis and treatment manual. 2022. | Ineligible intervention/phenomena of interest (Not self-management support) |
| 80 | NATIONAL INSTITUTE FOR HEALTH AND CARE Older people with social care needs and multiple long - term conditions. 2020; | Duplicate study |
| 81 | NICE (Internal Clinical Guidelines Team). Tuberculosis. 2016. | Ineligible intervention/phenomena of interest (Not self-management support) |
| 82 | NICE. Tuberculosis. 2017. | Ineligible intervention/phenomena of interest (Not self-management support) |
| 83 | NICE. Tuberculosis. 2019. | Ineligible intervention/phenomena of interest (Not self-management support) |
| 84 | Nasmith L, Kupka S, Ballem P, Creede C. Achieving care goals for people with chronic health conditions. Can Fam Physician [Internet]. 2013;59(1):13. Available from: http://www.cfp.ca/content/59/1/11.long | Ineligible study design (Not a guideline) |
| 85 | National HIV and AIDS Policy. 2019;(September). | Document older than 1st January 2010 |
| 86 | National Institute for Health and Care Excellence. NICE Tuberculosis. Nice Guideline 33. 2016;9(2):178. | Duplicate study |
| 87 | National Strategic Plan to Reduce Human Rights- Related Barriers to HIV and TB Services : Ghana. 2020;(May). | Ineligible study design (Not a guideline) |
| 88 | National Tuberculosis Control Programme. National TB Control Program Training Manual. 2012;173. Available from: http://www.tbghana.gov.gh/sites/default/files/NTP Training Manual 2012.pdf | Ineligible study design (Not a guideline) |
| 89 | New Zealand Ministry of Health. Guidance on Infectious Disease Management under the Health Act 1956. 2017; | Ineligible intervention/phenomena of interest (Not self-management support) |
| 90 | New Zealand Ministry of Health. REVIEW OF SERVICES FOR PEOPLE LIVING WITH HIV IN NEW ZEALAND. 2010; | Ineligible study design (Not a guideline) |
| 91 | New Zealand Ministry of Health. Self-management Support for People with Long-term Conditions. 2016; | Ineligible intervention/phenomena of interest (Not self-management support) |
| 92 | Nursing Guide for Managing Side Effects to Drug-resistant TB Treatment. | Ineligible intervention/phenomena of interest (Not self-management support) |
| 93 | O Niel J. A Clinical Guide to Supportive and Palliative Care for HIVAIDS. 1999; | Document older than 1st January 2010 |
| 94 | Older people with social care needs and multiple long-term conditions. 2021;(November 2015). | Ineligible population (Adults with TB-HIV, TB, HIV, Chronic condition) |
| 95 | Organization WH, Office R. Management of tuberculosis and HIV coinfection (Eng). 2013; | Ineligible study design (Not a guideline) |
| 96 | Organization WH. WHO Guideline on self-care interventions for health and well-being WHO Guideline on self-care interventions for health. 2021; | Duplicate study |
| 97 | O’Byrne P, MacPherson P, Orser L, Jacob JD, Holmes D. PrEP-RN: Clinical Considerations and Protocols for Nurse-Led PrEP. JANAC: Journal of the Association of Nurses in AIDS Care [Internet]. 2019;30(3):311. Available from: https://search.ebscohost.com/login.aspx?direct=true&db=cin20&AN=139173851&site=ehost-live&scope=site | Ineligible study design (Not a guideline) |
| 98 | Panel D, Guidelines A, Group W, Council A. Guidelines for the Use of Antiretroviral Agents in Adults and Adolescents with HIV Developed by the DHHS Panel on Antiretroviral Guidelines for Adults. | Ineligible intervention/phenomena of interest (Not self-management support) |
| 99 | Participant training manual for the three interlinked patient monitoring systems for HIV care / ART , MCH / PMTCT , and TB / HIV I nter l inked P atient M onitoring S ystem. 2012; | Ineligible study design (Not a guideline) |
| 100 | Patient Self-Management Support of Chronic Conditions : Framework for Clinicians Seeking Recertifcation Credit ( MOC Part IV. 2022; | Ineligible population (Adults with TB-HIV, TB, HIV, Chronic condition) |
| 101 | Patient experience in adult NHS services : improving the experience of care for people using adult NHS services. 2021;(February 2012). Available from: https://drive.google.com/file/d/1YhwphQvGS3ND47k-RYwcPviMxRe2u71l/view?usp=sharing | Ineligible intervention/phenomena of interest (Not self-management support) |
| 102 | Primary Care Guidance for Persons With Human Immunodeficiency Virus: 2020 Update by the HIV Medicine Association of the Infectious Diseases Society of America. Infectious Diseases Society of America [Internet]. 2020 Oct; Available from: https://academic.oup.com/cid/advance-article-pdf/doi/10.1093/cid/ciaa1391/34463416/ciaa1391.pdf | Ineligible intervention/phenomena of interest (Not self-management support) |
| 103 | Programme D. Standard Treatment Guidelines. 2017; | Ineligible intervention/phenomena of interest (Not self-management support) |
| 104 | Region WHOE. Psychosocial support for people with TB , HIV and viral hepatitis in the continuum of care in the WHO European Region Report - July 2020. 2020;(July). | Ineligible intervention/phenomena of interest (Not self-management support) |
| 105 | Republic of Phillipines National Tuberculosis Control Program. Manual for procedures. 2020; | Ineligible study design (Not a guideline) |
| 106 | Ridgely MS. Patient Self-Management Support Programs : An Evaluation. (8). | Ineligible intervention/phenomena of interest (Not self-management support) |
| 107 | Rottenberg ME, Pawlowski A, Jansson M, Sko M. Tuberculosis and HIV Co-Infection. 2012;8(2). | Ineligible study design (Not a guideline) |
| 108 | Self-management guide book. | Ineligible population (Adults with TB-HIV, TB, HIV, Chronic condition) |
| 109 | Services USP, Force T. Primary Care Screening and Treatment for Latent Tuberculosis Infection in Adults. 2016;27709. | Ineligible study design (Not a guideline) |
| 110 | Services USP, Force T. Screening for Latent Tuberculosis Infection in Adults. 2016;316(9). | Ineligible study design (Not a guideline) |
| 111 | Southern African HIV Clinicians Society / UNHCR. Clinical Guidelines for antiretroviral therapy management for displaced populations Southern Africa. 2007. | Ineligible intervention/phenomena of interest (Not self-management support) |
| 112 | Steps to Care. HIV Self Management Tools Chart. | Ineligible study design (Not a guideline) |
| 113 | Stop TB Partnership. People living with hiv. | Ineligible study design (Not a guideline) |
| 114 | Supporting people to. 2012;(August). | Ineligible population (Adults with TB-HIV, TB, HIV, Chronic condition) |
| 115 | Supporting self-management Key themes. | Ineligible study design (Not a guideline) |
| 116 | The National Tuberculosis and Leprosy Control Program Zambia. TB Manual. 2017. | Ineligible study design (Not a guideline) |
| 117 | Therapy MN. Recommendations Summary. 2010;21. | Ineligible study design (Not a guideline) |
| 118 | Transition between inpatient hospital settings and community or care home settings for adults with social care needs. 2022;(December 2015):32. | Ineligible population (Adults with TB-HIV, TB, HIV, Chronic condition) |
| 119 | Treatment HIV. GLOBAL UPDATE ON HIV TREATMENT 2013 : 2013;(June). | Ineligible study design (Not a guideline) |
| 120 | Tsima BM, Setlhare V, Nkomazana O. Developing the Botswana Primary Care Guideline: An integrated, symptom-based primary care guideline for the adult patient in a resource‑limited setting. Journal of Multidisciplinary Healthcare [Internet]. 2016;9:354. Available from: https://www.scopus.com/inward/record.uri?eid=2-s2.0-84987864014&doi=10.2147%2FJMDH.S112466&partnerID=40&md5=0627b9066d0ddbafc1cc749173612d72 | Ineligible intervention/phenomena of interest (Not self-management support) |
| 121 | Tuberculosis Programme. 2001; | Document older than 1st January 2010 |
| 122 | Use of mHealth Technology for Supporting Symptom Management in Underserved Persons Living with HIV - Final Report. 2018; | Ineligible population (Adults with TB-HIV, TB, HIV, Chronic condition) |
| 123 | WHO consolidated guidelines on tuberculosis . Module 3 : diagnosis – rapid diagnostics for tuberculosis detection Web Annex 3 . Evidence-to-decision tables 2021 update. 2021; | Ineligible intervention/phenomena of interest (Not self-management support) |
| 124 | WHO consolidated guidelines on tuberculosis: module 1: prevention: tuberculosis preventive treatment. World Health Organisation Guidelines [Internet]. 2020; Available from: https://apps.who.int/iris/rest/bitstreams/1270183/retrieve | Ineligible study design (Not a guideline) |
| 125 | WHO. WHO Consolidated Guideline on Self-Care Interventions for Health: Sexual and Reproductive Health and Rights. Omega [Internet]. 2019 Jun;23(3):107. Available from: http://dx.doi.org/10.1016/j.ijmedinf.2011.08.007%0Ahttp://www.ncbi.nlm.nih.gov/pubmed/28348205%0Ahttp://www.pubmedcentral.nih.gov/articlerender.fcgi?artid=PMC5485755%0Ahttp://jech.bmj.com/lookup/doi/10.1136/jech-2016-208322%0Ahttp://dx.doi.org/10.1016/j.a | Duplicate study |
| 126 | Wold Health Organization. IMAI District Clinician Manual: Hospital Care for Adolescents and Adults: Guidelines For The Management Of Common Illnesses With Limited Resources_Vol. 2. 2011. | Duplicate study |
| 127 | World Health Organization (WHO). Updated recommendations on service delivery for the treatment and care of people living with HIV. World Health Organisation Guidelines [Internet]. 2021 Jun;61. Available from: https://apps.who.int/iris/rest/bitstreams/1344311/retrieve | Ineligible intervention/phenomena of interest (Not self-management support) |
| 128 | World Health Organization, Geneva WHO, Organization WH, others, World Health Organization. WHO policy on collaborative TB/HIV activities: guidelines for national programmes and other stakeholders. 2012; | Ineligible intervention/phenomena of interest (Not self-management support) |
| 129 | World Health Organization. Compendium of WHO guidelines and associated standards: ensuring optimum delivery of the cascade of care for patients with tuberculosis. 2018;(June). Available from: https://apps.who.int/iris/bitstream/handle/10665/272644/9789241514101-eng.pdf?ua=1 | Ineligible intervention/phenomena of interest (Not self-management support) |
| 130 | World Health Organization. Consolidated guidelines on the use of antiretroviral drugs for treating and preventing HIV infection. World Health Organization [Internet]. 2016;152, 402. Available from: https://apps.who.int/iris/bitstream/handle/10665/208825/9789241549684_eng.pdf?sequence=1&isAllowed=y | Duplicate study |
| 131 | World Health Organization. Consolidated guidelines on tuberculosis. 2020; | Ineligible intervention/phenomena of interest (Not self-management support) |
| 132 | World Health Organization. Facilitator’s Guide for the IMAI TB-HIV Co-management Training Course. | Duplicate study |
| 133 | World Health Organization. General Principles of Good Chronic Care (INTEGRATED MANAGEMENT OF ADOLESCENT AND ADULT ILLNESS). 2004. | Document older than 1st January 2010 |
| 134 | World Health Organization. Guideline for Managing Advanced of Antiretroviral Rapid Initiation HIV Disease and Therapy. 2017. | Ineligible intervention/phenomena of interest (Not self-management support) |
| 135 | World Health Organization. Guideline on When To Start Antiretroviral Therapy and on Pre-Exposure Prophylaxis for HIV. World Health Organisation HIV Guidelines [Internet]. 2015 Oct;(September):76. Available from: http://apps.who.int/iris/bitstream/10665/186275/1/9789241509565_eng.pdf?ua=1 | Ineligible intervention/phenomena of interest (Not self-management support) |
| 136 | World Health Organization. Guidelines for treatment of drug-susceptible tuberculosis and patient care. 2017; Available from: http://www.who.int/tb/publications/ 2017/dstb_guidance_2017/en/ | Ineligible intervention/phenomena of interest (Not self-management support) |
| 137 | World Health Organization. IMAI District Clinician Manual: Hospital Care for Adolescents and Adults: Guidelines For The Management Of Common Illnesses With Limited Resources_Vol. 1. 2011. | Duplicate study |
| 138 | World Health Organization. Operations Manual for Delivery of HIV Prevention, Care and Treatment at Primary Health Centres in High-Prevalence, Resource-Constrained Settings. 2008. | Ineligible study design (Not a guideline) |
| 139 | World Health Organization. Recommendations for HIV-prevalent and resource-constrained settings. 2007; | Document older than 1st January 2010 |
| 140 | World Health Organization. TB/HIV Coinfection Regional Clinical Manual 2017. 2017; | Ineligible intervention/phenomena of interest (Not self-management support) |
| 141 | World Health Organization. THREE INTERLINKED PATIENT MONITORING SYSTEMS FOR HIV CARE / ART , MCH / PMTCT AND TB / HIV. 2012. | Ineligible study design (Not a guideline) |
| 142 | World Health Organization. Treatment of tuberculosis: guidelines – 4th ed. WHO/HTM/TB/2009.420. WHO Library Cataloguing-in-Publication Data: [Internet]. 2010;95(34–36):160. Available from: https://www.ncbi.nlm.nih.gov/books/NBK138748/pdf/Bookshelf_NBK138748.pdf | Document older than 1st January 2010 |
| 143 | World Health Organization. Tuberculosis care with TB-HIV co-management: Integrated management of adolescent and adult illness. 2007;34. | Document older than 1st January 2010 |
| 144 | World Health Organization. WHO Guideline on Self-Care Interventions for Health and Well-Being. 2021; | Ineligible intervention/phenomena of interest (Not self-management support) |
| 145 | World Health Organization. WHO recommendations on self-care interventions. 2020; | Duplicate study |
| 146 | World Health Organization. A guide to monitoring and evaluation for collaborative TB/HIV activities. 2015; | Ineligible intervention/phenomena of interest (Not self-management support) |
| 147 | World Health Organization. Antiretroviral therapy for HIV Infection in Adults And Adolescents: Recommendations for a public health approach. 2010; | Ineligible intervention/phenomena of interest (Not self-management support) |
| 148 | World Health Organization. Management of Tuberculosis Training for Health Facility Staff. 2010; | Ineligible study design (Not a guideline) |
| 149 | World Health Organization. NTP Manual Timor-Leste. 2020; | Ineligible study design (Not a guideline) |
| 150 | World Health Organization. Participant’s Manual for the IMAI TB-HIV Co-management Training Course. 2008; | Document older than 1st January 2010 |
| 151 | World Vision. Technical Guideline for Tuberculosis ( TB ) and TB-HIV Program Implementation. 2017;28. | Ineligible intervention/phenomena of interest (Not self-management support) |
