# Supplementary material for: Best practice guidelines for professional nurses to provide self-management support to adults with tuberculosis-human immunodeficiency virus coinfection: A scoping review
Source: PLoS One. 2023 Sep 12;18(9):e0291529. doi: 10.1371/journal.pone.0291529 (PMC10497153; doi:10.1371/journal.pone.0291529)
Supplement: S3 Appendix — (DOCX) [file pone.0291529.s004.docx]

**S3 APPENDIX**

**Recommendations and underlying evidence** **in the high-quality guideline**

**Table S3. Characteristics of recommendations in high-quality guideline.**

| **Recommendation** | **Source of evidence** | **Population** | **Design, methods and groups** | **Summary of main findings and effects** |
| --- | --- | --- | --- | --- |
| **A. PRACTICE RECOMMENDATIONS** | | | | |
| (1) Nurses utilize the “5 A’s” behavioural change approach of assess, advise, agree, assist and arrange, to incorporate multiple self-management strategies when supporting clients with a chronic illness to assist in improved outcomes. | Pignone et al., (2003) [1] | Adult males and females (≥18 years) with diet behaviour challenges | Systematic Review of 21 randomised controlled trials  **Intervention**: interactive communication  **Outcome**: change in diet behaviour.  **Setting**: primary healthcare | Interventions using interactive health communications, including computer-generated telephone or mail messages, produce moderate dietary changes. |
|  | McTigue et al., (2003) [2] | Adults with obesity | Systematic review of systematic reviews, Randomised controlled trials, and observational studies.  **Intervention**: Counseling and pharmacotherapy  **Outcome**: weight loss  **Setting**: primary healthcare | Counselling and pharmacotherapy can promote modest sustained weight loss, improving clinical outcomes. |
|  | Whitlock et al., (2002) [3] | Adults with risky behaviour | Systematic review of interventions  **Intervention**: behavioural counselling interventions  **Outcome**: change in behaviour  **Setting**: primary healthcare | Use of the 5A model (Assess, Advise, Agree, Assist, Arrange) during behavioural counselling can contribute to behaviour change. |
|  | Fiore et al., (2000) [4] | Persons who use tobacco | Clinical guideline for treating tobacco use and dependence | Multiple modalities contribute to improved health behaviour outcomes |
|  | Gibson et al., (2002) [5] | Adults (≥16 years) with asthma | Systematic review of 12 Randomised controlled trials  **Intervention**: limited asthma education.  **Outcome**: improving health outcomes  **Setting**: healthcare setting | Limited asthma education improved perceived asthma symptoms (OR 0.44, 95%CI 0.26 to 0.74) and reduced emergency department visits (-2.76 average visits per person per year, 95% confidence interval -4.34 to 1.18).  Intervention did not reduce hospitalisation for asthma, doctor visits, lung function and medication use |
|  | Toelle & Ram (2004) [6] | Adults and Children with Asthma | Systematic review of 7 Randomised controlled trials  **Intervention**: education, individualised written asthma self-management plan  **Outcomes**: medication adherence, hospitalisation, emergency department visits, oral corticosteroid use, lung function, days lost from school/work, unscheduled doctor visits and respiratory tract infections  **Setting:** primary healthcare | Inconsistent findings regarding improvement of outcomes. |
|  | Fahey, Schroeder, & Ebrahim (2005) [7] | Adults with hypertension | Systematic review of 59 Randomised controlled trials  **Intervention**: registration, recall and regular review stepped care  **Outcome**: control of blood pressure in clients with hypertension  **Setting**: primary healthcare | Use of multiple interventions improved patients’ health outcomes. |
|  | Deakin, McShane, Cade & Williams (2005) [8] | Adults with type 2 diabetes | Systematic review of 11 randomised controlled trials  **Intervention**: group-based, patient-centred training  **Outcome**: levels of glycated haemoglobin, fasting blood glucose, body weight, diabetes knowledge, need for diabetes medication  **Setting**: primary healthcare | The intervention contributed to improved health outcomes. |
|  | Goldstein (2004) [9] | Adults with Diabetes Mellitus | Literature review based on chronic care model  **Intervention**: Self-management support | multifaceted interventions are more effective in improving health behaviours compared to isolated interventions. |
|  | Cohen, Tallia, Crabtree & Young (2005) [10] | Adults with Diabetes Mellitus | **Intervention**: Systematic Review and content analysis of participants’ 17 diary data.  **Setting**: primary healthcare | Multifaceted interventions are more effective in promoting healthy behaviours than those isolated interventions |
|  | Mullen et al., (1997) [11] | Persons without diagnosed diseases (drug addicts, nutrition | Systematic review of 74 randomised controlled trials and non-randomised controlled trials.  **Intervention**: Individual and group psychosocial and psychological interventions (health education, counselling and/or cognitive-behavioural stress management)  **Outcome**: preventive health behaviours  **Setting**: primary healthcare | Behavioural interventions including self-monitoring and utilization of multiple communication channels yielded larger effects among participants who belong to nutrition groups or abuse smoke /alcohol. |
|  | Glasgow et al., (2003) [12] | Persons with chronic conditions | Article on self-management support models  **Setting**: primary healthcare | Self-management support is iterative, individualised patient-centred and requires collaborative goal setting |
|  | Pignone et al., (2002) [13] | Adults with depression | Systematic review of Randomised controlled trials  **Intervention**: feedback on depression screening  **Outcome**: levels and duration of depression  **Setting**: primary healthcare | Screening and feedback reduced participants’ risk for persistent depression (summary relative risk, 0.87 [95% CI, 0.79 to 0.95]).  Integrated interventions were more effective. |
|  | Renders et al., (2000) [14] | Persons with Diabetes including adults. | Systematic review of 27 randomised controlled trials, 12 controlled before and after studies and 2 interrupted time series.  **Interventions**: computers or nurses contacting patients, postgraduate education, reminders, audit and feedback, local consensus processes, and peer review  **Outcome**: health professional performance and patient glycaemic control, cardiovascular risk factors, wellbeing  **Setting**: primary healthcare, outpatient and community | Multifaceted professional and organizational interventions can enhance the performance of care providers. |
|  | Rubak, Sandbaek, Lauritzen & Christensen (2005) [15] | Persons with chronic conditions | Systematic review of 72 randomised controlled trials  **Intervention**: effectiveness of motivational interview  **Outcome**: body mass index, total blood cholesterol, systolic blood pressure, cigarettes per day and A1C levels  **Setting**: primary/secondary healthcare | Intervention improved body mass index, total blood cholesterol, systolic blood pressure, but not cigarettes per day and gluacted haemoglobin (A1C) levels |
|  | Smith-West et al., (2007) [16] | Women with Type 2 Diabetes Mellitus (n = 217) | Randomised controlled trial  **Intervention**: effectiveness of group-based 18 months motivational interview (Goal setting and problem-solving)  **Outcome**: Weightloss and glycemic control (A1c)  **Setting**: primary healthcare, United States of America | Women in motivational interviewing lost significantly more weight at 6 months (*p* = 0.01) and 18 months (*p* = 0.04).  glycemic control (A1c) improved at 6 months (*p* =0.02) but not 8 months. |
|  | Solberg et al., (2000) [17] | Clinical guideline  implementation experts | Consensus through interviews and modified nominal group/Delphi process (n = 12). | Multiple strategies in guideline implementation are more effective than individual interventions |
|  | Woolf et al., (2005) [18] | Persons with chronic conditions | Focused review of program records and literature  **Intervention**: 17 Prescription for Health based on 5A model – Assess, Advice, Agree, Assist, Arrange  **Outcome**: behaviour change  **Setting**: primary healthcare, | Intervention facilitated patients’ (1) identification and change of unhealthy behaviours,  (2) access to information at home (3) use self-help methods,  (4) access to intensive counselling, and (5) follow-up |
|  | Bodenheimer & Grumbach (2007) [19] | Persons with chronic and acute conditions | Teamlet model of care  **Intervention**: Agenda setting, medication reconciliation, history taking, closing the loop, goal setting.  **Outcome**: informed and active self-management  **Setting**: primary healthcare | Multifaceted interventions can improve the health outcomes of patients with chronic conditions. |
|  | Goldstein, Whitlock, & DePue (2004) [20] | Persons with chronic conditions (diabetes and cardiovascular conditions) | Review of randomised controlled trials and non-randomised controlled trials  **Intervention**: patient assessment, self-monitoring, collaborative goal setting and active problem-solving, multiple contacts  **Outcome**: Improved health behaviour (reduction of lack of physical activity, unhealthy diet, obesity, cigarette smoking, and risky/harmful alcohol use)  **Setting**: primary healthcare | Use of multiple/integrated modalities contributes to improved health behaviour outcomes |
|  | Glasgow, Goldstein, Ockene & Pronk (2004) [21] | Persons with chronic conditions | Literature review  **Intervention**: multifaceted behaviour change support using the 5A (Assess, Advice, Agree, Assist and Arrange) approach  **Outcome**: change in health behaviour  **Setting**: primary healthcare | Sustainable multiple behavioural changes should be patient-centred, tailored, proactive, population-based, culturally proficient, multilevel, and ongoing. |
| **1.1 ASSESS** | | | | |
| (1.1.a) Nurses establish rapport with clients and families. | Canadian Nurses Association (2010) [22] | Professional nurses | Nursing practice statements | Therapeutic or helping relationship is a central component of nursing care |
|  | College of Nurses of Ontario (2006) [23] | Professional nurses | Nursing practice statements | Therapeutic or helping relationship is a central component of nursing care |
|  | O’Connor et al., (1985) [24] | Patients seeking care in clinics | Paper on patient health counselling | Building rapport with the patient enhances health counselling and can improve client satisfaction. |
|  | Mejo (1989) [25] | Nurse practitioners and professional nurses | Clinical case report of care provided | The therapeutic relationship between the nurse and client is a factor that affects the patient’s treatment outcome |
|  | Paley & Lawton (2001) [26] | Therapy and healthcare providers | Paper promoting evidence-based practice. | Evidence that the therapeutic relationship between the healthcare provider and patient promotes health outcome should not be ignored. |
|  | Stewart (1995) [27] | Physicians and patients | Literature review of 21 randomised controlled trials and analytic studies | Effective physician and patient communication contributes to improved health outcomes |
|  | Registered Nurses Association of Ontario (2006) [28] | Professional nurses | Best practice guideline for establishing therapeutic relationships  **Methods**: Formal consensus based on a discussion of evidence among stakeholders | Establishing rapport as part of care delivery ensures that nurses listen to the views of the patient and family. |
|  | Redman (2004) [29] | Persons with chronic diseases | Book on evidence-based standards for self-management of chronic disease. | Nurses should assess persons with chronic health conditions for distress, anger and frustration as part of the rapport-building process. |
|  | Skovlund et al., (2005) [30] | Persons living with diabetes mellitus | Survey of 5,000 persons with diabetes mellitus and 4000 diabetes care providers | Distress related to chronic conditions can impede self-management. |
|  | Peyrot et al., (2005) [31] | Persons living with diabetes mellitus | Cross-sectional survey  5104 persons with diabetes mellitus and 3827 healthcare providers in 13 countries | Psychosocial problems and distress among persons with chronic conditions (diabetes mellitus) can impede self-management. |
| (1.1.b) Nurses screen for depression on initial assessment, at regular intervals and advocate for follow-up treatment of depression. | Chapman & Gratz (2007) [32] | Persons with borderline personality disorder | Book on borderline personality disorder. | Persons living with chronic conditions experience depression |
|  | Moussavi et al., (2007) [33] | Persons with chronic diseases | Cross-sectional study among 245, 404 participants with chronic conditions including angina, arthritis, asthma and diabetes mellitus worldwide. | Depression exacerbates chronic disease and contributes to decrement in health thus must be addressed by healthcare providers. |
|  | Anderson et al., (2001) [34] | Persons with diabetes mellitus | Systematic Review of 42 Randomised controlled trials and non-Randomised controlled trials  **Concept:** prevalence of depression among persons with diabetes  **Outcome**: depression levels | persons living with diabetes experience depression. Prevalence of depression is twice as the general population (OR = 2.0, 95% CI 1.8–2.2). |
|  | Taveira et al., (2008) [35] | Persons with hypertension, diabetes, hyperlipidemia and tobacco use | Retrospective analysis of intervention  **Intervention** (pharmacist-led cardiovascular risk reduction clinic)  297 participants with mental health challenges and other chronic conditions  **Outcome**: Risk of cardiovascular events  **Setting**: Clinic, United Kingdom | Mental health conditions Depression reduces the effects of interventions among persons with diabetes by decreasing patients’ knowledge acquisition and medication adherence levels. |
|  | Cleveland Clinic (2010) [36] | Persons with chronic conditions | Expert opinion article by a Cleveland Clinic medical professional  **Setting**: Ohio, United States of America | A third of persons with chronic conditions medical condition experience depression |
|  | Unützer et al., (1997) [37] | Persons with chronic conditions | Prospective cohort study  2,558 participants  **Outcome**: Depressive symptoms  **Setting**: primary healthcare, United States of America. | Risk of depression increases with risk of medical co-morbidity and age. |
|  | Egede et al., (2005) [38] | Adults with and without diabetes | Population-based survey  10,025 participants  **Setting**: United States of America | 20% to 50% of older adults with chronic conditions suffer from depression.  Comorbidity of depression and diabetes mellitus is associated with an elevated risk of death compared to depression or diabetes morbidity. |
|  | Bodenheimer et al., (2002) [39] | Persons with chronic conditions | Expert opinion based on literature review | The risk of morbidity and mortality among persons with comorbid chronic conditions is increased when depression is not treated. |
|  | Park et al., (2004) [40] | Persons with diabetes mellitus | Cross-sectional correlation survey  168 persons with diabetes aged >30 years  **Setting**: primary healthcare, Korea | Depression impairs the ability of persons with Type 2 diabetes mellitus to self-manage or exhibit medication adherence. |
|  | Lerman et al., (2004) [41] | Persons with diabetes mellitus | Cross-sectional survey  172 persons with diabetes aged 30-75 years  **Setting**: primary healthcare, Mexico | Depression reduces the ability of persons with Type 2 diabetes mellitus to exhibit medication adherence. |
|  | Katon & Ciechanowski (2002) [42] | Persons with chronic conditions | Review article  Paper reviews the epidemiology and impact of depression on persons with chronic conditions | Depression can contribute to avoidant behaviours among persons with chronic conditions. |
|  | DiMatteo et al., (2000) [43] | Patients receiving treatment from physicians | Systematic review of correlation studies  on compliance to treatment, depression level and anxiety level  **Setting**: Clinic | Depressed patients are three times less likely to adhere to their prescribed treatment. |
|  | Schmitz et al., (2007) [44] | Persons with chronic conditions and depression | Population-based survey through telephone interviews.  46,262 participants with depression, chronic conditions and functional disability  **Setting**: Clinic, Canada | Higher levels of functional disability were observed among persons with chronic conditions who were depressed compared to persons with chronic conditions without depression. |
|  | Kroenke et al., (2003) [45] | Persons with depression | A 2-item questionnaire for assessing depression (PHQ-2) | Depression can be validly and reliably measured with the questionnaire |
| (1.1.c) Nurses establish a written agenda for appointments in collaboration with the client and family, which may include:  • Reviewing clinical data  • Discussing client’s experiences with self-management  • Medication administration  • Barriers/stressors  • Creating action plans  • Client education | Epstein et al., (2008) [46] | healthcare providers | Review article  Paper on strategies for healthcare providers to structure the medical encounter with patients  **Setting**: primary healthcare, Mexico | Allowing the patient to contribute to setting the agenda promotes patient involvement, satisfaction with care and behaviour change |
|  | Little et al., (2001) [47] | Patients seeking care from physicians. | Survey with questionnaire.  865 participants with depression, chronic conditions and functional disability  **Setting**: primary healthcare, United Kingdom | Patients seeking primary healthcare want good communication, partnership and health promotion from their healthcare provider. |
|  | Middleton et al., (2006) [48] | General practitioners and patients | Randomised controlled trial  **Intervention**: Education for general practitioners (n = 45), with an embedded clustered Randomised controlled trial of a patient agenda form (857 patients)  **Outcome**: Number of health problems identified, the time required to manage each problem, duration of consultations, number of problems raised after the doctor considered the consultation finished (“by the way” questions), and patient satisfaction.  **Setting**: primary healthcare, United Kingdom | Patient-completed agenda forms prolonged consultation but contributed to the identification of more health problems among patients seeking healthcare. |
|  | Winefield et al., (1995) [49] | General practitioner | Review paper of 210 transcripts on types of general practice consultation (psychosocial, complex, and straightforward) | Patients are more satisfied when psychosocial problems are identified and addressed during interaction with healthcare providers |
|  | Hornberger et al., (1997) [50] | Physicians and community care patients | Randomised controlled trial  **Intervention**: patient completion of a pre-visit questionnaire on the need for medical information, psychosocial assistance, therapeutic listening, general health advice, and biomedical treatment. Physicians discussed questionnaires with patients during the visit.  **Outcome**: visit duration, the content of discussion, patient and physician satisfaction.  **Setting**: primary healthcare | The use of pre-visit questionnaires may lead to conflict goals between responding to patients’ concerns and increasing patient healthcare costs. |
|  | Miller & Rollnick (2002) [51] | healthcare professionals | Book on motivational interviewing to overcome the challenges associated with behaviour change. | Collaboratively setting the agenda for a health visit is consistent with Motivational interviewing. |
| (1.1.d) Nurses consistently assess client’s readiness for change to help determine strategies to assist client’s readiness for change to help determine strategies to assist client with specific behaviours. | Edwards et al., (1999) [52] | Persons with diabetes mellitus | Paper on the care of persons living with diabetes mellitus by healthcare providers. | Traditional methods used by healthcare providers to conduct health education assume patients are ready to change their health-related behaviours. |
|  | Jones et al., (2003) [53] | Persons living with diabetes melllitus | Randomised controlled trial  **Intervention**: pathway to change intervention based on transtheoretical model of change among 1,029 persons with diabetes mellitus  **Outcome**: change readiness, self-care and diabetes control  **Setting**: primary healthcare | Only a minority of patients receiving healthcare are ready for change. |
|  | Prochaska et al., (1992) [54] | Persons with health behavioural change needs | A theory-based Stages of Change Model | The patient should be involved in the precomtemplation stage of behavioural change process |
|  | Keller (1997) [55] | Persons with health behavioural change needs | Paper on the care of persons with behavioural needs. | healthcare providers should consider patients’ perceptions of their behaviour and confidence to change. |
|  | Rollnick et al., (2001) [56] | Persons with health behavioural change needs | Book on Health behaviour change: A guide for practitioners | healthcare providers should consider patients’ perceptions of their behaviour and confidence to change. |
|  | Whitlock et al., (2002) [3] | Adults with risky behaviour | Systematic review of interventions  **Intervention**: behavioural counselling interventions  **Outcome**: change in behaviour  **Setting**: primary healthcare | healthcare providers should consider patients’ perceptions of their behaviour and confidence to change. |
|  | Bodenheimer et al., (2005) [57] | Persons living with chronic conditions | Book on strategies for healthcare providers to help people to manage their chronic conditions | An assessment of the patient’s readiness for change should be conducted to support their self-management. |
| (1.1.e) Nurses encourage clients to use health risk appraisal instruments; model the use of such tools, and discuss the results of the risk assessment with them at regular follow up | Research and Development Corporation (2003) [58] | Older adults with health problems | Literature review on effectiveness of health research assessment for adults with health problems | The use of health risk assessment among patients can improve health behaviour (exercise) and general health services. |
|  | Research and Development Corporation, (2000) [59] | Older adults with health problems | Literature review on effectiveness of health research assessment for adults with health problems | Health risk assessment strategies for patients must consider information provision, support and referrals to be effective. |
| **1.2 ADVICE** | | | | |
| (1.2.a) Nurses combine effective behavioural, psychosocial strategies and self-management education processes as part of delivering self-management support. | Hibbard et al., (2007) [60] | Persons with chronic conditions | Randomised controlled trial  **Intervention**: patient activation (including workshop training on dealing with frustration, pain, fatigue, nutrition, communication and medication use) among 479 persons with diabetes mellitus between 50-70 years old  **Outcome**: self-management behaviours (exercise, eating low-fat diet, managing stress)  **Setting**: community-based healthcare | Patient activation contributes to an improvement in patient health outcomes and desired health behaviours |
|  | Anderson & Funnell (2005) [61] | Persons living with diabetes mellitus | Essay based on insight and experience | Collaborative care for persons with diabetes mellitus requires patient empowerment by health care providers. |
|  | Tang et al., (2006) [62] | Persons with chronic conditions | Paper on patient personal health records, definitions, benefits and strategies to overcome barriers. | Use active learning principles and engage patients in their learning processes. |
|  | Krichbaum et al., (2003) [63] | Persons living with diabetes mellitus | Literature review of articles on factors related to effective self-management of diabetes mellitus. | Involve the persons with diabetes in their own care, guide their active learning, and explore their feeling about the disease. |
|  | Lorig et al., (2001) [64] | Persons with chronic disease | Longitudinal study design post randomised controlled trial (Chronic disease Self-management Program)  among 831 persons with heart/lung disease, stroke and arthritis aged 40 years and older.  Outcome: health status, health utilization and perceived self-efficacy.  **Setting**: community-based healthcare | Emergency room and outpatient visits and health distress were reduced and self-efficacy improved.  Teaching self-management skills can contribute to behaviour change. |
|  | Bodenheimer & Handley (2009) [65] | Adolescents and adults with chronic diseases | Literature review of 8 articles on  processes of engaging patients on goal-setting.  **Setting**: primary healthcare | Collaborative goal-setting between healthcare providers and persons with chronic condition with feedback contributes to change in health behaviour. |
|  | Norris et al., (2002) [66] | Adults with diabetes mellitus | Systematic review of 31 randomised controlled trials  **Interventions**: self-management education  **Outcome**: Glycated Haemoglobin levels  **Setting**: primary, community, home-based healthcare | Glycated haemoglobin levels reduced over time among the intervention group. Self-management education contributed to change in patients’ learned health behaviours. |
|  | Barlow et al., (2002) [67] | Persons with chronic diseases | Literature review of 145 studies on self-management approaches for persons with chronic conditions  **Setting**: multiple, including primary healthcare | Several studies on self-management approaches focused on the attitudes of persons with chronic conditions as well as self-efficacy and confidence. |
|  | Brown et al., (2005) [68] | Persons with diabetes mellitus | Comparative study of 2 self-management interventions among 216 persons with diabetes mellitus aged 35 to 70 years. | Culturally appropriate self-management education interventions contribute to improved knowledge of diabetes and improved metabolic control. |
|  | Anderson & Funnell (2005) [61] | Persons living with diabetes mellitus | Essay based on insight and experience generated in the care of persons with diabetes mellitus. | Self-management Interventions tailored to the clients are more effective. |
|  | Sarkisian et al., (2003) [69] | Adults with diabetes mellitus | Systematic Review of 8 Randomised controlled trials  **Interventions**: self-management interventions including education.  **Outcome**: change health behaviour, knowledge and beliefs  **Setting**: primary healthcare | Self-management Interventions tailored to the clients’ culture or age are more effective. |
|  | Lorig et al., (2001) [64] | Persons with chronic disease | Longitudinal study design post randomised controlled trial  **Intervention**: Chronic disease Self-management Program among 831 persons with heart/lung disease, stroke and arthritis aged 40 years and older.  **Outcome**: health status, health utilization and perceived self-efficacy.  **Setting**: community-based healthcare | Client-tailored Self-management Interventions are more effective in improving patient health outcomes. |
|  | Tang et al., (2006) [62] | Persons with chronic conditions | Paper on patient personal health records, definitions, benefits and strategies to overcome barriers. | Client-tailored Self-management Interventions are more effective in improving patient health outcomes. |
|  | Glazier et al., (2006) [70] | Adults with diabetes mellitus | Systematic review of 17 randomised controlled trials, controlled trials, or before-and-after studies  **Interventions**: self-management interventions (education,  **Outcome**: change health behaviour, knowledge and beliefs  **Setting**: primary healthcare | Cultural tailoring of self-management interventions contributes to improved patient health outcomes. |
|  | Schillinger et al., (2002) [71] | Adults with diabetes mellitus | Cross-sectional observational study  on the association between health literacy and diabetes outcomes among 408 persons with diabetes mellitus  **Setting**: primary healthcare | Adequate health literacy contributes to improved glycaemic control among persons with diabetes mellitus.  Self-management Interventions should be tailored to patients’ needs. |
| (1.2.b) Nurses utilize the “ask-tell-ask” (also known as Elicit-Provide-Elicit) communication technique to ensure the client receives the information required or requested. | Miller & Rollnick (2002) [51] | healthcare professionals | Book on motivational interviewing to overcome the challenges associated with behaviour change. | The ask-tell-ask approach can help healthcare providers to offer information to patients in a manner directed by the patient. |
|  | Resnicow et al., (2001) [72] | African America adults | Randomised controlled trial  **Intervention**: motivational interviewing among 14 randomly churches with African- American patients. 3 treatment conditions (comparison, self-help intervention with 1 telephone call and self-help group with 1 cue call and 3 counselling calls.  **Outcome**: fruit and vegetable intake measured with a questionnaire.  **Setting**: community-based healthcare | The motivational interview group recorded a greater increase in fruit and vegetable intake compared to the comparison or self-help group.  The tone of the interaction with the client should be non-judgement, positive and empathetic. |
|  | Miller (1983) [73] | Persons with health behaviour change needs (Problem Drinkers) | Article on motivational interviewing for behavioural change. | The patient requiring behaviour change should be helped by the healthcare provider to identify and achieve health goals through positive affirmation which improves self-efficacy and self-esteem. |
|  | Rollnick (2003) [74] | Persons requiring information/  education | Book on patient education | Patients are more likely to retain the information they wish to know |
|  | Barrier et al., (2003) [75] | Physicians and their patients | Article on improving physician-patent communication by asking “What else?” | A patient-centred approach can enable the disclosure of problems by the patient to improve communication between the physician and the patient.  The ask-tell-ask approach can be used during medical interviews. |
|  | Keller & Gregory Carrol (1994) [76] | Physicians and their patients | An article on the E4 (Engage, Empathise, Educate and Enlist) model for physician and patient communication | healthcare providers should encourage patients to set the meeting’s agenda by asking If they have anything else on their minds to tell them.  The ask-tell-ask approach can be used during medical interviews. |
|  | Lipton et al., (2008) [77] | Persons with migraine | Qualitative study among 60 patients with migraine for at least 14 years. | healthcare professionals often use narrowly focused, closed-ended questions during interactions with patients.  Dialogue between the health care provider and the accuracy of the questions posed to the patient can enhance patient assessment. |
| (1.2.c) Nurses use the communication technique “Closing the Loop”  (also known as “ teach back”) to assess a client’s understanding of information. | Miller & Rollnick (2002) [51] | healthcare professionals | Book on motivational interviewing to overcome the challenges associated with behaviour change. | healthcare providers communicating with patients should assess patients’ level of understanding of the information they have provided. |
|  | Makoul (2001) [78] | healthcare professionals | Article on the Kalamazoo consensus statement by 21 experts in communication attending the Bayer–Fetzer Conference on Physician-Patient Communication in Medical Education, North America. | healthcare providers checking for patients’ understanding is a core communication skill of information sharing with patients. |
|  | Bertakis (1977) [79] | Patients receiving care in a clinic | Two Randomised controlled trials among a total of 100 patients receiving care in a clinic  **Intervention**: asking patient to restate information provided, followed by physician feedback.  **Outcome**: Information retention  **Setting**: Clinic, United States of America | Asking a patient to repeat information received in their own words contributes to a significant increase in information retention by the patient. |
|  | Schillinger et al., (2002) [71] | Adults with diabetes mellitus | Cross-sectional observational study  on the association between health literacy and diabetes outcomes among 408 persons with diabetes mellitus  **Setting**: primary healthcare | Adequate health literacy contributes to improved glycaemic control among persons with diabetes mellitus.  Patients who are asked to repeat the information provided obtained lower average glycated haemoglobin levels compared. |
|  | Kemp et al., (2008) [80] | Patients receiving care in a clinic | Videotape stimulus technique study  Used to assess patient participants’ choice of methods for assessing understanding of medical knowledge using a physician or patient-focused approaches  **Setting**: Clinic | Patients favoured a “tell-back collaborative method” in assessing understanding of medication information over others which were physician-centred or included responding whether one understands or not. |
| (1.2.d) Nurses assist clients in using information from self-monitoring  techniques (e.g., glucose monitoring, home blood pressure monitoring) to manage their condition. | Powell & Gibson (2002) [81] | Adults (>16 years) with asthma | Systematic review of 15 randomised controlled trials  **Interventions**: asthma education  **Outcome**: admissions, doctor visits, days lost from work or school, lung function (FEV1), peak expiratory flow (PEF), use of rescue beta-agonists, courses of oral corticosteroids, symptom scores, quality of life scores  **Setting**: primary healthcare | Symptom diaries are effective in monitoring the symptoms of asthma among patients. |
|  | Toelle & Ram (2004) [6] | Adults and Children with Asthma | Systematic review of 7 Randomised controlled trials  **Intervention**: education, individualised written asthma self-management plan  **Outcomes**: medication adherence, hospitalisation, emergency department visits, oral corticosteroid use, lung function, days lost from school/work, unscheduled doctor visits and respiratory tract infections  **Setting:** primary healthcare | There is no significant difference in adherence between the group that kept a written symptom diary and the group that did not. |
|  | Lefevre et al., (2002) [82] | Adults and Children with Asthma | Systematic review of 9 randomised controlled trials  **Intervention**: use of written asthma self-management plan with or without peak flow monitoring  **Outcomes**: frequency of waiting and examination room companions, the reasons for accompaniment, the influence on the encounter, helpfulness of companion as assessed by patients and companions  **Setting:** Clinic | Majority of persons with asthma who used written action plans did not record improved health outcomes. |
|  | Gibson et al., (2007) [83] | Adults (> 16 years) with asthma | Systematic review of randomised controlled trials  **Intervention**: self-monitoring of asthma symptoms with written action plans  **Outcomes**: admissions, doctor visits, quality of life  **Setting:** primary care vs hospital-based | Adults with asthma who monitor their health status with written action plans record improved health outcomes. |
|  | Berikai et al., (2007) [84] | Adults with diabetes mellitus | Retrospective study among 155 patients receiving care between 2001 and 2004 from a diabetes health facility.  Questionnaire assessing patient’s knowledge on diabetes mellitus  **Setting:** Clinic, United States of America | Persons with Diabetes are more likely to achieve self-management targets if they have adequate knowledge about their target outcomes such as glycated haemoglobin levels. |
|  | Yang et al., (2004) [85] | Patients on warfarin | Literature analysis on home monitoring of prothrombin time at patients. | Patients who self-monitored and self- adjusted warfarin doses at home recorded target international normalized ratios (INR) compared to clients on physician-adjusted doses |
|  | Welschen et al., (2005) [86] | Patients with diabetes mellitus not on insulin | Systematic review of 6 randomised controlled trials  **Intervention**: effects of self-monitoring of blood glucose  **Outcomes**: glycemic control, quality of life and well-being, patient satisfaction  **Setting:** home-based care | Self-monitoring of blood glucose may be useful for monitoring glycaemic control among persons with diabetes mellitus. |
|  | Farmer et al., (2007) [87] | Patients with diabetes mellitus not on insulin | Randomised controlled trial.  **Intervention**: Standardised (n = 152), blood glucose self-monitoring with advice for patients to contact their doctor for interpretation of results, in addition to usual care (n=150), and blood glucose self-monitoring with additional training of patients in interpretation and application of the results to enhance motivation and maintain adherence to a healthy lifestyle (n=151).  **Outcome**: Glycated haemoglobin level measured at 12 months. | There were no statistically significant differences among the groups of persons with diabetes who self-monitor their glucose levels versus standard care. |
|  | Towfigh et al., (2008) [88] | Persons with diabetes mellitus | Systematic review and metanalysis of 9 randomised controlled trials  **Intervention**: efficacy of self-monitoring of blood glucose (SMBG) levels among patients with diabetes mellitus (DM)  **Outcomes**: glycemic control, quality of life and well-being, patient satisfaction  **Setting:** home-based care | Self-monitoring of blood glucose has a statistically significant but clinically modest effect in controlling blood glucose levels in patients with diabetes not taking insulin. |
|  | Blonde et al., (2009) [89] | Adults (≥18 years) with diabetes mellitus | Randomised controlled trial.  **Intervention**: Once-daily insulin doses adjusted using algorithm-guided patient-directed titration to achieve target plasma glucose values.  **Outcome**: Glycated haemoglobin level. | Persons with diabetes on insulin who utilize monitoring information and algorithms to adjust inulin doses record improved metabolic outcomes. |
|  | Selam & Meneghini (2009) [90] | Adults (≥18 years) with diabetes mellitus | Randomised controlled trial.  **Intervention**: 26-week, randomized, phase 4 study where adults with diabetes mellitus could adjust their insulin detemir dose every 3 days  **Outcome**: Glycated haemoglobin level. | Patient-directed modification of insulin doses is comparable to physician-directed insulin dose adjustment. |
|  | Cappuccio et al., (2004) [91] | Persons with essential hypertension | Systematic review of 18 randomised controlled trials  **Intervention**: home blood pressure monitoring  **Outcomes**: Differences in systolic, diastolic or mean blood pressure  **Setting:** primary healthcare | Home blood pressure monitoring by patient is associated with improved blood pressure control compared with clinic-only blood pressure monitoring. |
| (1.2.e) Nurses encourage clients to use monitoring methods (e.g., diaries, logs, personal health records) to monitor and track their health condition. | Cincinnati Children’s Hospital Medical Centre (2007) [92] | Children with chronic conditions | Report on the management of children with chronic conditions.  **Setting:** primary healthcare | Patients with chronic conditions can use diaries and journals for self-monitoring during self-management support. |
|  | Scherger (2005) [93] | Persons with chronic conditions | Report on the management of children with chronic conditions.  **Setting:** primary healthcare | personal health recording systems contribute to behavioural change among persons with chronic conditions. |
|  | Tang & Lansky (2005) [94] | Patient seeking healthcare | Opinion article on the management of persons with chronic conditions.  **Setting:** Clinic | Personal health records (PHRs) can facilitate collaboration between patients and healthcare providers. |
|  | Tang et al., (2003) [95] | Patient seeking healthcare | Opinion article on the management of persons with chronic conditions.  **Setting:** Clinic | Electronic medical record system can promote collaboration between healthcare providers and patients. |
|  | Wald et al., (2004) [96] | Patient seeking healthcare | Article on Longitudinal medical records used by 8700 patients of 90 Physicians | Ambulatory electronic medical care record systems can promote collaboration between healthcare providers and patients. |
|  | Newell et al., (2002) [97] | Persons at risk of cancer and cardiovascular disease | Randomised controlled trial.  **Intervention**: Administration of personal Health Record Booklets including current recommendations cancer risk and cardiovascular disease risk reduction  **Outcome**: Pap-smear tests, mammograms, and skin operations counts  **Setting:** Community, Australia | Patient’s cancer screening behaviour not increased by use of personal health record booklets |
|  | Simmons et al., (2004) [98] | Persons with poorly controlled diabetes. | Randomised controlled trial.  **Intervention**: New Zealand Diabetes Passport providing information on diabetes.  **Outcome**: Glycated haemoglobin levels  **Setting:** small town, Australia | Patient knowledge on diabetes, self-empowerment and glycaemic control not improved by keeping a diabetes passport |
|  | Tang et al., (2006) [62] | Persons with chronic conditions | Paper on patient personal health records, definitions, benefits and strategies to overcome barriers. | The capabilities and use of personal health record systems is not fully covered by current evidence. |
| **1.3 AGREE** | | | | |
| (1.3) Nurses collaborate with clients to:  • Establish goals,  • Develop action plans that enable achievement of goals: and  • Monitor progress towards goals | Bodenheimer & Handley (2009) [65] | Adolescents and adults with chronic conditions | Literature review of 8 articles on  processes of engaging patients in goal-setting.  **Setting**: primary healthcare | Collaborative goal-setting and feedback between the healthcare provider and the person with chronic condition contributed to behaviour change and improved self-efficacy for clinically significant behaviour changes. |
|  | Marks et al., (2005) [99] | Persons with chronic conditions | Literature review on the educational implications of interventions which improve self-efficacy among persons with chronic conditions. | self-management strategies that included enhancement of patients’ self-efficacy contribute to improving patients’ self-management. |
|  | Ammerman et al., (2002) [100] | Persons with nutritional health behaviour challenges | Systematic review of 33 randomised controlled trials, systematic reviews and observational studies  **Intervention**: nutrition counselling  **Outcomes**: nutrition health behaviour  **Setting:** primary healthcare | Setting goals or action planning as part of self-management contributes to improved health behaviours |
|  | Shilts et al., (2004) [101] | Persons with nutritional health problems | Systematic review of 28 randomised controlled trials, systematic reviews and observational studies  **Intervention**: goal-setting for self-management  **Outcomes**: effectiveness of goal setting for nutrition and physical activity-related change in behaviour  **Setting:** primary healthcare | Goal setting or action planning for adults can contribute to improved self-management of chronic conditions |
|  | Estabrooks et al., (2005) [102] | Adults (≥25 years) with diabetes mellitus | randomised controlled trial.  **Intervention**: selection of a behavioural goal and receiving mail and telephone support for 6 months  **Outcome**: Frequency of behavioural goal selection  **Setting:** primary healthcare, United States of America | Choosing goals for self-management and making plans to attain those goals often chose goals for behaviours |
|  | Handley et al., (2006) [103] | Clinicians and their patients seeking primary healthcare | Descriptive study  among 228 patients and 8 primary health clinicians to assess the feasibility of patients making action plans during the primary care visit; carrying out action plans and describing action plans chosen by patients.  Setting: primary healthcare, United States of America | Patient behaviour change can be promoted by integrating collaborative goal setting and action plans into routine primary care provision. |
|  | Bodenheimer & Handley (2009) [65] | Adolescents and adults with chronic diseases | Literature review of 8 articles on  processes of engaging patients in goal-setting.  **Setting**: primary healthcare | Action planning between patients and healthcare providers is considered a strategy for goal setting. |
| **1.4 ASSIST** | | | | |
| (1.4.a) Nurses who are appropriately trained use motivational interviewing with their clients to allow clients to fully participate in identifying their desired behavioural changes. | Miller & Rollnick (2002) [51] | healthcare professionals | Book on motivational interviewing to overcome the challenges associated with behaviour change. | Motivational Interviewing is a patient-centred directive approach to enhancing the intrinsic motivation of a patient to change behaviour by exploring and resolving ambivalence.  Motivational interviewing is not an easy skill to master but requires repetitive practice and feedback. |
|  | Miller & Rollnick (2009) [104] | healthcare professionals and patients undergoing counselling | Article on ten things that motivational interviewing is not. | Motivational interviewing is a complex, individualized, person-centred and collaborative method to enhance client’s motivation for change.  Favourable outcomes are more likely to be attained with the increasing use of motivational interviewing. |
|  | Rubak, Sandbaek, Lauritzen & Christensen (2005) [15] | Persons with chronic conditions | Systematic Review of 72 Randomised controlled trials  **Intervention**: effectiveness of motivational interviews  **Outcome**: body mass index, total blood cholesterol, systolic blood pressure, cigarettes per day and A1C levels  **Setting**: primary/secondary healthcare | Motivational interviewing improved body mass index, total blood cholesterol, systolic blood pressure, but not cigarettes per day and A1C levels |
|  | Lewin et al., (2001) [105] | healthcare providers | Systematic review of 17 randomised controlled trials and non-experimental studies  **Intervention**: Interventions that promote patient-centred care in clinical consultations  **Outcome**: healthcare behaviour and status, patient satisfaction  **Setting**: primary healthcare | Motivational interviewing can be used to counsel patients and families on changing negative health behaviours. |
|  | Hettema & Hendricks (2010) [106] | Persons who smoke | Meta-analytic review of 17 randomised controlled trials and non-experimental studies  **Intervention**: motivational interviewing  **Outcome**: smoking cessation  **Setting**: health-facility | Motivational interview is effective for smoking cessation.  Motivational interviewing can be combined with other treatment methods for behaviour change. |
|  | Rubak, Sandbaek, Lauritzen & Christensen (2005) [15] | Persons with chronic conditions | Systematic Review of 72 Randomised controlled trials  **Intervention**: effectiveness of motivational interviews  **Outcome**: body mass index, total blood cholesterol, systolic blood pressure, cigarettes per day and A1C levels  **Setting**: primary/secondary healthcare | Motivational interviewing outperforms traditional advice-giving for behavioural health problems.  Well-trained healthcare providers can use motivational interviewing skills in care delivery. |
| (1.4.b) Nurses teach and assist clients to use problem-solving techniques. | Funnell et al., (2008) [107] | Persons with diabetes mellitus | National standards for diabetes self-management education | Being able to solve problems is a core skill for self-management among persons with chronic conditions. |
|  | Glasgow et al., (2002) [108] | Persons with diabetes mellitus and heart failure | Article on self-management aspects of the improving chronic illness care breakthrough series  **Design:** Expert discussions in four group meetings over 13 months | Problem-solving skills are critical to self-management among persons with chronic conditions. |
|  | Glasgow et al., (2003) [12] | Persons with chronic conditions | Article on self-management support models  **Setting**: primary healthcare | Self-management support is iterative, individualised patient-centred and requires collaborative goal setting  Problem-solving skills are critical to self-management among persons with chronic conditions |
|  | Lorig & Holman (2003) [109] | Persons with chronic conditions | Article on Self-Management Education: History, Definition, Outcomes, and Mechanisms  **Setting**: primary healthcare | Problem-solving skills are critical to self-management among persons with chronic conditions |
|  | Whitlock et al., (2002) [3] | Adults with risky behaviour | Systematic review of interventions  **Intervention**: behavioural counselling interventions  **Outcome**: change in behaviour  **Setting**: primary healthcare | Problem-solving ability is a core self-management skill among persons with chronic conditions |
|  | Malouff et al., (2007) [110] | Persons with mental or physical health  problems | Systematic Review with meta-analysis of 31 Randomised controlled trials  **Intervention**: Problem-solving therapy  **Outcome**: reduction in mental and physical health problems  **Setting**: primary/secondary healthcare | Problem-solving therapy is more effective than no treatment among persons with chronic physical or mental health conditions |
|  | Bell & D’Zurilla (2009) [111] | Persons with depression | Systematic Review with meta-analysis of 21 Randomised controlled trials  **Intervention**: Problem-solving therapy  **Outcome**: depressive symptomatology  **Setting**: primary/secondary healthcare | Problem-solving therapy is more effective than no treatment among persons with chronic mental illnesses as depression. |
|  | Glasgow, Goldstein, Ockene & Pronk (2004) [21] | Persons with chronic conditions | Literature review  **Intervention**: multifaceted behaviour change support using the 5A (Assess, Advice, Agree, Assist and Arrange) approach  **Outcome**: change in health behaviour  **Setting**: primary healthcare | Sustainable multiple behavioural changes should be patient-centred, tailored, proactive, population-based, culturally proficient, multilevel, and ongoing.  The effects of the intervention on diet and self-efficacy were partially mediated through problem-solving |
|  | Hill-Briggs & Gemmell (2007) [112] | Persons with diabetes mellitus | Systematic Review of 52 quantitative and qualitative studies  **Intervention**: Problem-solving therapy  **Outcome**: problem-solving, self-management behaviors, and physiological, psychosocial and process outcomes.  **Setting**: primary healthcare | Problem-solving therapy contributes to improved health behaviours among persons with diabetes mellitus. |
| (1.4.c) Nurses are aware of community self-management programs in a variety of settings, and link clients to these programs through the provision of accurate information and relevant resources. | Cifuentes et al., (2005) [113] | Persons with unhealthy behaviours such as smoking, unhealthy diet and inactivity. | Report on 17 practice-based research network (PBRNs) projects which focused on health behaviour change among participants in 120 family medicine, internal medicine, pediatric, and nursing practices. | Self-management can be enhanced by linking clinic-based care to community-based care. |
|  | Renders et al., (2001) [114] | Persons with diabetes mellitus | Systematic Review of 41 randomised controlled trials, controlled before-after study and interrupted time series design  **Intervention**: interventions targeted at health care professionals and aimed at improving the process of care or patient outcomes for patients with diabetes  **Outcome**: problem-solving, self-management behaviours, and physiological, psychosocial and process outcomes.  **Setting**: primary care, outpatient, and community settings. | Multifaceted professional and organizational interventions can make patient reviews effective. Self-management can be enhanced by linking clinic-based care to community-based care. |
|  | Woolf et al., (2005) [18] | Persons with chronic conditions | Focused review of program records and literature  **Intervention**: 17 Prescription for Health based on 5A model – Assess, Advice, Agree, Assist, Arrange  **Outcome**: glycaemic control  **Setting**: primary healthcare | Intervention facilitated patients’ (1) identification and change of unhealthy behaviours,  (2) access to information at home (3) use self-help methods,  (4) access to intensive counselling, and (5) follow-up  Self-management can be enhanced by linking clinic-based care to the community care |
|  | Heisler (2010) [115] | Persons with diabetes mellitus | Article on models for the mobilization of peer support to enhance diabetes self-management and clinical outcomes | Nurses can collaborate with effective community self-management programs as an additional resource to promote patients’ self-management. |
|  | Green & Kreuter (1999) [116] | healthcare providers | Book on planning health promotion planning using educational and environmental approaches | The various levels of influence on health behaviour change should be considered by healthcare providers when linking patients to community resources. |
|  | Eakin et al., (2007) [117] | Persons with chronic diseases | Randomised controlled trial.  **Intervention**: 2 face-to-face, self-management support and community linkage sessions with a health educator, 3 follow-up phone calls, and 3 tailored newsletters (n = 200 participants)  **Outcome**: dietary behaviour and physical activity  **Setting:** primary healthcare, United States of America | Identifying multi-level and community supports for health behaviour change contributed to improved dietary behaviour and multilevel support for healthy lifestyles. |
|  | Deakin, McShane, Cade & Williams (2005) [8] | Adults with type 2 diabetes | Systematic Review of 11 Randomised controlled trials  **Intervention**: group-based, patient-centred training  **Outcome**: levels of glycated haemoglobin, fasting blood glucose, body weight, diabetes knowledge, need for diabetes medication  **Setting**: primary healthcare | Group-based self-management education can reduce health care costs and resource utilization, and improve patient health outcomes |
|  | National Council on Aging Center for Healthy Aging (2006) [118] | Ageing population | Paper on care and support for ageing populations. | Effective evidence-based health promotion programs offered by trained community volunteers exist and can be considered by healthcare providers to promote self-management. |
|  | Brody et al., (2005) [119] | Adults (≥60 years) with age-related macular degeneration | Randomised controlled trial.  **Intervention**: 12-hour self-management program and 12 hours of tape-recorded health lectures  **Outcome**: emotional distress, function, self-confidence, depression status  **Setting:** primary healthcare, United States of America | A month 6, the self-management support program is effective in promoting self-efficacy and preventing depression in elderly patients with sight challenges. |
|  | Fu et al., (2003) [120] | Adults with chronic conditions (≥20 years) | Randomised controlled trial.  **Intervention**: Shanghai Chronic Disease Self-Management Program (n = 954 participants)  **Outcome**: health behaviours, health status, self-efficacy, and health service utilization  **Setting:** primary healthcare, China | Participants' health behaviour, self-efficacy, and health status reduced the number of hospitalizations six months after the course. |
|  | Fu et al., (2006) [121] | Adults with chronic conditions (≥20 years) | Qualitative study  Individual semi-structured interviews among 57 participants to explore the impact of Chronic Disease Self-Management Program (CDSMP) on participants’ perception of their behaviour, health status and quality of life and how the program achieves its benefits, and to collect suggestions for future improvement and dissemination.  Setting: primary healthcare, China | Participants experienced elevated knowledge, self-management skills, positive health behaviour, increased self-confidence, health status and quality of life. |
|  | Lorig & Holman (2003) [109] | Persons with chronic conditions | Article on Self-Management Education: History, Definition, Outcomes, and Mechanisms  **Setting**: primary healthcare | Problem-solving skills are critical to self-management among persons with chronic conditions |
|  | McGowan (1994) [122] | Persons with arthritis | Study on the impact of the Arthritis Self-Management Program in Canada  **Setting**: primary healthcare | Evidence-based self-management support program was effective in providing patients with self-management and health outcomes. |
|  | Plews (2005) [123] | Persons with chronic conditions | Article describing the Expert Patient Programme developed for the management of patients with long-term conditions  **Setting**: Great Britain | Evidence-based self-management support program was effective in proving patients’ self-management and health outcomes. |
|  | Siu et al., (2007) [124] | Persons with chronic conditions | Evaluation study  **Intervention**: 6-week Chronic Disease Self-Management Program (CDSMP) among 148 participants  **Outcome:** Health status  **Setting**: primary healthcare, Hong Kong. | The self-management program improved the self-efficacy, exercise behaviour, and application of cognitive coping strategies of participants |
|  | Sobel et al., (2002) [125] | Persons with chronic conditions | Evaluation study on Chronic Disease Self-Management Program  **Intervention**: 6-week Chronic Disease Self-Management Program (CDSMP) among 15 participants delivered by volunteers.  **Setting**: Clinic, United States of America | The self-management program was cost-effective in improving the self-management and health outcome of persons with chronic conditions. |
|  | Swerissen et al., (2006) [126] | Persons with chronic conditions | Evaluation study on Chronic Disease Self-Management Program  **Intervention**: 6-week Chronic Disease Self-Management Program (CDSMP) among 320 participants.  **Outcome**: energy, exercise, symptom management, self-efficacy, general health, pain, fatigue and health distress  **Setting**: primary healthcare, Australia | The self-management program contributed to improving the self-management and health outcomes of persons with chronic conditions. |
|  | Leveille et al., (1998) [127] | Older adults (≥70 years) with chronic conditions | Randomised controlled trial.  **Intervention**: self-management and disability prevention program. (n = 201 participants)  **Outcome**: health behaviour, functioning, and healthcare utilization  **Setting:** primary and community healthcare, United States of America | Self-management support programs can include providing information about health conditions and their treatment, medication, symptom and emotional management. Other areas include psychological consequences, lifestyle behaviour change, social support, and communication. |
|  | Barlow & Ellard (2007) [128] | Persons with chronic conditions | Qualitative study  Individual semi-structured interviews among lay-led providers of the Expert Patient Programme  **Setting**: Work setting, United Kingdom | Self-management support programs contribute to improved health outcomes such as knowledge, symptoms management, communication with healthcare providers and self-efficacy in self-management. |
|  | Deakin, McShane, Cade & Williams (2005) [8] | Adults with type 2 diabetes | Systematic Review of 11 Randomised controlled trials  **Intervention**: group-based, patient-centred training  **Outcome**: levels of glycated haemoglobin, fasting blood glucose, body weight, diabetes knowledge, need for diabetes medication  **Setting**: primary healthcare | Group-based self-management education can reduce health care costs and resource utilization, and improve patient health outcomes |
|  | Gifford et al., (1998) [129] | Men with symptomatic HIV/AIDS | Randomised controlled trial.  **Intervention**: Peer-led interactive health education groups teaching self-management skills (medication use, symptoms assessment and management, communication with healthcare providers, relaxations and physical exercise) (n = 71 participants)  **Outcome**: status of symptoms, self-efficacy and health behaviours.  **Setting:** primary healthcare, United States of America | The intervention contributed to reduced symptom severity and increased self-efficacy. Fatigue, pain and psychological symptoms were not significantly improved. |
|  | Heisler (2006) [130] | Persons with chronic conditions and healthcare providers | Report on Seven models for building peer support programs to manage chronic diseases.  **Setting:** primary healthcare, United States of America | Peer support interventions which are well designed can empower persons with chronic conditions to improve the self-management of their health |
|  | Lorig et al., (1985) [131] | Persons with chronic conditions | Randomised controlled trial.  **Intervention**: 4-month Arthritis Self-Management Program providing self-help education for self-management (n = 190 participants)  **Outcomes**: knowledge of arthritis, exercise and relaxation, walking, pain, disability, locus of control, the number of visits to a physician for treatment of arthritis, and the participant’s satisfaction with care.  **Setting:** primary healthcare, United States of America | Persons with chronic conditions who received self-help education records improved knowledge, recommended behaviours, and reduced pain |
|  | Lorig et al., (1999) [132] | Persons (≥40 years) with chronic conditions | Randomised controlled trial.  **Intervention**: 6-month Chronic Disease Self-Management Program providing education on the use of symptom management techniques; nutrition management; fatigue and sleep management; use of community resources; use of medications; dealing with emotions (n = 950 participants)  **Outcomes**: health behaviour change, health status and health service utilization  **Setting:** primary/community-based healthcare, United States of America | Persons with chronic conditions in the intervention arm reported improved weekly exercise, frequency of cognitive symptom management, communication with physicians, self-reported health, health distress, fatigue, disability, and social/role activity limitations. Patient hospitalization and hospital admission days were reduced significantly. |
|  | Lorig et al., (2001) [64] | Persons with chronic disease | Longitudinal study design post-randomised controlled trial (chronic disease self-management program)  among 831 persons with heart/lung disease, stroke and arthritis aged 40 years and older.  Outcome: health status, health utilization and perceived self-efficacy.  **Setting**: community-based healthcare | Client-tailored Self-management Interventions are more effective in improving patient health outcomes. |
|  | Lorig & Holman (2003) [109] | Persons with chronic conditions | Article on self-management education: history, definition, outcomes, and mechanisms  **Setting**: primary healthcare | Problem-solving skills are critical to self-management among persons with chronic conditions |
|  | Lorig et al., (2005) [133] | Persons with chronic conditions | Evaluation study  **Intervention**: Effectiveness of the Spanish-language version of Chronic Disease Self-management Program (CDSMP) (n = 445 participants)  **Outcome**: health behaviours, health status, and self-efficacy  **Setting**: community-based healthcare, United States of America | Persons living with chronic conditions recorded improved health behaviours, health status, and self-efficacy at both 4 months and 1 year |
|  | Schreurs et al., (2003) [134] | Persons with chronic conditions | Article describing the development of a theory-driven intervention to enhance self-management.  **Intervention**: five two-hour group sessions involving 6–8 participants, and facilitated by nurses with speciality asthma, diabetes, or heart failure care (n = 83 participants)  **Outcomes**: health status, self-efficacy. | Peer support interventions can empower persons with chronic conditions to improve the management of their health |
|  | Tang & Lansky (2005) [94] | Patient seeking healthcare | Opinion article on the management of persons with chronic conditions.  **Setting:** Clinic | Personal health records (PHRs) can facilitate collaboration between patients and healthcare providers. |
|  | Siu et al., (2007) [124] | Persons with chronic conditions | Evaluation study  **Intervention**: 6-week Chronic Disease Self-Management Program (CDSMP) among 148 participants  **Outcome:** Health status  **Setting**: primary healthcare, Hong Kong. | The self-management program improved the self-efficacy, exercise behavior, and application of cognitive coping strategies of participants |
|  | Swerissen et al., (2006) [126] | Persons with chronic conditions | Evaluation study on Chronic Disease Self-Management Program  **Intervention**: 6-week Chronic Disease Self-Management Program (CDSMP) among 320 participants.  **Outcome**: energy, exercise, symptom management, self-efficacy, general health, pain, fatigue and health distress  **Setting**: primary healthcare, Australia. | The self-management program contributed to improving the self-management and health outcomes of persons with chronic conditions. |
|  | Foster et al., (2007) [135] | Persons with chronic conditions | Systematic review of 17 randomised controlled trials  **Intervention**: lay person-led self-management support programs  **Outcomes**: Health status, health behaviours, healthcare use, self-efficacy  **Setting:** primary healthcare | The self-management support program led by lay persons can contribute to improved small short- term self-efficacy, health status, management of symptom, and health behaviours such as exercising. |
|  | Kennedy et al., (2007) [136] | Persons with chronic conditions | Two-arm pragmatic randomised controlled trial design  **Intervention**: The lay-led self care support group involved 6-weekly sessions to teach self care skills (n = 629 participants)  **Outcomes**: cost effectiveness, self-efficacy, reported energy and routine health services utilisation at 6 months  **Setting:** primary healthcare, England | Lay-led self-care support groups can be effective in enhancing the self-efficacy and energy levels among patients with long-term conditions, and can be cost effective over 6 months |
| **1.5 ARRANGE** | | | | |
| (1.5) Nurses arrange regular and sustained follow-up for clients based on the client’s preference and availability (e.g., telephone, email, regular appointments). Nurses and clients discuss and agree on the data/ information that will be reviewed at each appointment | Clement (1995) [137] | Persons with diabetes mellitus | Article on self-management education among persons with diabetes mellitus. | Follow-up by healthcare providers is key to ensuring self-management among persons with chronic conditions |
|  | Kim & Oh (2003) [138] | Persons with diabetes mellitus | Randomised controlled trial.  **Intervention**: 12 week Telephone calls to participants (n = 20 participants)  **Outcomes**: Glycated haemoglobin and adherence  **Setting:** home-based healthcare, South Korea | Follow-up by healthcare providers is key to ensuring improved dietary self-management among persons with chronic conditions |
|  | Diabetes Prevention Program Research Group (2002) [139] | Persons with diabetes mellitus | Randomised controlled trial.  **Intervention**: lifestyle-modification program to achieve at least a 7 percent weight loss with a minimum of 150 minutes of physical activity per week (n = 3234 participants)  **Outcomes**: diabetes (plasma glucose), weight, frequency of physical activity  **Setting:** home-based healthcare, England | Regular follow-ups can contribute to the maintenance of lifestyle changes among persons with chronic conditions. |
|  | Perri et al., (2001) [140] | Persons with obesity | Randomised controlled trial.  **Intervention**: behavioural treatment in weekly 2-hr group sessions, with 11-14 members per group, for 20 weeks, home-based walking program (n = 103 participants)  **Outcomes**: body weight, treatment adherence  **Setting:** home-based healthcare, United States of America | Regular follow-ups can contribute to the maintenance of lifestyle changes among persons with obesity |
|  | Norris et al., (2001) [141] | Persons with diabetes mellitus | Systematic review of 72 randomised controlled trials  **Intervention**: self-management training (educational, lifestyle behaviours, coping skills)  **Outcomes**: knowledge, attitudes, and self-care skills; lifestyle behaviours, psychological outcomes, and quality of life; glycemic control; cardiovascular disease risk factors; and economic measures and health service utilization  **Setting:** primary healthcare | Regular reinforcement and follow-up on persons with chronic conditions enhanced the effectiveness of short-term educational interventions. |
|  | Norris et al., (2002) [66] | Adults with diabetes mellitus | Systematic review of 31 randomised controlled trials  **Interventions**: self-management education  **Outcome**: Glycated Haemoglobin levels  **Setting**: primary, community, home-based healthcare | Glycated haemoglobin levels reduced over time among members in the intervention group. Self-management education contributed to change in patients’ learned health behaviours. Regular follow-up is required to maintain outcomes. |
|  | Brown et al., (2005) [68] | Persons with diabetes mellitus | Comparative study of 2 self-management interventions among 216 persons with diabetes mellitus aged 35 to 70 years. | Culturally appropriate self-management education interventions contribute to improved knowledge of diabetes and improved metabolic control. Scheduled follow-up is required to sustain long-term self-management education targets. |
|  | Funnell et al., (2008) [107] | Persons with diabetes mellitus | National Standards for Diabetes Self-Management Education | Individualized follow-up plans can contribute to sustaining self-management among persons with chronic conditions (diabetes mellitus) |
| **2. INNOVATIVE DELIVERY MODELS** | | | | |
| (2.0) Nurses use a variety of innovative, creative and flexible modalities with clients when providing self-management support such as:  a) Electronic support systems b) Printed materials  c) Telephone contact  d) Face-to-face interaction  e) New and emerging modalities | Lorig et al., (2006) [142] | Persons with chronic diseases | Randomised controlled trial.  **Intervention**: internet-based chronic diseases self-management program – peer-led, bulletin board discussion and illustrated exercises (n = 958 participants)  **Outcomes**: Health status, health behaviours and communication with physicians and self-efficacy  **Setting:** internet-based healthcare, United States of America | Persons with heart, lung or Type 2 diabetes mellitus in the internet-based self-management program recorded improved health status and self-efficacy after 1 year |
|  | Glasgow et al., (2003) [143] | Persons with diabetes mellitus | Randomised controlled trial.  **Intervention**: face-to-face self-management or diet versus internet-based self-management program Diabetes Network. Peer-led support group, professionally monitored, via the internet (n = 320 participants).  **Outcomes**: health behaviour, psychosocial status and biological outcomes  **Setting:** primary healthcare, United States of America | Use of the website reduce over time however, the internet-based self-management program contributed to improved health behaviour, psychosocial status and biological outcomes |
|  | Eakin et al., (2007) [117] | Persons with chronic diseases | Randomised controlled trial.  **Intervention**: 2 face-to-face, self-management support and community linkage sessions with a health educator, 3 follow-up phone calls, and 3 tailored newsletters (n = 200 participants)  **Outcome**: dietary behaviour and physical activity  **Setting:** primary healthcare, United States of America | The use of tailored printed materials contributes to improved health outcomes for persons with chronic disease. |
|  | Kennedy et al., (2003) [144] | Persons with inflammatory bowel syndrome | Randomised controlled trial.  **Intervention**: Training of consultants in patient-centred care, providing guidebooks on ulcerative colitis and Crohn’s disease participants, and patients writing a self-management plan (n = 700 participants)  **Outcomes**: health behaviour change, health status and health service utilization  **Setting:** primary healthcare, United Kingdom | Use of tailored printed materials in combination with phone calls, face-to-face meetings and guidebooks contribute to improved the health outcomes of persons with chronic disease. |
|  | Lorig et al., (2004) [145] | Persons with Arthritis | Randomised controlled trial.  **Intervention**: mailed arthritis self-management program (SMART) care (n = 1090 participants)  **Outcomes**: self-efficacy, disability, role function  **Setting:** primary healthcare, United Kingdom | Mailed tailored print materials with questionnaires on exercise level, pain disability and other behaviours contributed to improved health outcomes among persons with chronic conditions. |
|  | Maljanian et al., (2005) [146] | Persons with diabetes mellitus | Randomised controlled trial.  **Intervention**: 12 weekly self-management support delivered through phone calls as follow-up (n = 507 participants)  **Outcomes**: adherence to American diabetes association self-care and medical care guidelines, quality of life.  **Setting:** primary healthcare, United States of America | Self-management support through telephone calls contributed to improved patients’ adherence to the American Diabetes Association's self-care and medical care guidelines. Quality of life and glycaemic control outcomes were not improved. |
|  | Coultas et al., (2005) [147] | Patients (≥45 years) with chronic obstructive pulmonary disease | Randomised controlled trial.  **Intervention**: 6 monthly face-to-face self-management program delivered by nurses trained to use the Global Initiative for Chronic Obstructive Lung Disease guidelines (n = 151 participants)  **Outcomes**: illness intrusiveness, quality of life and self-reported health-care utilization  **Setting:** primary healthcare, United Kingdom | The interventions improved patient education and self-management skills however, follow-up calls did not contribute to improved health status among persons with chronic obstructive pulmonary disease |
|  | Eakin et al., (2007) [117] | Persons with chronic diseases | Randomised controlled trial.  **Intervention**: 2 face-to-face, self-management support and community linkage sessions with a health educator, 3 follow-up phone calls, and 3 tailored newsletters (n = 200 participants)  **Outcome**: dietary behaviour and physical activity  **Setting:** primary healthcare, United States of America | Self-management support delivered face to face contributes to improved patient health outcomes. |
|  | Schillinger et al., (2008) [148] | Persons with diabetes mellitus | Randomised controlled trial.  **Intervention**: the IDEALL self-management support project delivered through automated telephone disease management and group medical visits, (n = 200 participants)  **Outcome**: participation among clinics, clinicians, and patients, patient representativeness; and engagement with self-management support among patients  **Setting:** primary healthcare, United States of America | Automated telephone disease management contributed to improved health outcomes for participants. |
| (2.1) Nurses tailor the delivery of self-management support strategies to the clients’ culture, social and economic context across settings. | Tang et al., (2005) [149] | Persons with diabetes mellitus | Longitudinal, prospective pretest and post-test design  **Intervention:** 90-minute weekly educational sessions led by diabetes nurses (n = 62)  **Outcomes**: Self-care behaviour, Self-management difficulty, psychological distress  **Setting:** community-based healthcare, United States of America | Family networks and religious communities were a strong source of social support for self-management among persons with diabetes mellitus. |
|  | Nath (2007) [150] | Persons with diabetes mellitus | Article on literacy and self-management of diabetes | To reduce the barriers experienced by patients with inadequate literacy, nurses and other healthcare providers must simplify care provision, tailor education to clients and reduce the complex nature of the health system. |
|  | McGowan (1994) [122] | Persons with arthritis | Study on the impact of the Arthritis Self-Management Program  **Setting**: primary healthcare, Canada. | Evidence-based self-management support program was effective in proving patients' self-management and health outcomes. |
|  | McGowan (2006) [151] | Persons with chronic conditions | Evaluation report of the British Columbia Chronic Disease Self-Management Program | The self-management program has demonstrated that it is effective in improving the health outcomes (health status, self-efficacy, knowledge) of persons living with chronic conditions in British Columbia. |
|  | Fu et al., (2006) [121] | Persons with chronic conditions | Qualitative Evaluation of Chronic Disease Self-Management Program  **Methods**: Qualitative data collected via interviews (n = 57)  **Setting:** community-based healthcare, China | The self-management program improved the knowledge, self-management skills, health status, health behaviour, self-efficacy and quality of life of persons with chronic conditions. |
|  | Chui et al., (2004) [152] | Persons with Rheumatoid Arthritis | Pre-test and Post-test design    **Intervention**: Evaluation study of Rheumatoid Arthritis Self-Management Programme (n = 200 participants)  **Outcome**: self-efficacy, self-management behaviour, psychological well-being and health status.  **Setting:** community-based healthcare, Hong-Kong | The self-management program improved the self-efficacy, self-management behaviour, psychological well-being and health status of persons with chronic conditions. |
|  | Siu et al., (2007) [124] | Persons with chronic conditions | Evaluation study  **Intervention**: 6-week Chronic Disease Self-Management Program (CDSMP) among 148 participants  **Outcome:** Health status  **Setting**: primary healthcare, Hong-Kong. | The self-management program improved the self-efficacy, exercise behaviour, and application of cognitive coping strategies of participants |
|  | Yip et al., (2007) [153] | Persons with osteoarthritic knee problems | randomised controlled trial.  **Intervention**: Arthritis Self-Management Programme 6 sessions of 2 hourly meetings held once a week, with 10–15 participants, led by registered nurses trained in small group leadership and basic principles of self-management (n = 149 participants)  **Outcome**: arthritic pain, fatigue level, the practice of light exercise routines, functional status, and number of unplanned arthritis-related medical consultations.  **Setting:** primary healthcare, Hong-Kong | Additional exercises as part of the self-management program improved patients’ pain/fatigue, knee range of motion and medical consultations. |
|  | Swerissen et al., (2006) [126] | Persons with chronic conditions | Evaluation study on Chronic Disease Self-Management Program  **Intervention**: 6-week Chronic Disease Self-Management Program (CDSMP) among 320 participants.  **Outcome**: energy, exercise, symptom management, self-efficacy, general health, pain, fatigue and health distress  **Setting**: primary healthcare, Australia. | The self-management program contributed to improving the self-management and health outcomes of persons with chronic conditions. |
|  | McGowan & Green (1995) [154] | Canadian Aboriginals with arthritis | Article on the strengths and weaknesses of the First Nations Arthritis Self-Management study.  **Intervention**: 6-week Chronic Disease Self-Management Program (CDSMP) among 320 participants.  **Setting**: primary healthcare, Australia. | The self-management program contributed to improving the self-management and health outcomes of participants |
|  | Fu et al., (2006) [121] | Persons with chronic conditions | Qualitative Evaluation of Chronic Disease Self-Management Program  **Methods**: Qualitative data collected via interviews (n = 57)  **Setting:** community-based healthcare, China | The self-management program improved the knowledge, self-management skills, health status, health behavior, self-efficacy and quality of life of persons with chronic conditions. |
|  | Heisler (2006) [130] | Persons with chronic conditions and healthcare providers | Report on Seven models for building peer support programs to manage chronic diseases.  **Setting:** primary healthcare, United States of America | Peer support interventions which are well designed and led by providers from diverse ethnic and cultural groups can empower persons with chronic conditions to improve their self-management. |
|  | Georges et al., (2004) [155] | Persons with health literacy challenge | Exploratory descriptive study  Assessing health literacy among vulnerable populations of African-Americans and Hispanics (n = 190)  **Setting**: community/primary healthcare, United States of America | Patients with inadequate functional health literacy have difficulty in reading, understanding, and interpreting health texts written as part of their care.  The illustrations in educational materials should represent the target population. |
|  | Registered Nurses Association of Ontario (2006) [156] | Nurses and their clients | Clinical guideline for providing client-centred care | Nurses should empower their clients and enhance their quality of care |
|  | Deakin et al., (2006) [157] | Persons with diabetes mellitus | Randomised controlled trial.  **Intervention**: X-PERT Programme: 6 two-hourly group sessions of self-management education (n = 314 participants)  **Outcome**: glycated haemoglobin, body weight, body mass index (BMI), waist circumference, total cholesterol, self-empowerment, diabetes knowledge, physical activity levels, foot care, fruit and vegetable intake, enjoyment of food and treatment satisfaction  **Setting:** community healthcare, United Kingdom | The interventions contributed to the improvement of body weight, body mass index (BMI), waist circumference, total cholesterol, self-empowerment, diabetes knowledge, physical activity levels, foot care, fruit and vegetable intake, enjoyment of food and treatment satisfaction |
|  | Norris et al., (2002) [66] | Adults with diabetes mellitus | Systematic Review of 31 Randomised controlled trials  **Interventions**: self-management education  **Outcome**: Glycated Haemoglobin levels  **Setting**: primary, community and home-based healthcare | Glycated haemoglobin levels reduced over time among the intervention group. Self-management education contributed to change in patients’ learned health behaviours. |
|  | Leveille et al., (1998) [127] | Older adults (≥70 years) with chronic conditions | Randomised controlled trial.  **Intervention**: self-management and disability prevention program. (n = 201 participants)  **Outcome**: health behaviour, functioning, and healthcare utilization  **Setting:** primary and community healthcare, United States of America | Self-management support programs can include providing information about health conditions and their treatment, medication, symptom and emotional management. Other areas include psychological consequences, lifestyle behaviour change, social support, and communication. |
|  | Pepper-Burke (2003) [158] | Persons with chronic conditions | Article describing the Chronic Disease Self-Management Workshop.  **Intervention**: Six-weeks, 21  two-hour session work-  shop on “exercising, symptom management techniques, nutrition, fatigue and sleep management, use of medications, dealing with the emotions of fear, anger, and depression, communication with others, including health professionals, problem-solving, and decision-making.  **Setting:** community-based healthcare, United States of America | The self-management program contributed to improving the self-management and health outcomes of persons with chronic conditions. |
|  | Lefort et al., (1998) [159] | Patients with chronic non-malignant pain | Randomised controlled trial.  **Intervention**: 12-hour Chronic Pain Self-Management Program (n = 102 participants)  **Outcome**: self-efficacy, self-reported level of pain and quality of life.  **Setting:** community-based healthcare, Canada | The self-management program contributed to improving the self-management and health outcomes of persons with chronic non-malignant pain. |
|  | Tang et al., (2005) [149] | Persons with diabetes mellitus | Longitudinal, prospective pretest and post test design  **Intervention:** 90-minute weekly educational sessions led by diabetes nurses (n = 62)  **Outcomes**: Self-care behavior, Self-management difficulty, psychological distress  **Setting:** community-based healthcare, United States of America | Family networks and religious communities were a strong source of social support for self-management among persons with diabetes mellitus. |
|  | Karlsson et al., (2005) [160] | Patients with congestive heart failure | Randomised controlled trial.  **Intervention**: follow-up at a nurse-based outpatient clinic (n = 208 participants)  **Outcome**: knowledge of congestive cardiac failure and self-care  **Setting:** community-based healthcare, Sweden | Female participants managed by nurses in out-client clinics recorded higher levels of self-care knowledge compared to participants receiving primary physician health care only. |
|  | Coleman et al., (2004) [161] | Adults (≥65 years) with chronic conditions | Quasi experimental study  **Intervention**: tools to facilitate cross-site communication, motivation to play more active role in care, assert preferences and guidance from a transition coach (n = 1,393 participants)  **Outcome**: post discharge hospital use rate  **Setting:** community-based healthcare, United States of America | Older clients and caregivers with communication tools and a transition coach record less hospital visits, improved communication with healthcare providers and self-confidence in self-management. |
|  | Eakin et al., (2007) [117] | Persons with chronic diseases | Randomised controlled trial.  **Intervention**: 2 face-to-face, self-management support and community linkage sessions with a health educator, 3 follow-up phone calls, and 3 tailored newsletters (n = 200 participants)  **Outcome**: dietary behavior and physical activity  **Setting:** primary healthcare, United States of America | Identifying multi-level and community supports for health behaviour change contributed to improved dietary behaviour and multilevel support for healthy lifestyles. |
|  | Glasgow et al., (2004) [162] | Post-menopausal women with diabetes mellitus | randomised controlled trial.  **Intervention**: 6-month multiple lifestyle behavior change program addressing diet, physical activity, stress management, and social support (n = 279 participants)  **Outcome**: problem solving, coping with diabetes self-care challenges  **Setting:** primary healthcare, United States of America | Assessment of the participants with the Diabetes Problem-Solving Inventory revealed that participants recorded improved dietary behaviour and community support but not physical activity |
| (2.2) Nurses facilitate a collaborative practice team approach for effective self-management support. | Bray et al., (2005) [163] | Persons with diabetes mellitus | Randomised controlled trial.  **Intervention**: 12-month case management, group visits, and electronic registry in 5 solo or small group sessions facilitated by a nurse (n = 314 participants)  **Outcome**: problem solving, coping with diabetes self-care challenges  **Setting:** primary healthcare, United States of America | Weekly visits by nurse to support self-management contributed to improved percentage of patients achieving self-management goals and documenting goals/lipid panels. |
|  | Coleman & Newton (2005) [164] | Persons with chronic conditions | Article on how to support persons with chronic illnesses to self-manage. | Healthcare providers should work closely as a team when providing self-management support to persons with chronic conditions. |
|  | Glasgow et al., (2002) [108] | Persons with diabetes mellitus and heart failure | Article on Self-Management Aspects of the Improving Chronic Illness Care Breakthrough Series  **Design:** Expert discussions in four group meetings over 13-months | Self-management support should be provided by a team of healthcare providers. |
|  | Keers et al., (2004) [165] | Persons with diabetes mellitus | An article on the effects and utilisation of a  multidisciplinary intensive education programme focusing diabetes rehabilitation | An interdisciplinary team of health care providers including nurses should is required for effective self-management support of patients. |
|  | Bodenheimer, (2003) [166] | Persons with chronic conditions | Article evaluating the effectiveness of Interventions to Improve Chronic Illness Care  **Setting:** primary healthcare | Primary care teams comprised of prepared and proactive healthcare providers from different disciplines can effectively provide self-management support to patients. |
|  | Morgan (1997) [167] | healthcare providers | Article on collaborative care planning for care on persons seeking healthcare. | Collaborative care planning promotes teamwork among healthcare professional providing care to patients. |
|  | Funnell & Anderson (2004) [168] | Persons with diabetes mellitus | Article on how to empower persons with diabetes mellitus to self-manage their chronic conditions.  **Setting:** primary healthcare, United States of America | Healthcare teams providing care to patients should be trained on the clinical, educational and behavioural facets of care. |
|  | Bodenheimer et al., (2005) [57] | Persons living with chronic conditions | Book on Strategies for healthcare providers to help people to manage their chronic conditions | Healthcare team members providing self-management support to persons with chronic conditions require training on self-management support techniques and tools to be effective.  Planned care visits by professional teams can facilitate their self-management support provision. |
|  | Mensing et al., (2002) [169] | Persons with diabetes mellitus | Article on the National Standards for Diabetes Self-Management Education  **Methods:** Task force members (Nurses, Behaviorists, Pharmacists, Physicians, Dieticians) representing organizations in diabetes care arrived at standards upon series of meeting and discussions.    **Setting:** primary healthcare, United States of America | Healthcare team members providing self-management support to persons with chronic conditions require training on self-management support techniques and tools in order to be effective. |
|  | Fisher et al., (2005) [170] | Persons with diabetes mellitus | An article on the ecological approaches to Self-Management as part of managing case s of diabetes. | Members of the health care team providing self-management support to patients should be trained on self-management techniques and tools in order to be effective. |
|  | Coleman & Newton (2005) [164] | Persons with chronic conditions | Article on how to support persons with chronic illnesses to self-manage. | Healthcare providers should work closely as a team when providing self-management support to persons with chronic conditions. Team members may include members of the community. |
|  | Jack et al., (2004) [171] | Persons with diabetes mellitus | Systematic review (n = 11 studies)  **Intervention**: Diabetes self-management education (DSME) in community gathering places  **Outcome**: knowledge, fasting blood glucose levels  **Setting:** community-based healthcare | Diabetes self-management education teams should consider including community members and resources in the design and implementation of their self-management education interventions. |
|  | Glasgow et al., (2003) [12] | Persons with chronic conditions | Article on self-management support models  **Setting**: primary healthcare | Self-management support is iterative, individualised patient-centred and requires collaborative goal setting.  The self-management support should be integrated across the clinic and community setting. |
|  | Heisler (2005) [172] | Persons with chronic conditions | Article on effective approaches that can be used by physicians’ support patients’ self-management | The provision of self-management support should be client centered and integrated across the hospital/clinic and community setting. |
|  | Glasgow, Goldstein, Ockene & Pronk (2004) [21] | Persons with chronic conditions | Literature review  **Intervention**: multifaceted behaviour change support using the 5A (Assess, Advice, Agree, Assist and Arrange) approach  **Outcome**: change in health behaviour  **Setting**: primary healthcare | Sustainable multiple behavioural changes should be patient-centred, tailored, proactive, population-based, culturally proficient, multilevel, and ongoing.  Self-management support should be client-centered and provided in an integrated form across the hospital/clinic and community setting. |
|  | Funnell & Anderson (2004) [168] | Persons with diabetes mellitus | Article on how to empower persons with diabetes mellitus to self-manage their chronic conditions.  **Setting:** primary healthcare, United States of America | The self-management support provided to patients should be client centered and integrated into the care needs of patients. |
|  | Bodenheimer (2003) [166] | Persons with chronic conditions | Article evaluating the effectiveness of Interventions to Improve Chronic Illness Care  **Setting:** primary healthcare | Primary care teams comprised of prepared and proactive healthcare providers from different disciplines can effectively provide self-management support to patients. |
|  | Jayasuriya et al., (2001) [173] | Persons with chronic conditions | Article providing an introduction to self-management of chronic diseases. | Team care can contribute to effective improvement of the health outcomes of persons with chronic conditions. |
|  | Holland et al., (2003) [174] | Persons with chronic conditions (≥36 years) | Randomised controlled trial.  **Intervention**: California Public Employees Retirement System (CalPERS) Health Matters program including strategies to prevent disability, health coaching, patient education on the self-management (n = 279 participants)  **Outcome**: wellness, health lifestyle  **Setting:** community-based healthcare, United States of America | To be effective, interdisciplinary self-management support teams should consider providing support within the communities of their clients. |
|  | Jack et al., (2004) [171] | Persons with diabetes mellitus | Systematic review (n = 11 studies)  **Intervention**: Diabetes self-management education (DSME) in community gathering places  **Outcome**: knowledge, fasting blood glucose levels  **Setting:** community-based healthcare | Diabetes self-management education teams should consider including community members and resources in the design and implementation of their self-management education interventions. |
|  | Wagner et al., (1999) [175] | Persons with chronic conditions | Survey article on 72 self-management programs in 69 organizations. | Most of the programs relied on traditional, information-oriented resources in their self-management education.  An interdisciplinary team approach should be used in providing effective self-management support to persons with chronic conditions. |
|  | Jack et al., (2004) [171] | Persons with diabetes mellitus | Systematic review (n = 11 studies)  **Intervention**: Diabetes self-management education (DSME) in community gathering places  **Outcome**: knowledge, fasting blood glucose levels  **Setting:** community-based healthcare | Diabetes self-management education teams should consider including community members and resources in the design and implementation of their self-management education interventions.  Older and vulnerable patients may be assisted by the community based care approach. |
|  | Kennedy et al., (2003) [144] | Persons with inflammatory bowel syndromne | Randomised controlled trial.  **Intervention**: Training of consultants in patient-centred care, providing guidebooks on ulcerative colitis and Crohn’s disease participants, patients writing a self-management plan (n = 700 participants)  **Outcomes**: health behaviour change, health status and health service utilization  **Setting:** primary healthcare, United Kingdom | Collaborative practice among health providers across multiple disciplines contribute to improved health outcomes of persons with chronic disease in self-management support programs. |
|  | Leveille et al., (1998) [127] | Older adults (≥70 years) with chronic conditions | Randomised controlled trial.  **Intervention**: self-management and disability prevention program. (n = 201 participants)  **Outcome**: health behaviour, functioning, and healthcare utilization  **Setting:** primary and community healthcare, United States of America | Interdisciplinary collaboration among healthcare providers contribute to improved health outcomes of persons with chronic disease in self-management support programs. |
|  | Wilson et al., (2005) [176] | Professional nurses | Qualitative descriptive, exploratory design  **Methods:** semi-structured individual interviews (n = 9 participants)  **Setting:** primary healthcare, Australia | Interprofessional collaboration among nurses, physician and allied healthcare providers is complex and requires deliberate efforts to establish it. The Interprofessional collaboration can contribute to improved patient health outcomes. |
|  | Deakin et al., (2006) [157] | Persons with diabetes mellitus | Randomised controlled trial.  **Intervention**: X-PERT Programme: 6 two-hourly group sessions of self-management education (n = 314 participants)  **Outcome**: glycated haemoglobin, body weight, body mass index (BMI), waist circumference, total cholesterol, self-empowerment, diabetes knowledge, physical activity levels, foot care, fruit and vegetable intake, enjoyment of food and treatment satisfaction  **Setting:** community healthcare, United Kingdom | The interventions contributed to improvement of body weight, body mass index (BMI), waist circumference, total cholesterol, self-empowerment, diabetes knowledge, physical activity levels, foot care, fruit and vegetable intake, enjoyment of food and treatment satisfaction.  healthcare providers offering self-management support should work in alliance with other professionals to identify approaches to successful self-management. |
|  | Loeb et al., (2003) [177] | Older adults (≥55 years) with multiple chronic conditions | Qualitative design  **Methods:** 5 semi-structured focus group (n = 37 participants)  **Setting:** hospital-based healthcare, United States of America | Care partnerships involving nurses and related health care providers can improve the life-long self-management and health of older persons with multiple chronic conditions. |
| **B. EDUCATION RECOMMENDATIONS** | | | | |
| (3.0) Nursing academic programs integrate principles of self-management support education throughout their core curriculum and in continuing education. | Redman (2004) [29] | Persons with chronic diseases | Book on evidence-based standards for self-management of chronic disease. | Nurses should assess persons with chronic health conditions for distress, anger and frustration as part of the rapport-building process.  Self-management preparation entails training persons with chronic conditions to deal with the medication, therapeutic regimen, daily life and frustrations associated with chronic conditions. |
|  | Rauscher (2006) [178] | Persons with chronic conditions | Discussion paper on self-management and self-management support  **Setting:** primary healthcare, United States of America | The education programs of health professionals should include self-management support objectives and skills. |
| (3.1) Organizations provide self-management support education through a variety of ongoing professional development opportunities to support nurses in effectively developing skills in self-management support. | College of Nurses of Ontario (2010) [179] | Professional nurses | Document on quality assurance among practicing professional nurses. | Nurses must regularly reflect on their practice and consider their strategies used in the provision of self-management support to their client. |
| **C. ORGANIZATION AND POLICY RECOMMENDATIONS** | | | | |
| (4.0) Organizations provide opportunities for nurses to take leadership roles in the provision of self-management support. | Canadian Nurses Association (2005) [180] | Professional nurses and persons with chronic conditions | Article summarizing the issues associated with chronic disease and nursing. | Professional nurses are well positioned as members of multidisciplinary healthcare teams play a vital role in prevention and management of chronic conditions. |
|  | Funnell et al., (2007) [181] | Persons with diabetes mellitus | National Standards for Diabetes Self-Management Education | Nurse case or care managers can provide important coordination for the team approach to care. |
|  | Mensing et al., (2006) [182] | Persons with diabetes mellitus | Article on the National Standards for Diabetes Self-Management Education  **Methods:** Task force members (Registered Nurses, Behaviorists, Pharmacists, Physicians, Dieticians) representing organizations in diabetes care arrived at standards upon series of meeting and discussions.    **Setting:** multiple healthcare, United States of America | Nurse case or care managers can provide important coordination for the team approach to care.  The team members’ care can be coordinated and be effectively conducted with a written care plan indicating each member’s role. |
|  | Sadur et al., (1999) [183] | Persons (16 to 75 years) with diabetes mellitus | Randomised controlled trial.  **Intervention**: 6-month cluster visit of 10-18 clients per month by an interdisciplinary team of diabetes nurse educator, a psychologist, a nutritionist, and a pharmacist providing diabetes care management on an outpatient basis (n = 314 participants)  **Outcome**: glycated haemoglobin, change in self-care practices, self-efficacy  **Setting:** community healthcare, United States of America | The intervention increased patients’ self-efficacy, glycemic control and satisfaction while reducing patients’ health care utilization. Nurse case or care managers can provide important coordination for the team approach to care of chronic conditions. |
|  | Wagner et al., (1999) [175] | Persons with chronic conditions | Survey article on 72 self-management programs in 69 organizations. | An interdisciplinary team approach should be used in providing effective self-management support to persons with chronic conditions.  Nurse case or care managers can provide important coordination for the team approach to chronic disease care.  Furthermore, a documented plan on team members’ roles can enhance communication and organize the work of team members to make them effective in patient care delivery. |
|  | Bodenheimer et al., (2005) [57] | Persons living with chronic conditions | Book on Strategies for healthcare providers to help people to manage their chronic conditions | Written plan of care informed by the patient and family’s needs that stipulates their roles as well as each care team member’s role, including the client and family can help to organize the efforts of each healthcare team member. |
|  | Jayasuriya et al., (2001) [173] | Persons with chronic conditions | Article providing an introduction to self-management of chronic diseases. | Team care can contribute to effective improvement of the health outcomes of persons with chronic conditions.  Written plan on team members; roles can organize the work of team members to make them effective in patient care delivery. |
|  | Holland et al., (2003) [174] | Persons with chronic conditions (≥36 years) | Randomised controlled trial.  **Intervention**: California Public Employees Retirement System (CalPERS) Health Matters program including strategies to prevent disability, health coaching, patient education on the self-management (n = 279 participants)  **Outcome**: wellness, health lifestyle  **Setting:** community-based healthcare, United States of America | Nurses were effective in coordinating the patient care teams to attain outcomes. |
|  | Dickey et al., (1999) [184] | Persons with chronic conditions | Literature review article on the effectiveness of office system interventions in improving behaviour-change counseling  **Setting:** primary healthcare | Teamwork can be effective in improving patient lifestyle and health behaviour. Nurses were able to easily integrated counselling sessions within busy outpatient delivery systems. |
| (4.1) Organizations integrate self-management support values and principles related to fostering client-centered care and therapeutic relationships in the delivery of care and services, through inclusion in strategic plans and organizational goals. | Woolf et al., (2005) [18] | Persons with chronic conditions | Focused review of program records and literature  **Intervention**: 17 Prescription for Health based on 5A model – Assess, Advice, Agree, Assist, Arrange  **Outcome**: behaviour change  **Setting**: primary healthcare, | Assessment of the participants with the Diabetes Problem-Solving Inventory revealed that participants recorded improved dietary behaviour and community support but not physical activity.  Clinician’s efforts at promoting clients’ health behaviour is enhanced in presence of a system to support the entire 5 A counselling sequence instead of its parts. |
|  | Glasgow et al., (2003) [12] | Persons with chronic conditions | Article on self-management support models  **Setting**: primary healthcare | Self-management support is iterative, individualised patient-centred and requires collaborative goal setting.  Champions of self-management support should be identified across organizational levels to promote self-management support provision to clients. |
| (4.2) Decision makers (Chief Executive Officers, Directors, Managers, Stakeholders) within organizations ensure adequate funding is available for self- management support initiatives such as technology to provide education to clients and nurses. |  |  | *No evidence was discussed by authors for this recommendation* |  |
| (4.3) Nursing best practice guidelines can be successfully implemented where there are adequate planning strategies, resources, organizational and administrative support and appropriate facilitation of guideline uptake among clinicians. An effective organizational plan for implementation includes:  • An assessment of organizational readiness and barriers to implementation, taking into account local circumstances.  • Involvement of all members (whether in a direct or indirect supportive function) who will contribute to the implementation process.  • Ongoing opportunities for discussion and education to reinforce the importance of best practices.  • Dedication of a qualified individual to provide the support needed for the education and implementation process.  • Ongoing opportunities for discussion and education to reinforce the importance of best practices.  • Opportunities for reflection on personal and organizational experience in implementing guidelines | Registered Nurses’ Association of Ontario (2006) [185] | Professional nurses | Guideline developed for the Implementation of clinical practice guidelines.  **Methods**: Formal consensus of stakeholders upon discussion of available evidence and theories. | A structured, systematic planning process as well as strong nursing leadership can ensure the successful implementation of best practice guideline recommendations in guidelines. |

**References of underlying evidence**

1. Pignone MP, Ammerman A, Fernandez L, Orleans CT, Pender N, Woolf S, et al. Counseling to promote a healthy diet in adults: A summary of the evidence for the U.S. Preventive Services Task Force. Am J Prev Med. 2003;24: 75–92. doi:10.1016/S0749-3797(02)00580-9

2. McTigue KM, Harris R, Hemphill B, Lux L, Sutton S, Bunton AJ, et al. Screening and Interventions for Obesity in Adults: Summary of the Evidence for the U.S. Preventive Services Task Force. Ann Intern Med. 2003;139. doi:10.7326/0003-4819-139-11-200312020-00013

3. Whitlock EP, Orleans CT, Pender N, Allan J. Evaluating primary care behavioral counseling interventions: An evidence-based approach. Am J Prev Med. 2002;22: 267–284. doi:10.1016/S0749-3797(02)00415-4

4. Fiore MC. Treating tobacco use and dependence-Clinical practice guideline. http//www Surg gov/tobacco/. 2000.

5. Gibson PG, Powell H, Wilson A, Hensley MJ, Abramson MJ, Bauman A, et al. Limited (information only) patient education programs for adults with asthma. Cochrane Database Syst Rev. 2002;2010. doi:10.1002/14651858.CD001005

6. Toelle B, Ram FS. Written individualised management plans for asthma in children and adults. Cochrane Database Syst Rev. 2004. doi:10.1002/14651858.cd002171.pub3

7. Fahey T, Schroeder K, Ebrahim S. Interventions used to improve control of blood pressure in patients with hypertension. Cochrane database Syst Rev. 2005 [cited 24 Jul 2022]. doi:10.1002/14651858.CD005182

8. Deakin TA, McShane CE, Cade JE, Williams R. Group based training for self-management strategies in people with type 2 diabetes mellitus. Cochrane Database Syst Rev. 2005. doi:10.1002/14651858.cd003417.pub2

9. Goldstein MG. Supporting self-management to improve diabetes care. R I Med J. 2004;87: 46.

10. Cohen DJ, Tallia AF, Crabtree BF, Young DM. Implementing Health Behavior Change in Primary Care: Lessons From Prescription for Health. Ann Fam Med. 2005;3: S12–S19. doi:10.1370/AFM.334

11. Dolan Mullen P, Simons-Morton DG, Ramírez G, Frankowski RF, Green LW, Mains DA. A meta-analysis of trials evaluating patient education and counseling for three groups of preventive health behaviors. Patient Educ Couns. 1997;32: 157–173. doi:10.1016/S0738-3991(97)00037-2

12. Glasgow R, Connie D, Martha F, Beck A. Implementing Practical Interventions to Support Chronic Illness Self-Management Patient Centeredness. Jt Comm Qual Saf J Novemb. 2003. Available: http://www.cochrane.org

13. Pignone MP, Gaynes BN, Rushton JL, Burchell CM, Orleans CT, Mulrow CD, et al. Screening for depression in adults: A summary of the evidence for the U.S. Preventive Services Task Force. Ann Intern Med. 2002;136: 765–776. doi:10.7326/0003-4819-136-10-200205210-00013

14. Renders CM, Valk GD, Griffin SJ, Wagner E, Eijk JT van, Assendelft WJ. Interventions to improve the management of diabetes mellitus in primary care, outpatient and community settings. Cochrane Database Syst Rev. 2000 [cited 24 Jul 2022]. doi:10.1002/14651858.CD001481

15. Sune Rubak, Annelli Sandbæk, Torsten Lauritzen, Bo Christensen. Motivational interviewing: a systematic review and meta-analysis. Br J Gen Pract. 2005;55: 305–312.

16. West DS, DiLillo V, Bursac Z, Gore SA, Greene PG. Motivational Interviewing Improves Weight Loss in Women With Type 2 Diabetes. Diabetes Care. 2007;30: 1081–1087. doi:10.2337/DC06-1966

17. Solberg LI, Brekke ML, Fazio CJ, Fowles J, Jacobsen DN, Kottke TE, et al. Lessons from Experienced Guideline Implementers: Attend to Many Factors and Use Multiple Strategies. Jt Comm J Qual Improv. 2000;26: 171–188. doi:10.1016/S1070-3241(00)26013-6

18. Woolf SH, Glasgow RE, Krist A, Bartz C, Flocke SA, Holtrop JS, et al. Putting It Together: Finding Success in Behavior Change Through Integration of Services. Ann Fam Med. 2005;3: S20–S27. doi:10.1370/AFM.367

19. Bodenheimer T, Laing BY. The Teamlet Model of Primary Care. Ann Fam Med. 2007;5: 457–461. doi:10.1370/AFM.731

20. Goldstein MG, Whitlock EP, DePue J. Multiple behavioral risk factor interventions in primary care: Summary of research evidence. Am J Prev Med. 2004;27: 61–79. doi:10.1016/j.amepre.2004.04.023

21. Glasgow RE, Goldstein MG, Ockene JK, Pronk NP. Translating what we have learned into practice: Principles and hypotheses for interventions addressing multiple behaviors in primary care. Am J Prev Med. 2004;27: 88–101. doi:10.1016/j.amepre.2004.04.019

22. Canadian Nurses Association. Canadian Registered Nurse Exam: Competencies (June 2010-May 2015). 2010. Available: http://www.cna-aiic.ca/CNA/nursing/rnexam/competencies/default_e.aspx

23. College of Nurses of Ontario. Practice Standard: Therapeutic Nurse –Client Relationship. 2006. Available: www.cno.org

24. O’Connor, G.T; Gaylor, M.S.; Nelson EC. Health counseling: Building patient rapport. Physician Assist. 1985;9: 154–155.

25. Mejo SL. Communication as it Affects the Therapeutic Alliance. J Am Acad Nurse Pract. 1989;1: 20–23. doi:10.1111/j.1745-7599.1989.tb00731.x

26. Paley G, Lawton D. Evidence-based practice: Accounting for the importance of the therapeutic relationship in UK National Health Service therapy provision. Couns Psychother Res. 2001;1: 12–17. doi:10.1080/14733140112331385198

27. Stewart MA. Effective physician-patient communication and health outcomes: a review. C Can Med Assoc J. 1995;152: 1423. Available: /pmc/articles/PMC1337906/?report=abstract

28. Registered Nurses Association of Ontario. Establishing therapeutic relationships. Journal of Pediatric Oncology Nursing. Toronto, Canada; 2006. doi:10.1177/104345429100800101

29. Redman BK. Patient Self-management of Chronic Disease: The Health Care Provider’s Challenge. Jones and Bartlett Publishers; 2004. Available: https://books.google.com.gh/books?id=6uPrQ-65nvsC

30. Skovlund SE, Peyrot M, Panel on behalf of the DIA. The Diabetes Attitudes, Wishes, and Needs (DAWN) Program: A New Approach to Improving Outcomes of Diabetes Care. Diabetes Spectr. 2005;18: 136–142. doi:10.2337/DIASPECT.18.3.136

31. Peyrot M, Rubin RR, Lauritzen T, Snoek FJ, Matthews DR, Skovlund SE. Psychosocial problems and barriers to improved diabetes management: results of the Cross-National Diabetes Attitudes, Wishes and Needs (DAWN) Study. Diabet Med. 2005;22: 1379–1385. doi:10.1111/J.1464-5491.2005.01644.X

32. Chapman A, Gratz K. The borderline personality disorder survival guide: Everything you need to know about living with BPD. New Harbinger Publications; 2007.

33. Moussavi S, Chatterji S, Verdes E, Tandon A, Patel V, Ustun B. Depression, chronic diseases, and decrements in health: results from the World Health Surveys. Lancet. 2007;370: 851–858. doi:10.1016/S0140-6736(07)61415-9

34. Anderson RJ, Freedland KE, Clouse RE, Lustman PJ. The Prevalence of Comorbid Depression in Adults With Diabetes: A meta-analysis. Diabetes Care. 2001;24: 1069–1078. doi:10.2337/DIACARE.24.6.1069

35. Taveira TH, Pirraglia PA, Cohen LB, Wu WC. Efficacy of a Pharmacist-Led Cardiovascular Risk Reduction Clinic for Diabetic Patients With and Without Mental Health Conditions. Prev Cardiol. 2008;11: 195–200. doi:10.1111/J.1751-7141.2008.00008.X

36. Cleveland Clinic. Chronic Illness and Depression. 2010 [cited 10 Aug 2022]. Available: https://my.clevelandclinic.org/health/articles/9288-chronic-illness-and-depression

37. Unützer J, Patrick DL, Simon G, Grembowski D, Walker E, Rutter C, et al. Depressive Symptoms and the Cost of Health Services in HMO Patients Aged 65 Years and Older: A 4-Year Prospective Study. JAMA. 1997;277: 1618–1623. doi:10.1001/JAMA.1997.03540440052032

38. Egede LE, Nietert PJ, Zheng D. Depression and All-Cause and Coronary Heart Disease Mortality Among Adults With and Without Diabetes. Diabetes Care. 2005;28: 1339–1345. doi:10.2337/DIACARE.28.6.1339

39. Bodenheimer T, Lorig K, Holman H, Grumbach K. Patient self-management of chronic disease in primary care. J Am Med Assoc. 2002;288: 2469–2475. doi:10.1001/jama.288.19.2469

40. Park H, Hong Y, Lee H, Ha E, Sung Y. Individuals with type 2 diabetes and depressive symptoms exhibited lower adherence with self-care. J Clin Epidemiol. 2004;57: 978–984. doi:10.1016/J.JCLINEPI.2004.01.015

41. Lerman I, Lozano L, Villa AR, Hernández-Jiménez S, Weinger K, Caballero AE, et al. Psychosocial factors associated with poor diabetes self-care management in a specialized Center in Mexico City. Biomed Pharmacother. 2004;58: 566–570. doi:10.1016/J.BIOPHA.2004.09.003

42. Katon W, Ciechanowski P. Impact of major depression on chronic medical illness. J Psychosom Res. 2002;53: 859–863. doi:10.1016/S0022-3999(02)00313-6

43. DiMatteo MR, Lepper HS, Croghan TW. Depression Is a Risk Factor for Noncompliance With Medical Treatment: Meta-analysis of the Effects of Anxiety and Depression on Patient Adherence. Arch Intern Med. 2000;160: 2101–2107. doi:10.1001/ARCHINTE.160.14.2101

44. Schmitz N, Wang JL, Malla A, Lesage A. Joint effect of depression and chronic conditions on disability: Results from a population-based study. Psychosom Med. 2007;69: 332–338. doi:10.1097/PSY.0B013E31804259E0

45. Kroenke K, Spitzer RL, Williams JBW. The Patient Health Questionnaire-2: validity of a two-item depression screener. Med Care. 2003; 1284–1292.

46. Epstein RM, Mauksch L, Carroll J, Jaen CR. Have you really addressed your patient’s concerns? Fam Pract Manag. 2008;15: 35.

47. Little P, Everitt H, Williamson I, Warner G, Moore M, Gould C, et al. Preferences of patients for patient centred approach to consultation in primary care: observational study. BMJ. 2001;322: 468. doi:10.1136/BMJ.322.7284.468

48. Middleton JF, McKinley RK, Gillies CL. Effect of patient completed agenda forms and doctors’ education about the agenda on the outcome of consultations: randomised controlled trial. BMJ. 2006;332: 1238–1242. doi:10.1136/BMJ.38841.444861.7C

49. Winefield HR, Murrell TGC, Clifford J V., Farmer EA. The usefulness of distinguishing different types of general practice consultation, or are needed skills always the same? Fam Pract. 1995;12: 402–407. doi:10.1093/FAMPRA/12.4.402

50. Hornberger J, Thom D, MaCurdy T. Effects of a self-administered previsit questionnaire to enhance awareness of patient’s concerns in primary care. J Gen Intern Med 1997 1210. 1997;12: 597–606. doi:10.1046/J.1525-1497.1997.07119.X

51. Miller WR, Rollnick S. Motivational Interviewing, Second Edition: Preparing People for Change. 2nd ed. Guilford Publications; 2002. Available: https://books.google.com.gh/books?id=p7TpwAEACAAJ

52. Edwards L, Jones H, Bleton A. The Canadian experience in the development of a continuing education program for diabetes educators based on the transtheoretical model of behavior change. Diabetes Spectr. 1999;12: 157.

53. Jones H, Edwards L, Vallis TM, Ruggiero L, Rossi SR, Rossi JS, et al. Changes in Diabetes Self-Care Behaviors Make a Difference in Glycemic ControlThe Diabetes Stages of Change (DiSC) study. Diabetes Care. 2003;26: 732–737. doi:10.2337/DIACARE.26.3.732

54. Prochaska JO, DiClemente CC, John C. In Search of How People Change: Applications to Addictive Behaviors. Am Psychol. 1992;47: 1102–1114.

55. Keller VK. Choices and changes: a new model for influencing patient health behavior. J Clin Outcomes Manag. 1997;4: 33–36.

56. Rollnick S, Mason P, Butler C. Health behavior change: A guide for practitioners. Churchill livingstone Edinburgh, UK; 2001.

57. Bodenheimer T, MacGregor K, Sharifi C. Helping patients manage their chronic conditions. California HealthCare Foundation; 2005.

58. Research and Development Corporation. Health risk appraisals and medicare. Evidence report and evidence-based recommendations. Baltimore: US Department of Health and Human Services; 2003.

59. Research and Development Corporation. Health risk appraisals and medicare. Evidence report and evidence-based recommendations. US Department of Health and Human Services; 2000.

60. Hibbard JH, Mahoney ER, Stock R, Tusler M. Do Increases in Patient Activation Result in Improved Self-Management Behaviors? Health Serv Res. 2007;42: 1443–1463. doi:10.1111/J.1475-6773.2006.00669.X

61. Anderson RM, Funnell MM. Patient empowerment: reflections on the challenge of fostering the adoption of a new paradigm. Patient Educ Couns. 2005;57: 153–157. doi:10.1016/j.pec.2004.05.008

62. Tang PC, Ash JS, Bates DW, Overhage JM, Sands DZ. Personal health records: Definitions, benefits, and strategies for overcoming barriers to adoption. J Am Med Informatics Assoc. 2006;13: 121–126. doi:10.1197/JAMIA.M2025/2/JAMIAM2025.F01.JPEG

63. Krichbaum K, Aarestad V, Buethe M. Exploring the Connection Between Self-Efficacy and Effective Diabetes Self-f Management. Diabetes Educ. 2003;29: 653–662. doi:10.1177/014572170302900411

64. Lorig KR, Ritter P, Stewart AL, Sobel DS, Brown Jr BW, Bandura A, et al. Chronic disease self-management program: 2-year health status and health care utilization outcomes. Med Care. 2001; 1217–1223.

65. Bodenheimer T, Handley MA. Goal-setting for behavior change in primary care: An exploration and status report. Patient Educ Couns. 2009;76: 174–180. doi:10.1016/j.pec.2009.06.001

66. Norris SL, Lau J, Smith SJ, Schmid CH, Engelgau MM. Self-Management Education for Adults With Type 2 DiabetesA meta-analysis of the effect on glycemic control. Diabetes Care. 2002;25: 1159–1171. doi:10.2337/DIACARE.25.7.1159

67. Barlow J, Wright C, Sheasby J, Turner A, Hainsworth J. Self-management approaches for people with chronic conditions: A review. Patient Educ Couns. 2002;48: 177–187. doi:10.1016/S0738-3991(02)00032-0

68. Brown SA, Blozis SA, Kouzekanani K, Garcia AA, Winchell M, Hanis CL. Dosage Effects of Diabetes Self-Management Education for Mexican AmericansThe Starr County Border Health Initiative. Diabetes Care. 2005;28: 527–532. doi:10.2337/DIACARE.28.3.527

69. Sarkisian CA, Brown AF, Norris KC, Wintz RL, Managione CM. A Systematic Review of Diabetes Self-Care Interventions for Older, African American, or Latino Adults. Diabetes Educ. 2003;29: 467–479. doi:10.1177/014572170302900311

70. Glazier RH, Bajcar J, Kennie NR, Willson K. A Systematic Review of Interventions to Improve Diabetes Care in Socially Disadvantaged Populations. Diabetes Care. 2006;29: 1675–1688. doi:10.2337/DC05-1942

71. Schillinger D, Grumbach K, Piette J, Wang F, Osmond D, Daher C, et al. Association of Health Literacy With Diabetes Outcomes. JAMA. 2002;288: 475–482. doi:10.1001/JAMA.288.4.475

72. Resnicow K, Jackson A, Wang T, De AK, McCarty F, Dudley WN, et al. A Motivational Interviewing Intervention to Increase Fruit and Vegetable Intake Through Black Churches: Results of the Eat for Life Trial. https://doi.org/102105/AJPH91101686. 2001;91: 1686–1693. doi:10.2105/AJPH.91.10.1686

73. Miller WR. Motivational Interviewing with Problem Drinkers. Behav Psychother. 1983;11: 147–172. doi:10.1017/S0141347300006583

74. Rollnick S. Motivational interviewing: Preparing people for change. 2nd ed. London: Guilford Press; 2003.

75. Barrier PA, Li JTC, Jensen NM. Two words to improve physician-patient communication: What else? Mayo Clin Proc. 2003;78: 211–214. doi:10.4065/78.2.211

76. Keller VF, Gregory Carroll J. A new model for physician-patient communication. Patient Educ Couns. 1994;23: 131–140. doi:10.1016/0738-3991(94)90051-5

77. Lipton RB, Hahn SR, Cady RK, Brandes JL, Simons SE, Bain PA, et al. In-office Discussions of Migraine: Results from the American Migraine Communication Study. J Gen Intern Med. 2008;23: 1145. doi:10.1007/S11606-008-0591-3

78. Makoul G. Essential Elements of Communication in Medical Encounters. Acad Med. 2001;76: 390–393. doi:10.1097/00001888-200104000-00021

79. Bertakis KD. The communication of information from physician to patient: a method for increasing patient retention and satisfaction. J Fam Pract. 1977;5: 217–222.

80. Kemp EC, Floyd MR, McCord-Duncan E, Lang F. Patients Prefer the Method of “Tell Back- Collaborative Inquiry” to Assess Understanding of Medical Information. J Am Board Fam Med. 2008;21: 24–30. doi:10.3122/jabfm.2008.01.070093

81. Powell H, Gibson PG. Options for self‐management education for adults with asthma. Cochrane Database Syst Rev. 2002.

82. Lefevre F, Piper M, Weiss K, Mark D, Clark N, Aronson N. Do written action plans improve patient outcomes in asthma? An evidence-based analysis. J Fam Pract. 2002;51: 842–848.

83. Gibson PG, Coughlan J, Wilson AJ, Abramson M, Haywood P, Bauman A, et al. Self-management education and regular practitioner review for adults with asthma. Cochrane Database Syst Rev. 2007;1.

84. Berikai P, Meyer PM, Kazlauskaite R, Savoy B, Kozik K, Fogelfeld L. Gain in patients’ knowledge of diabetes management targets is associated with better glycemic control. Diabetes Care. 2007;30: 1587–1589. doi:10.2337/dc06-2026

85. Yang DT, Robetorye RS, Rodgers GM. Home prothrombin time monitoring: A literature analysis. Am J Hematol. 2004;77: 177–186. doi:10.1002/ajh.20161

86. Welschen LMC, Bloemendal E, Nijpels G, Dekker JM, Heine RJ, Stalman WAB, et al. Self-monitoring of blood glucose in patients with type 2 diabetes who are not using insulin: a systematic review. Diabetes Care. 2005;28: 1510–1517. doi:10.2337/diacare.28.6.1510

87. Farmer A, Wade A, Goyder E, Yudkin P, French D, Craven A, et al. Impact of self monitoring of blood glucose in the management of patients with non-insulin treated diabetes: Open parallel group randomised trial. Br Med J. 2007;335: 132–136. doi:10.1136/bmj.39247.447431.BE

88. Towfigh A, Romanova M, Weinreb JE, Munjas B, Suttorp MJ, Zhou A, et al. Self-monitoring of blood glucose levels in patients with type 2 diabetes mellitus not taking insulin: A meta-analysis. Am J Manag Care. 2008;14: 468–475.

89. Blonde L, Merilainen M, Karwe V, Raskin P. Patient-directed titration for achieving glycaemic goals using a once-daily basal insulin analogue: An assessment of two different fasting plasma glucose targets - The TITRATE TM study. Diabetes, Obes Metab. 2009;11: 623–631. doi:10.1111/j.1463-1326.2009.01060.x

90. Selam J-L, Meneghini LF. Basal-bolus therapy with insulin detemir using the 303 algorithm in the US PREDICTIVE 303 trial. Adv Ther. 2009;26: 194–207.

91. Cappuccio FP, Kerry SM, Forbes L, Donald A. Blood pressure control by home monitoring: meta-analysis of randomised trials. BMJ Br Med J. 2004;329: 145. doi:10.1136/BMJ.38121.684410.AE

92. Cincinnati Children’s Hospital Medical Centre. Evidence-based care guideline for Chronic Care: Self-Management. 2007.

93. Scherger JE. Primary care needs a new model of office practice. BMJ. 2005;330: E358–E359. doi:10.1136/BMJ.330.7504.E358

94. Tang PC, Lansky D. The missing link: Bridging the patient-provider health information gap. Health Aff. 2005;24: 1290–1295. doi:10.1377/hlthaff.24.5.1290

95. Tang PC, Black W, Buchanan J, Young CY, Hooper D, Lane SR, et al. PAMFOnline: Integrating EHealth with an Electronic Medical Record System. AMIA Annu Symp Proc. 2003;2003: 644. Available: /pmc/articles/PMC1479999/

96. Wald JS, Middleton B, Bloom A, Walmsley D, Gleason M, Nelson E, et al. A patient-controlled journal for an electronic medical record: issues and challenges. Medinfo. MEDINFO. 2004. pp. 1166–1170.

97. Newell SA, Sanson-Fisher RW, Girgis A, Davey HM. Can personal health record booklets improve cancer screening behaviors? Am J Prev Med. 2002;22: 15–22. doi:10.1016/S0749-3797(01)00404-4

98. Simmons D, Gamble GD, Foote S, Cole DR, Coster G. The New Zealand Diabetes Passport Study: a randomized controlled trial of the impact of a diabetes passport on risk factors for diabetes-related complications. Diabet Med. 2004;21: 214–217. doi:10.1111/J.1464-5491.2004.01047.X

99. Marks R, Allegrante JP, Lorig K. A review and synthesis of research evidence for self-efficacy-enhancing interventions for reducing chronic disability: implications for health education practice (part I). Health Promot Pract. 2005;6: 37–43. doi:10.1177/1524839904266790

100. Ammerman A, Pignone M, Fernandez L, Lohr K, Jacob A., Nester C, et al. Counseling to promote a healthy diet. Systematic Evidence Review. 2002.

101. Shilts MK, Horowitz M, Townsend MS. Goal setting as a strategy for dietary and physical activity behavior change: A review of the literature. Am J Heal Promot. 2004;19: 81–93. doi:10.4278/0890-1171-19.2.81

102. Estabrooks PA, Nelson CC, Xu S, King D, Bayliss EA, Gaglio B, et al. The frequency and behavioral outcomes of goal choices in the self-management of diabetes. Diabetes Educ. 2005;31: 391–400. doi:10.1177/0145721705276578

103. Handley M, MacGregor K, Schillinger D, Sharifi C, Wong S, Bodenheimer T. Using Action Plans to Help Primary Care Patients Adopt Healthy Behaviors: A Descriptive Study. J Am Board Fam Med. 2006;19: 224–231. doi:10.3122/JABFM.19.3.224

104. Miller WR, Rollnick S. Ten Things that Motivational Interviewing Is Not. Behav Cogn Psychother. 2009;37: 129–140. doi:10.1017/S1352465809005128

105. Lewin S, Skea Z, Entwistle VA, Zwarenstein M, Dick J. Interventions for providers to promote a patient‐centred approach in clinical consultations. Lewin S, editor. Cochrane Database Syst Rev. 2001 [cited 17 Aug 2022]. doi:10.1002/14651858.CD003267

106. Hettema JE, Hendricks PS. Motivational interviewing for smoking cessation: a meta-analytic review. J Consult Clin Psychol. 2010;78: 868–884. doi:10.1037/a0021498

107. Funnell MM, Brown TL, Childs BP, Haas LB, Hosey GM, Jensen B, et al. National standards for diabetes self-management education. Diabetes Care. 2008;33: S89–S96. doi:10.2337/dc10-S089

108. Glasgow R, Funnell MM, Bonomi AE, Davis C, Beckham V, Wagner EH. Self-management aspects of the improving chronic illness care breakthrough series: Implementation with diabetes and heart failure teams. Ann Behav Med. 2002;24: 80–87. doi:10.1207/S15324796ABM2402_04

109. Lorig KR, Holman HR. Self-management education: History, definition, outcomes, and mechanisms. Ann Behav Med. 2003;26: 1–7. doi:10.1207/S15324796ABM2601_01

110. Malouff JM, Thorsteinsson EB, Schutte NS. The efficacy of problem solving therapy in reducing mental and physical health problems: A meta-analysis. Clin Psychol Rev. 2007;27: 46–57. doi:10.1016/J.CPR.2005.12.005

111. Bell AC, D’Zurilla TJ. Problem-solving therapy for depression: A meta-analysis. Clin Psychol Rev. 2009;29: 348–353. doi:10.1016/J.CPR.2009.02.003

112. Hill-Briggs F, Gemmell L. Problem solving in diabetes self-management and control: A systematic review of the literature. Diabetes Educ. 2007;33: 1032–1050. doi:10.1177/0145721707308412

113. Cifuentes M, Fernald DH, Green LA, Niebauer LJ, Crabtree BF, Stange KC, et al. Prescription for Health: Changing Primary Care Practice to Foster Healthy Behaviors. Ann Fam Med. 2005;3: S4–S11. doi:10.1370/AFM.378

114. Renders C, Valk GD, Griffin SJ, Wagner EH, Van Eijk JTM, Assendelft WJJ. Interventions to Improve the Management of Diabetes in Primary Care, Outpatient, and Community Settings: A systematic review. Diabetes Care. 2001;24: 1821–1833. doi:10.2337/DIACARE.24.10.1821

115. Heisler M. Different models to mobilize peer support to improve diabetes self-management and clinical outcomes: evidence, logistics, evaluation considerations and needs for future research. Fam Pract. 2010;27: i23–i32. doi:10.1093/FAMPRA/CMP003

116. Green L, Kreuter M. Health promotion planning: an educational approach and environmental approach. Mayfield Publishing Company; 1999.

117. Eakin EG, Bull SS, Riley KM, Reeves MM, McLaughlin P, Gutierrez S. Resources for Health: A Primary-Care-Based Diet and Physical Activity Intervention Targeting Urban Latinos With Multiple Chronic Conditions. Heal Psychol. 2007;26: 392–400. doi:10.1037/0278-6133.26.4.392

118. National Council on Aging Center for Healthy Aging. Using the evidence base to promote healthy aging. Issue Brief No. 1 (revised). 2006.

119. Brody BL, Roch-Levecq AC, Thomas RG, Kaplan RM, Brown SI. Self-management of Age-related Macular Degeneration at the 6-Month Follow-up: A Randomized Controlled Trial. Arch Ophthalmol. 2005;123: 46–53. doi:10.1001/ARCHOPHT.123.1.46

120. Fu D, Fu H, McGowan P, Shen Y, Zhu L, Yang H, et al. Implementation and quantitative evaluation of chronic disease self-management programme in Shanghai, China: randomized controlled trial. Bull World Health Organ. 2003;81: 174–182. doi:10.1590/S0042-96862003000300007

121. Fu D, Ding Y, McGowan P, Fu H. Qualitative evaluation of Chronic Disease Self Management Program (CDSMP) in Shanghai. Patient Educ Couns. 2006;61: 389–396. doi:10.1016/j.pec.2005.05.002

122. McGowan P. The impact of the Arthritis Self-Management Program in Canada. Toronto, Canada; 1994.

123. Plews C. Expert Patient Programme: managing patients with long-term conditions. Br J Nurs. 2005;14: 1086–1089.

124. Siu AMH, Chan CCH, Poon PKK, Chui DYY, Chan SCC. Evaluation of the chronic disease self-management program in a Chinese population. Patient Educ Couns. 2007;65: 42–50. doi:10.1016/j.pec.2006.04.013

125. Sobel DS, Lorig KR, Hobbs M. Chronic Disease Self-Management Program: From Development to Dissemination. Perm J. 2002;6: 15. Available: /pmc/articles/PMC6220632/

126. Swerissen H, Belfrage J, Weeks A, Jordan L, Walker C, Furler J, et al. A randomised control trial of a self-management program for people with a chronic illness from Vietnamese, Chinese, Italian and Greek backgrounds. Patient Educ Couns. 2006;64: 360–368. doi:10.1016/j.pec.2006.04.003

127. Leveille SG, Wagner EH, Davis C, Grothaus L, Wallace J, LoGerfo M, et al. Preventing Disability and Managing Chronic Illness in Frail Older Adults: A Randomized Trial of a Community-Based Partnership with Primary Care. J Am Geriatr Soc. 1998;46: 1191–1198. doi:10.1111/J.1532-5415.1998.TB04533.X

128. Barlow J, Ellard DR. Implementation of a Self-management Programme for People with Long-Term Medical Conditions in a Workplace Setting. J Appl Rehabil Couns. 2007;38: 24–34. doi:10.1891/0047-2220.38.2.24

129. Gifford AL, Laurent DD, Gonzales VM, Chesney MA, Lorig KR. Pilot randomized trial of education to improve self-management skills of men with symptomatic HIV/AIDS. J Acquir immune Defic Syndr Hum retrovirology Off Publ Int Retrovirology Assoc. 1998;18: 136–144.

130. Heisler M. Building peer support programs to manage chronic disease: Seven models for success. Oakland, Carlifonia; 2006.

131. Lorig K, Lubeck D, Kraines RG, Seleznick M, Holman HR. Outcomes of self-help education for patients with arthritis. Arthritis Rheum. 1985;28: 680–685. doi:10.1002/ART.1780280612

132. Lorig K, Sobel DS, Stewart AL, Brown Jr BW, Bandura A, Ritter P, et al. Evidence suggesting that a chronic disease self-management program can improve health status while reducing hospitalization: a randomized trial. Med Care. 1999; 5–14.

133. Lorig KR, Ritter PL, Jacquez A. Outcomes of border health Spanish/English Chronic Disease Self-management Programs. Diabetes Educ. 2005;31: 401–409. doi:10.1177/0145721705276574

134. Schreurs KMG, Colland VT, Kuijer RG, De Ridder DTD, Van Elderen T. Development, content, and process evaluation of a short self-management intervention in patients with chronic diseases requiring self-care behaviours. Patient Educ Couns. 2003;51: 133–141. doi:10.1016/S0738-3991(02)00197-0

135. Foster G, Taylor SJC, Eldridge SE, Ramsay J, Griffiths CJ. Self-management education programmes by lay leaders for people with chronic conditions. Cochrane Database of Systematic Reviews. 2007. doi:10.1002/14651858.CD005108.pub2

136. Kennedy A, Reeves D, Bower P, Lee V, Middleton E, Richardson G, et al. The effectiveness and cost effectiveness of a national lay-led self care support programme for patients with long-term conditions: a pragmatic randomised controlled trial. J Epidemiol Community Heal. 2007;61: 254–261. doi:10.1136/JECH.2006.053538

137. Clement S. Diabetes Self-Management Education. Diabetes Care. 1995;18: 1204–1214. doi:10.2337/DIACARE.18.8.1204

138. Kim HS, Oh JA. Adherence to diabetes control recommendations: impact of nurse telephone calls. J Adv Nurs. 2003;44: 256–261. doi:10.1046/J.1365-2648.2003.02800.X

139. Diabetes Prevention Program Research Group. Reduction in the Incidence of Type 2 Diabetes with Lifestyle Intervention or Metformin. https://doi.org/101056/NEJMoa012512. 2002;346: 393–403. doi:10.1056/NEJMOA012512

140. Perri MG, Nezu AM, McKelvey WF, Shermer RL, Renjilian DA, Viegener BJ. Relapse prevention training and problem-solving therapy in the long-term management of obesity. J Consult Clin Psychol. 2001;69: 722–726. doi:10.1037/0022-006X.69.4.722

141. Norris SL, Engelgau MM, Narayan KMV. Effectiveness of Self-Management Training in Type 2 Diabetes: A systematic review of randomized controlled trials. Diabetes Care. 2001;24: 561–587. doi:10.2337/DIACARE.24.3.561

142. Lorig KR, Ritter PL, Laurent DD, Plant K. Internet-Based Chronic Disease Self-Management. 2006;44: 964–971.

143. Glasgow R, Boles SM, Ph D, Mckay HG, Ph D, Feil EG, et al. The D-Net diabetes self-management program : long-term implementation , outcomes , and generalization results. 2003;36: 410–419. doi:10.1016/S0091-7435(02)00056-7

144. Kennedy A, Nelson E, Reeves D, Richardson G, Roberts C, Robinson A, et al. A randomised controlled trial to assess the impact of a package comprising a patient-orientated, evidence-based self-help guidebook and patient-centred consultations on disease management and satisfaction in inflammatory bowel disease. Health Technol Assess (Rockv). 2003;7. doi:10.3310/HTA7280

145. Lorig K, Ritter P, Laurent D, Fries J. Long-Term Randomized Controlled Trials of Tailored-Print and Small-Group Arthritis Self-Management Interventions. 2004;42: 346–354. doi:10.1097/01.mlr.0000118709.74348.65

146. Maljanian R, Grey N, Staff I, Conroy L. Intensive Telephone Follow-Up to a Hospital-Based Disease Management Model for Patients with Diabetes Mellitus. http://www.liebertpub.com/dis. 2005;8: 15–25. doi:10.1089/DIS.2005.8.15

147. Coultas D, Frederick J, Barnett B, Singh G, Wludyka P. A randomized trial of two types of nurse-assisted home care for patients with COPD. Chest. 2005;128: 2017–2024. doi:10.1378/chest.128.4.2017

148. Schillinger D, Hammer H, Wang F, Palacios J, McLean I, Tang A, et al. Seeing in 3-D: Examining the reach of diabetes self-management support strategies in a public health care system. Heal Educ Behav. 2008;35: 664–682. doi:10.1177/1090198106296772

149. Tang T, Gillard M Lou, Funnell MM, Nwankwo R, Parker E, Spurlock D, et al. Developing a new generation of ongoing diabetes self-management support interventions: A preliminary report. Diabetes Educ. 2005;31: 91–97. doi:10.1177/0145721704273231

150. Nath C. Literacy and diabetes self-management. Am J Nurs. 2007;107: 43–49. doi:10.1097/01.NAJ.0000277829.28043.93

151. McGowan P. The Chronic Disease Self-Management Program – program evaluation British Columbia 2003 – 2006. 2006.

152. Chui DYY, Lau JSK, Yau ITY. An outcome evaluation study of the rheumatoid arthritis self-management programme in Hong Kong. Psychol Heal Med. 2004;9: 286–292. doi:10.1080/13548500410001721855

153. Yip YB, Sit JW, Fung KKY, Wong DYS, Chong SYC, Chung LH, et al. Impact of an arthritis self-management programme with an added exercise component for osteoarthritic knee sufferers on improving pain, functional outcomes, and use of health care services: An experimental study. Patient Educ Couns. 2007;65: 113–121. doi:10.1016/j.pec.2006.06.019

154. McGowan P, Green LW. Arthritis Self-management in Native Populations of British Columbia: An Application of Health Promotion and Participatory Research Principles in Chronic Disease Control. Can J Aging / La Rev Can du Vieil. 1995;14: 201–212. doi:10.1017/S0714980800005511

155. Georges CA, Bolton LB, Bennett C. Functional health literacy: an issue in African-American and other ethnic and racial communities. J Natl Black Nurses Assoc. 2004;15: 1–4. Available: https://europepmc.org/article/med/15712814

156. Registered Nurses Association of Ontario. Client Centred Care: Revision supplement. Toronto, Canada; 2006.

157. Deakin TA, Cade JE, Williams R, Greenwood DC. Structured patient education: the Diabetes X-PERT Programme makes a difference. Diabet Med. 2006;23: 944–954. doi:10.1111/J.1464-5491.2006.01906.X

158. Pepper-Burke N. Chronic Disease Self-Management Workshop: An Implementation Model From Southern California. Health Promot Pract. 2003;4: 8–13. doi:10.1177/1524839902238281

159. Lefort SM, Gray-Donald K, Rowat KM, Jeans ME. Randomized controlled trial of a community-based psychoeducation program for the self-management of chronic pain. Pain. 1998;74: 297–306. doi:10.1016/S0304-3959(97)00190-5

160. Karlsson MR, Edner M, Henriksson P, Mejhert M, Persson H, Grut M, et al. A nurse-based management program in heart failure patients affects females and persons with cognitive dysfunction most. Patient Educ Couns. 2005;58: 146–153. doi:10.1016/j.pec.2004.08.005

161. Coleman EA, Smith JD, Frank JC, Min SJ, Parry C, Kramer AM. Preparing Patients and Caregivers to Participate in Care Delivered Across Settings: The Care Transitions Intervention. J Am Geriatr Soc. 2004;52: 1817–1825. doi:10.1111/J.1532-5415.2004.52504.X

162. Glasgow R, Toobert D, Barrera M, Strycker LA. Assessment of Problem-Solving: A Key to Successful Diabetes Self-Management. J Behav Med 2004 275. 2004;27: 477–490. doi:10.1023/B:JOBM.0000047611.81027.71

163. Bray P, Roupe M, Young S, Harrell J, Cummings DM, Whetstone LM. Feasibility and effectiveness of system redesign for diabetes care management in rural areas: The Eastern North Carolina experience. Diabetes Educ. 2005;31: 712–718. doi:10.1177/0145721705280830

164. Coleman M, Newton K. Supporting self-management in patients with chronic illness. Am Fam Physician. 2005;72: 1503–1510.

165. Keers JC, Blaauwwiekel EE, Hania M, Bouma J, Scholten-Jaegers SMHJ, Sanderman R, et al. Diabetes rehabilitation: development and first results of a Multidisciplinary Intensive Education Program for patients with prolonged self-management difficulties. Patient Educ Couns. 2004;52: 151–157.

166. Bodenheimer T. Interventions to Improve Chronic Illness Care: Evaluating Their Effectiveness. http://www.liebertpub.com/dis. 2003;6: 63–71. doi:10.1089/109350703321908441

167. Morgan U. The introduction of collaborative care plans. Prof nurse. 1997;12: 556–558.

168. Funnell MM, Anderson RM. Empowerment and self-management of diabetes. Clin Diabetes. 2004;22: 123–128. Available: https://go.gale.com/ps/i.do?p=AONE&sw=w&issn=08918929&v=2.1&it=r&id=GALE%7CA120103231&sid=googleScholar&linkaccess=fulltext

169. Mensing C, Boucher J, Cypress M, Weinger K, Mulcahy K, Barta P, et al. National Standards for Diabetes Self-Management Education. Diabetes Care. 2002;25: s140–s147. doi:10.2337/DIACARE.25.2007.S140

170. Fisher EB, Brownson CA, O’Toole ML, Shetty G, Anwuri V V., Glasgow RE. Ecological approaches to self-management: The case of diabetes. Am J Public Health. 2005;95: 1523–1535. doi:10.2105/AJPH.2005.066084

171. Jack L, Liburd L, Spencer T, Airhihenbuwa CO. Understanding the environmental issues in diabetes self-management education research: A reexamination of 8 studies in community-based settings. Ann Intern Med. 2004;140: 964–971. doi:10.7326/0003-4819-140-11-200406010-00038

172. Heisler M. Helping your patients with chronic disease: Effective physician approaches to support self-management. Hosp Physician. 2005;41: 45.

173. Jayasuriya P, Shaw E, Roach S, Bailey L. Self management for chronic disease: an introduction. Aust Fam Physician. 2001;30: 913–916.

174. Holland SK, Greenberg J, Tidwell L, Newcomer R. Preventing Disability Through Community-Based Health Coaching. J Am Geriatr Soc. 2003;51: 265–269. doi:10.1046/J.1532-5415.2003.51068.X

175. Wagner E, Davis C, Schaefer J, Von Korff M, Austin B. A survey of leading chronic disease management programs: are they consistent with the literature? Manag Care Q. 1999;7: 56–66. Available: https://europepmc.org/article/med/10620960

176. Wilson K, Coulon L, Hillege S, Swann W. Nurse practitioners’ experiences of working collaboratively with general practitioners and allied health professionals in New South Wales, Australia. Aust J Adv Nursing,. 2005;23.

177. Loeb SJ, Penrod J, Falkenstern S, Gueldner SH, Poon LW. Supporting older adults living with multiple chronic conditions. West J Nurs Res. 2003;25: 8–23. doi:10.1177/0193945902238830

178. Rauscher C. Self-management Support Design Template and Discussion. 2006.

179. College of Nurses of Ontario. Quality Assurance. 2010. Available: https://www.cno.org/en/myqa/

180. Canadian Nurses Association. CNA Backgrounder. 2005.

181. Funnell MM, Brown TL, Childs BP, Haas LB, Hosey GM, Jensen B, et al. National standards for diabetes self-management education. Diabetes Educ. 2007;33: 599–600, 602–4, 606 passim. doi:10.1177/0145721707305880

182. Mensing C, Boucher J, Cypress M, Weinger K, Mulcahy K, Barta P, et al. National Standards for Diabetes Self-Management Education. Diabetes Care. 2006;29: s78–s85. doi:10.2337/DIACARE.29.S1.06.S78

183. Sadur CN, Moline N, Costa M, Michalik D, Mendlowitz D, Roller S, et al. Diabetes management in a health maintenance organization. Efficacy of care management using cluster visits. Diabetes Care. 1999;22: 2011–2017. doi:10.2337/DIACARE.22.12.2011

184. Dickey LL, Gemson DH, Carney P. Office system interventions supporting primary care–based health behavior change counseling. Am J Prev Med. 1999;17: 299–308. doi:10.1016/S0749-3797(99)00083-5

185. Registered Nurses’ Association of Ontario. Toolkit: Implementation of clinical practice guidelines. 2nd ed. Toronto, Canada; 2006.
